# The exhaustive genomic scan approach, with an application to rare-variant association analysis

**DOI:** 10.1038/s41431-020-0639-3

**Published:** 2020-05-15

**Authors:** George Kanoungi, Michael Nothnagel, Tim Becker, Dmitriy Drichel

**Affiliations:** 1 Faculty of Medicine and University Hospital Cologne, Cologne Center for Genomics (CCG), University of Cologne, Weyertal 115b, 50931 Cologne, Germany; 2 Institute for Community Medicine, Ernst Moritz Arndt University Greifswald, Greifswald, Germany; 3 xValue GmbH, Willich, Germany; 4 Drichel Analytics, Alexanderstraße 6, 53111 Bonn, Germany

**Keywords:** Genetics, Psychiatric disorders, Immunological disorders, Data processing

## Abstract

Region-based genome-wide scans are usually performed by use of a priori chosen analysis regions. Such an approach will likely miss the region comprising the strongest signal and, thus, may result in increased type II error rates and decreased power. Here, we propose a genomic exhaustive scan approach that analyzes all possible subsequences and does not rely on a prior definition of the analysis regions. As a prime instance, we present a computationally ultraefficient implementation using the rare-variant collapsing test for phenotypic association, the genomic exhaustive collapsing scan (GECS). Our implementation allows for the identification of regions comprising the strongest signals in large, genome-wide rare-variant association studies while controlling the family-wise error rate via permutation. Application of GECS to two genomic data sets revealed several novel significantly associated regions for age-related macular degeneration and for schizophrenia. Our approach also offers a high potential to improve genome-wide scans for selection, methylation, and other analyses.

## Introduction

Genomic scans assess genomic regions (usually subsequences) with respect to some statistical measure and, ideally, quantify its consistency with the null hypothesis. Prominent applications include the detection of allele frequency differences between cases and controls in genetic association studies [1], the departure of the site-frequency spectrum (SFS) from the expectation under neutral evolution in selection analysis [2] and of differential methylation patterns in epigenomics [3]. Although statistical tests differ, the basic procedure remains similar across these applications by comprising [1] the prior definition of a set of contiguous analysis regions (bins) *B*_*ij*_, characterized by start positions *i* and end positions *j* (“binning”), sometimes defined by setting scanning parameter values (“sliding window”) [2]; the calculation of a suitable summary or test statistic, T(*B*_*ij*_), for each bin [3]; the distributional assessment of the statistics in order to identify extreme values, frequently including the calculation of *p* values, and often, but not always, followed by control of the family-wise error rate (FWER).

With long chromosomal sequences, it is not known in advance which subset of possible subsequences is most suitable for statistical summarization and testing, i.e., which regions will provide the highest power. Use of a priori fixed regions, including sliding-window approaches with fixed bins, will result in a highly likely increase in the type II error rate and, correspondingly, reduced power, because regions comprising the strongest signal(s) will almost certainly not be chosen prior to the analysis. A more probable scenario is that a region of interest will only partially coincide with the chosen analysis region. As a consequence, the signal will be diluted by inclusion of nonrelevant variants, split across multiple analysis regions, or both. Fixed, predetermined binning therefore represents a major limitation of current genomic scans. Moreover, due to unknown correlation structures between regions, the correction for multiple testing is often performed in a conservative way, e.g., by use of Bonferroni correction for the number of tested regions [4].

Here, we focus on the application of the exhaustive scan approach to rare-variant (RV) association studies based on sequenced or genotyped data. RV analysis is motivated by the observation that although genome-wide association studies (GWAS) have usually identified common risk alleles for a wide range of complex diseases [5], most of these alleles cause at most moderate increases in risk and contribute little to the overall heritability of diseases individually, leaving large portions of human diseases’ heritability unexplained [5, 6]. This observation motivated studies to focus on the role of RVs, aiming to deliver functionally interpretable variants of moderate-to-large effect sizes and explaining additional disease risk variability. Region-based RV association analyses are based on the assumption that multiple RVs in physical proximity have similar effects on the phenotype. Under this assumption, multiple RVs in a genomic region can be aggregated and analyzed as a unit. In this context, the most common approach is to define fixed bins by either using the locations of known protein-coding genes as regions of analysis or by using a sliding-window approach with two fixed parameters, namely the window size and the step size. Either choice is fundamentally limited in scope, and will consider only a tiny fraction of possible subsequences.

In RV analysis, “rareness” itself is another parameter that is usually defined by a threshold of the minor allele frequency, MAF_*T*__._ Alternatively, weighting schemes have been proposed that assign lower weights to variants with higher allele counts. This does not fully solve the problem of rareness thresholds, as the shape of the weighting function is usually chosen somewhat arbitrarily and without a stringent justification of its usefulness.

Noteworthy progress towards non-parametric RV analysis has been made in [7], who proposed the Variable-Threshold (VT) approach, in which test statistics for all possible MAF_*T*_ are computed and the optimal MAF_*T*_ is adapted from the data. The method uses permutation testing to adjust for the large number of tested hypotheses within a bin; it is therefore computationally more intense. In [8], the VT method was extended to the collapsing and the CMAT tests [9], whereas the method became computationally impractical for regression models.

However, even if the problem of the unknown “rareness” can be alleviated, the problem of the choice of analysis regions remains, which has been acknowledged before [10–13]. The present work can be regarded as the extension of the VT method to binning of analysis regions (“variable binning”).

Here, we suggest to perform an exhaustive scan for phenotypic association using a simple RV test (collapsing method, COLL) as the test statistic [14]. COLL dichotomizes samples by their carrier status, i.e., whether the corresponding individual is carrying at least one rare allele in the analysis region. In a case–control study design, a 1-df χ^2^-test can be applied to the resulting 2 × 2 contingency table. Interestingly, despite its relatively simple disease model, the power of COLL is comparable with more sophisticated methods for a wide range of disease models [9]. However, COLL is inherently limited in that it can only be applied to binary phenotypes only, does not account for covariates, and has limited power if the associated RVs in the region have different effect directions. A large number of more advanced tests have been developed, see [8, 15, 16] for categorizations. A notable example is the sequence kernel association test (SKAT) [17], which is a variance-component test and sensitive to mixed effect directions in a region, allows for inclusion of covariates, and can be used with binary and quantitative phenotypes.

**Fig. 1:**
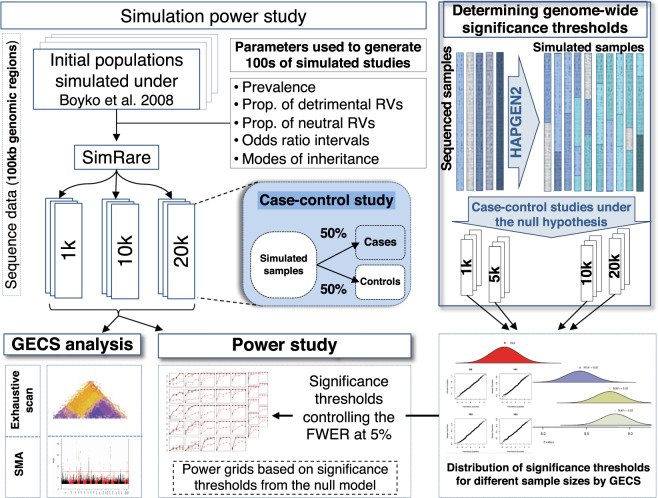
Workflow chart of the entire simulation study illustrating the major steps and procedures. See main text for details.

Here, we propose the use of exhaustive scans to all possible contiguous subsequences and to perform multiple-testing correction by obtaining the distribution of extreme *p* values from replicates of the data simulated under the null hypothesis by repeatedly permuting case–control status. We introduce this approach, in an exemplary way, for a specific application, namely the genomic exhaustive collapsing scan (GECS) approach for COLL, and present a computationally efficient implementation of GECS. We show that although the number of possible contiguous bins for all RVs at a single chromosome is very large, namely *n*(*n* + 1)/2 with *n* variants, the number of distinct bins dramatically reduces by about three to four orders of magnitude, rendering GECS feasible and scalable even for whole-genome sequence data in large sample sets. Furthermore, this acceleration allows control of the FWER via repeated case–control status permutation that provides optimal power to detect association [18]. Based on simulations, we derive empirical thresholds for genome-wide significance in case–control WGS studies for different sample sizes and minor allele frequency thresholds, in an approach analogous to [19]. In applying GECS to two real-world data sets, we show that our approach is feasible and scalable with large, modern association studies and provides a fine-grained, base-pair resolution of associated regions contained in the data (Fig. 1), which will enable a deeper understanding of the effect of RVs on the etiology of complex diseases.

## Methods

### GECS algorithm

Our method is based on the observation that under the collapsing test COLL (see Supplementary Note a), test statistics for all locally distinct bins can be computed efficiently without explicit computation of each bin. In pseudocode, the algorithm can be formulated as follows:

$${\mathrm{for}}\ 	\left( {i = 0; 	i\, < \, n;i{ + + }} \right)\left\{ \, \right.\\ 	{\mathrm{for}}\ \left( {j = i;{\mathrm{ }}j \, < \, n;{\mathrm{ }}j{ + + }} \right)\left\{ \, \right.\\ 	\hskip 20pt {\mathrm{if}}\ \left( {{\boldsymbol{B}}_{{\boldsymbol{ij}}} = = {\boldsymbol{B}}_{\left( {{\boldsymbol{i}} + 1} \right){\boldsymbol{j}}}\parallel {\boldsymbol{B}}_{{\boldsymbol{ij}}} = = {\mathbf{1}}} \right){\mathrm{break}};\\ 	\hskip 20pt {\mathrm{else}}\ {\mathrm{if}}\ \left( {{\boldsymbol{B}}_{{\boldsymbol{ij}}} = = {\boldsymbol{B}}_{{\boldsymbol{i}}\left( {{\boldsymbol{j}} + 1} \right)}} \right)\ {\mathrm{continue}};\\ 	\hskip 20pt {\mathrm{else}}\ {\mathrm{compute}}\ {\boldsymbol{T}}\left( {{\boldsymbol{B}}_{{\boldsymbol{ij}}}} \right);\ //\, {\mathrm{locally}}\, {\mathrm{distinct}}\, {\mathrm{bin}}\, {\mathrm{identified}}\\ 	\hskip 20pt {\mathrm{\} }}\\ 	\hskip -15pt {\mathrm{\} }}$$

Here, *n* is the number of variants on a linear chromosome, *B*_*ij*_ is the set of carriers of a minor allele (which can be conveniently parametrized by a binary array) and *T(B*_*ij*_*)* is the corresponding test statistic. See Supplementary Notes b–d for a more detailed justification and description of the algorithm.

### Simulation studies

We performed extensive simulation studies (Fig. 1) to (i) determine genome-wide significance thresholds for region-agnostic RV testing (Table 1), (ii) assess the statistical power of our approach (Figs. 2, 3 and S5–S12), and (iii) benchmark the feasibility of GECS for analysis of large genomic data sets (see Supplementary Note f) for the description of the studies and (Supplementary Note g) for the results.

**Table 1:** Empirical, sample-size dependent significance thresholds (*α*, with control of the FWER at 5%) for simulated genome-wide studies.

Sample size	Number of replications	SMA	GECS, 3 MAF_*T*_ combined	GECS, MAF_*T*_ = 0.01	GECS, MAF_*T*_ = 0.03	GECS, MAF_*T*_ = 0.05
1000	1000	2.95 × 10^−8^	7.35 × 10^−10^	3.61 × 10^−9^	1.73 × 10^−9^	1.60 × 10^−9^
5000	1000	1.86 × 10^−8^	3.31 × 10^−10^	1.26 × 10^−9^	8.92 × 10^−10^	8.49 × 10^−10^
10,000	1000	1.27 × 10^−8^	2.81 × 10^−10^	1.05 × 10^−9^	7.13 × 10^−10^	6.91 × 10^−10^
20,000	500	1.15 × 10^−8^	2.59 × 10^−10^	9.28 × 10^−10^	6.36 × 10^−10^	6.01 × 10^−10^

**Fig. 2:**
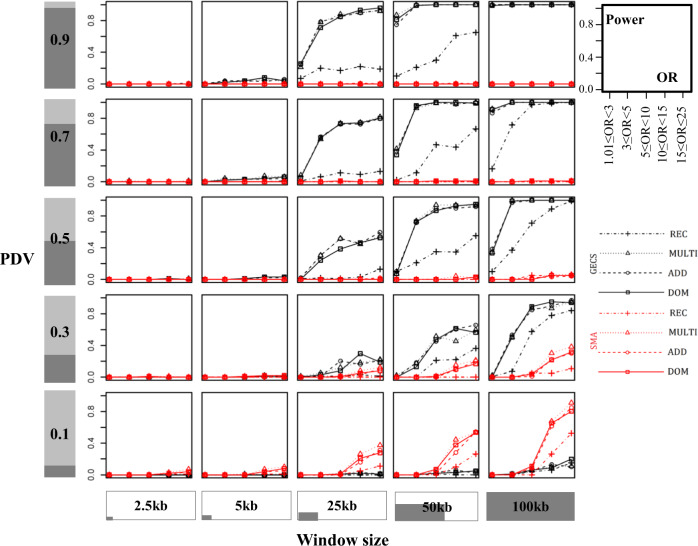
Comparative power analysis for a rare disease (prevalence *K* = 0.01) and small sample size (*N* = 1000). Results are given for studies with proportion of neutral rare variants (PNV) = 0.3, different simulated window sizes (*x*-axis), and different proportions of detrimental rare variants (PDV) (*y*-axis). Black lines: GECS; red lines: SMA. In each grid cell, the power is presented on the *y*-axis and OR intervals on the *x*-axis. For an overview see Table S20.

**Fig. 3:**
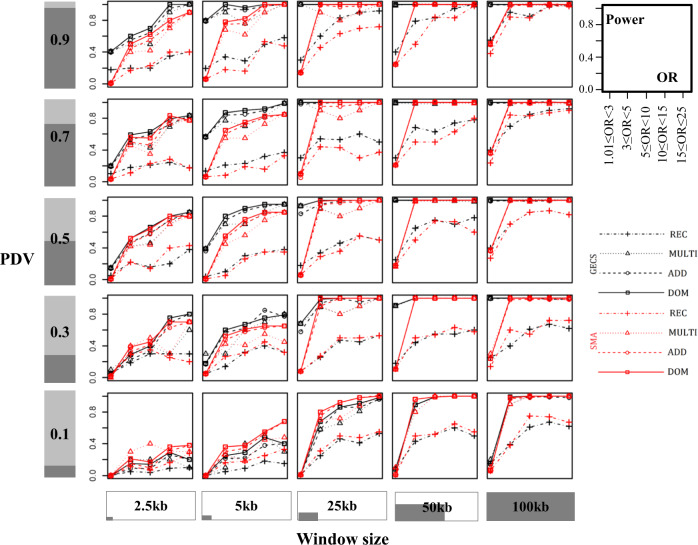
Comparative power analysis for a rare disease (prevalence *K* = 0.01) and moderate sample size (*N* = 10,000). Results are given for studies with proportion of neutral rare variants (PNV) = 0.3, different simulated window sizes (*x*-axis), and different proportions of detrimental rare variants (PDV) (*y*-axis). Black lines: GECS; red lines: SMA. In each grid cell, the power is presented on the *y*-axis and OR intervals on the *x*-axis. For an overview see Table S20.

### Real-world data set analysis

Advanced age-related macular degeneration (AAMD) GWAS from the International AMD Genomics Consortium (dbGaP accession: phs001039.v1.p1) and schizophrenia (SCZD) exome sequencing study from a population-based schizophrenia Swedish case-control cohort (dbGaP accession: phs000473.v2.p2). We validated the most interesting bins by performing association testing with SKAT (*p*′ values, see Supplementary Note h). For the description of the data sets, the quality control, and the analysis setup see Supplementary Note e and h.

## Results

### Real-data analysis

#### Advanced age-related macular degeneration (AAMD)

We applied GECS to the whole-genome imputed case–control data of the subset of samples with European ancestry and cases with AAMD (Table S3). The strongest signals were detected in bins overlapping with protein-coding genes, including human leukocyte antigen B (*HLA-B*), *HLA-DRA*, and *MICB* in chromosome 6, *FYB* in chromosome 5, *CFD*, and *NRTN* in chromosome 9, and *PLEKHA1* in chromosome 10 (Table S10). These genes, among others, are involved in the regulation of the immune system process and innate immune response. The set of genes overlapping significant bins were enriched in the activation of immune response pathway, in particular, the positive regulation of immune response (7.36-fold enrichment, Bonferroni-corrected *p* value of 4.4 × 10^−4^; see Table S11). In addition, GECS reidentified and refine most of the previously reported RV associations with AAMD (e.g., *CFI*, *C3*, *SKIV2L*, *SYN3*, and *C9*) (Table S12) [20, 21]. Odds ratios of identified bins ranged between 0.5 and 3.45, indicating that carrier status can be both positively and negatively correlated with AAMD. Significant bins with OR > 1 were overrepresented on chromosome 6, with OR values ranging between 1.1 and 1.4 and bin sizes ranging between 2 and 26 rare variants.

Notably, bin 6.I (chr6: 31,323,455–31,323,745 bp, hg19) of 12 rare variants (MAF ≤ 0.05) was found to be significant with a *p* value of 3.48 × 10^−10^, *p*′ value of 2.76 × 10^−11^, and OR of 1.18 [1.12, 1.24]. This bin overlaps with the protein-coding *HLA-B*, which plays a very important role in the immune system (Fig. S17). Interestingly, a previous study found a positive correlation between the *HLA-B* allele HLA-B27 with AAMD [22]. Also, bin 6.II (chr6: 31,473,707–31,474, 883 bp) overlapped with the *MICB* gene and comprised six rare variants (MAF ≤ 0.05). This bin was found to be significantly associated with AAMD with *p* value 1.71 × 10^−10^, *p*′ value of 2.08 × 10^−13^, and OR = 1.27 [1.19, 1.38] (Fig. S18). An example for a bin with OR < 1 is 10.I (chr10: 124,226,492–124,249,185 bp), which comprised 64 rare variants (MAF ≤ 0.05), was found to be associated with AAMD with *p* value of 2.09 × 10^−84^, *p*′ value of 2.96 × 10^−30^, and OR = 0.62 [0.59, 0.65]. Notably, this bin, with an apparently protective effect of rare alleles overlaps with *HTRA1*, which has been functionally studied in the context of AMD [23]. The association signal was independent from multiple common variants found to be associated with AAMD in this gene [24, 25]. Another noteworthy finding was bin 6.IV (chr6: 31,878,006–31,878,721 bp) with five rare variants in the *C2* gene (MAF ≤ 0.05) was found to be significantly associated with AMD with *p* value of 3.78 × 10^−80^, *p*′ value of 1.23 × 10^−70^, and OR = 0.53 [0.50, 0.57] (Fig. S19). Our finding is in line with the known role of some protective haplotypes in the *C2*-*AS1* region were found to be significantly reducing the risk of AMD [26]. For more results, see Supplementary Note i.

#### Schizophrenia

We applied GECS to the WES variant data (Table S5). The analysis was conducted with three MAF thresholds, and the genome-wide significance threshold in the combined study comprised 1.87 × 10^−08^ (Tables 2, Figs. S21–24). Most of the alleles identified to be significantly associated to schizophrenia had OR < 1, so that the carrier status appeared to be protective (Table 3, S15). For example, bins like 15.I (chr15: 73,044,829–73,044,833 bp), 17.I (chr17: 49,239,143–49,239,143 bp), and 22.I (chr22: 17,687,954–17,688,129 bp) overlapped with genes on chromosome 15 (*ADPGK*), 17 (*NME1*, *NME2*), and 22 (*CERC1*) (Table S16). These genes are involved in the purine nucleoside triphosphate biosynthetic process, which has previously been demonstrated as to be strongly linked to the development of schizophrenia [27]. On the other hand, bin 19.I (chr9: 8,999,386–9,028,410 bp), comprising 62 rare SNPs (MAF ≤ 0.03), was found to be significant, with *p* value 2.59 × 10^−09^, *p*′ value 3.11 × 10^−10^, and OR = 1.29 [1.19, 1.40], covering exonic regions of the *MUC16* gene (Fig. S25). Although some rare alleles in *MUC16* were reported in association to schizophrenia, none of the 62 rare alleles in this bin were reported before. Moreover, genes covered by bins 15.I, 17.I, 19.I, and 22.I were found to have a function in the small molecule metabolic processes. Interestingly, gene *PRSS3* was covered by bin 9.I (9: 33,796,672–33,798,630) comprising 20 rare variants (MAF ≤ 0.05), *p*′ value of 3.89 × 10^−11^, and OR = 1.37 [1.24, 1.52]. This gene was not previously reported to be related to schizophrenia. The relatively small sizes of the detected significant bins in the WES data of schizophrenia indicate that the availability of large whole-genome sequencing studies will enable a considerable power gain for our method (Table S17).

**Table 2:** Significance thresholds (*α*, with control of the FWER at 5%) for the whole-genome, imputed AAMD data set, and the whole-exome SCZD data set.

Data set	SMA	GECS, 3 MAF_*T*_ combined	GECS, MAF_*T*_ = 0.01	GECS, MAF_*T*_ = 0.03	GECS, MAF_*T*_ = 0.05
AAMD	1.81 × 10^−8^	1.43 × 10^−9^	7.42 × 10^−9^	2.80 × 10^−9^	2.54 × 10^−9^
SCZD	8.32 × 10^−7^	1.87 × 10^−8^	4.35 × 10^−8^	3.59 × 10^−8^	2.84 × 10^−8^

**Table 3:** A selection of bins with the locally most significant association signals in AAMD and SCZD data sets, detected by GECS and verified by SKAT.

	Chr.	Bin position (hg19)		Gene	MAF_*T*_	#RVs	OR [95% CI]	*p* value	*p*′ value
AAMD	6	31,935,392	31,937,762	*DXO, SKIV2L*	0.03	28	0.55 [0.52, 0.59]	6.24 × 10^−76^	4.74 × 10^−81^
	6	31,878,006	31,878,721	*C2*	0.05	5	0.53 [0.50, 0.57]	3.78 × 10^−80^	1.23 × 10^−70^
	10	124,226,492	124,249,185	*HTRA1*	0.05	64	0.62 [0.59, 0.65]	2.09 × 10^−84^	2.96 × 10^−30^
	19	6,718,146	6,718,155	*C3*	0.03	2	2.98 [2.42, 3.69]	7.87 × 10^−27^	6.30 × 10^−28^
	6	31,473,707	31,474,883	*MICB*	0.05	6	1.27 [1.19, 1.38]	1.71 × 10^−10^	2.08 × 10^−13^
	6	31,323,455	31,323,745	*HLA-B*	0.05	12	1.18 [1.12, 1.24]	3.48 × 10^−10^	2.76 × 10^−11^
	4	110,685,721	110,685,820	*CFI*	0.01	5	3.42 [2.34, 5.04]	2.15 × 10^−11^	7.03 × 10^−10^
	6	31,373,445	31,373,957	*MICA*	0.05	9	1.29 [1.20, 1.39]	5.74 × 10^−12^	1.03 × 10^−09^
	5	39,199,134	39,199,134	*FYB*	0.03	1	1.75 [1.47, 2.08]	2.40 × 10^−10^	1.70 × 10^−10^
	5	39,327,884	39,327,888	*C9*	0.03	2	1.75 [1.48, 2.03]	4.58 × 10^−12^	1.28 × 10^−11^
**SCZD**	9	33,796,672	33,798,630	*PRSS3*	0.05	20	1.37 [1.24, 1.52]	5.07 × 10^−10^	3.89 × 10^−11^
	15	73,044,829	73,044,833	*ADPGK*	0.03	2	0.42 [0.36, 0.52]	1.17 × 10^−19^	4.30 × 10^−20^
	17	49,239,143	49,239,143	*NME1.NME2*	0.01	1	0.13 [0.06, 0.29]	2.72 × 10^−09^	5.58 × 10^−11^
	19	8,999,386	9,028,410	*MUC16*	0.03	62	1.29 [1.19, 1.40]	2.59 × 10^−09^	3.10 × 10^−10^
	22	17,687,954	17,688,129	*CERC1*	0.01	9	0.27 [0.19, 0.40]	3.79 × 10^−13^	6.77 × 10^−14^

Each bin is the most significant signal in the block of all overlapping significant bins detected by GECS. These bins are verified by SKAT, adjusted for sex, age, ten principal components, and common variants in physical proximity, if available (p′ values). For verification with SKAT, we set the threshold at 5 × 10^−8^ for AAMD and 2 × 10^−6^ for SCZD. See Supplementary Material for more comprehensive results.

## Discussion

While genome-wide scans with heuristically predetermined analysis regions are an established approach, they are limited in their scope, resolution, and power by requiring a prior choice of the analysis regions. In the context of selection analysis, Akey fittingly compared the scan with a hatchet and called for more refined scalpel-like approaches [28]. We argue that in Akey’s analogy, the exhaustive scan is an electron microscope, as it allows for base-pair-level analysis of genomic regions, with genome-wide, nonconservative, optimally powerful correction for multiple testing using replicates of the data generated under the null hypothesis.

GECS is scalable to large association studies of imputed and sequenced variant data, as demonstrated by our simulation of the null model. The efficiency of our implementation allowed us to estimate significance thresholds for RV analysis in whole-genome sequenced data for association studies comprising up to 20,000 individuals. As a by-product, the analysis offered another opportunity to study significance thresholds (FWER control at 5%) for single-marker analysis (SMA), which, even for small sample sizes of *N* = 1000, was found to be stricter (*α* = 2.95 × 10^−8^) than the commonly used threshold of *α* = 5.0 × 10^−8^. This result is consistent with previously published results [19, 29] and highlights the need to abandon the “agreed-upon” significance threshold of 5.0 × 10^−8^, which is anticonservative for large-scale association studies.

The estimates of *α* allow us to assess the absolute power of the region-based exhaustive scan in future whole-genome deeply sequenced data sets. In contrast to previous studies [9], the power study is free from the assumption that the simulated region and the analysis region happen to coincide. Since the exhaustive scan is guaranteed to identify the most strongly associated regions, our FWER control accounts for the multiple-testing “cost” of finding these regions, which was ignored in previous studies. Overall, the power of GECS is higher, or at least comparable with SMA for small to moderate odds ratios of associated rare variants (1.01 ≤ OR < 3), being the OR range expected to be most commonly found in complex diseases. For large sample sizes and large effect sizes, GECS, in general, offers no advantage to detect association. This result reflects the expectation that given a large sample size, enough rare alleles will be present to detect associated variants with sufficient power in single-variant tests [30].

We applied GECS to real-world data sets, namely of AAMD (imputed microarray data) and of schizophrenia (WES), and performed very stringent quality control of both sets to avoid possible type I errors. Application of GECS to AAMD confirmed a multitude of previously reported rare associated SNPs, for which SMA was underpowered to pick up many signals due to the low MAFs. We confirmed that exhaustively scanning for association through all possible combinations of contiguous rare variants from different MAF thresholds alleviates the limitations posed by previous fixed-bin strategies. The in-depth follow-up analysis showed high enrichment of genes covered by identified bins in pathways with key roles in the development and function of immune system. Our approach was also successfully applied to the schizophrenia data set, however, judging by the limited spatial extent of the resulting bins, the approach might be underpowered due to limited coverage of the genome in WES studies and will probably improve with availability of WGS data.

GECS is a powerful approach for detecting phenotypic association of genomic regions harboring rare variants and for refining our understanding of their contribution to predisposition for complex diseases. We conclude that our approach is well-suited for whole-genome and whole-exome association analyses. However, GECS utilizes the simple allele counting function of COLL to achieve perfect, essentially base-pair-level spatial resolution. As COLL is only able do dichotomize individuals by the carrier status, the test is not able to distinguish between carriers of one or more minor alleles. We alleviated the limitations of COLL by performing follow-up analysis of candidate regions with locally exhaustive scans using SKAT. Enabling the exhaustive scan with more sophisticated tests that take more sources of information into account, like allele counts and covariates, might reveal further associated candidate regions. The challenge of extending the exhaustive scan approach to more complex association tests is purely computational in nature. Our algorithm does not generalize to other published association tests in a straightforward manner, so that new solutions will be required to generalize the exhaustive association scan beyond the collapsing method.

Application of exhaustive scans is not limited to association testing and could be useful in further applications, in particular for studying methylation and evolutionary selection. In fact, our preliminary results show that the exhaustive scan is feasible for the study of selection when used with SFS-based tests such as Tajima’s D (data not shown). This is due to the fact that the computational complexity of SFS-based tests is independent from the number of individuals in the study, since only allele count data is required. As a consequence, the quadratic space of all contingent regions can be computed by brute force, even for very large data sets. Moreover, modern, efficient coalescent simulators such as msprime [31] and fastsimcoal2 [32] can be used to simulate the null model under neutral evolution under realistic demographic histories [33], which can be used for FWER-controlled *p* values.

In summary, we developed a method that allows for an exhaustive scan of all possible contiguous genomic regions with the collapsing test and eliminates the choice of candidate bins. Instead, the space of all possible bins is tested. This eliminates binning as a source of type II error and is expected to improve power. Furthermore, the speed-up by several orders of magnitude allows for computation of nonconservative genome-wide significance thresholds by permutation, leading to improved power when compared with conservative correction methods such as Bonferroni’s. We show that GECS indeed improves statistical power in both simulated and empirical data sets.

## Supplementary information

TheExhaustiveGenomicScan_Supplementary_Revision2

External_data

## Data Availability

The software is written in C++ and is available at https://github.com/ddrichel/GECS.
