# Supplementary material for: The exhaustive genomic scan approach, with an application to rare-variant association analysis
Source: Eur J Hum Genet. 2020 May 15;28(9):1283–91. doi: 10.1038/s41431-020-0639-3 (PMC7608423; doi:10.1038/s41431-020-0639-3)
Supplement: Supplementary file 1 — TheExhaustiveGenomicScan_Supplementary_Revision2 [file 41431_2020_639_MOESM1_ESM.docx]

**The exhaustive genomic scan approach, with an application to rare variant association analysis**

George Kanoungi, Michael Nothnagel, Tim Becker, Dmitriy Drichel

# Supplement

## Tables:

**Table S1: Values of parameters which were used in the power study. Each possible combination of parameters represented a scenario in the study, except for simulations with N=20,000, which were performed for PNV=0.3 and the dominant inheritance mode only**

| **Parameters** | **Values** |
| --- | --- |
| Prevalence (K) | 0.01, 0.1 |
| PNV | 0.1, 0.3 |
| Sample size | 1,000, 10,000, 20,000 |
| Inheritance mode | dominant (DOM), additive (ADD),  multiplicative (MULTI), recessive (REC) |
| PDV | 0.1, 0.3, 0.5, 0.7, 0.9 |
| OR intervals | 1.01-3, 3-5, 5-10, 10-15, 15-25 |
| Size of analysis window | 2.5kb, 5kb, 25kb, 50kb, 100kb |

**Table S2: Description of the AMD data set (build hg19)**

| **Number of samples** | **All samples** | **European ancestry** | **African ancestry** | **Asian ancestry** | **Unknown ancestry** |
| --- | --- | --- | --- | --- | --- |
|  | 35,358 | 32,637 | 208 | 1,742 | 771 |
| cases | 19,112 | 18,047 | 76 | 649 | 340 |
| controls | 16,246 | 16,106 | 132 | 1093 | 431 |
| women | 20,214 | 18,829 | 890 | 85 | 410 |
| men | 15,144 | 13,808 | 852 | 123 | 361 |

**Table S3: Description of the European AMD data set, used for further analysis**

|  | **All data set** | **European** | **European after QC*** |
| --- | --- | --- | --- |
| **Sample size** | 35,358 | 32,637 | 27,259 |
| **Controls** | 16,246 | 14,590 | 14,566 |
| **Advanced AMD (AAMD) cases** | 13,379 | 12,711 | 12,693 |
| **Intermediate AMD cases** | 5,733 | 5,336 | - |
| **All AMD cases** | 19,112 | 18,047 | 12,693 |
| **Average age** | ~75 | ~77 | ~72 |
| **Number of SNPs** | 11,722,963 | 11,712,893 | 11,712,830 ** |

* In the quality control stage, intermediate AMD cases and 42 PCA-outliers (see table S4) were excluded.

** Only 428,357 variants were directly genotyped in the data set.

**Table S4: Excluded PCA-outliers in the AMD data set.**

| **Nr.** | **Sample-ID** |
| --- | --- |
| 1 | IAMDGC00769 |
| 2 | IAMDGC05427 |
| 3 | IAMDGC06555 |
| 4 | IAMDGC06574 |
| 5 | IAMDGC08194 |
| 6 | IAMDGC08279 |
| 7 | IAMDGC09965 |
| 8 | IAMDGC11776 |
| 9 | IAMDGC13070 |
| 10 | IAMDGC14221 |
| 11 | IAMDGC14509 |
| 12 | IAMDGC14534 |
| 13 | IAMDGC14967 |
| 14 | IAMDGC15779 |
| 15 | IAMDGC16012 |
| 16 | IAMDGC16119 |
| 17 | IAMDGC16223 |
| 18 | IAMDGC16753 |
| 19 | IAMDGC17010 |
| 20 | IAMDGC18277 |
| 21 | IAMDGC18598 |
| 22 | IAMDGC18648 |
| 23 | IAMDGC19367 |
| 24 | IAMDGC22398 |
| 25 | IAMDGC24181 |
| 26 | IAMDGC25735 |
| 27 | IAMDGC25908 |
| 28 | IAMDGC26258 |
| 29 | IAMDGC26643 |
| 30 | IAMDGC26652 |
| 31 | IAMDGC26702 |
| 32 | IAMDGC28886 |
| 33 | IAMDGC29369 |
| 34 | IAMDGC31174 |
| 35 | IAMDGC32617 |
| 36 | IAMDGC32793 |
| 37 | IAMDGC32830 |
| 38 | IAMDGC33953 |
| 39 | IAMDGC34220 |
| 40 | IAMDGC34288 |
| 41 | IAMDGC34749 |
| 42 | IAMDGC35085 |

**Table S5: Description of the SCZD data set (build hg19).**

| **Applied**  **QC-level** | **Cases** | **Controls** | **Female** | **Male** | **Total** | **Total number of variants** |
| --- | --- | --- | --- | --- | --- | --- |
| None | 6,135 | 6,245 | 5,780 | 6,600 | 12,380 | 1,811,204 |
| SCZD only | 4,969 | 6,245 | 5,069 | 6,145 | 11,214 | 1,650,674 |
| QC* | 4,696 | 6,067 | 4,856 | 5,907 | 10,763 | 308,456 |

* Quality control required non-monomorphic bi-allelic SNPs, a genotype call rate of 0.05 for variants and of 0.04 for individuals, no relatives (expected identity-by-descent sharing ≤0.35), in addition to exclusion of PCA-outliers (see table S6).

**Table S6: Excluded PCA-outliers in the SCZD data set.**

| **Nr.** | **Sample-ID** |
| --- | --- |
| 1 | 28304 |
| 2 | 28309 |
| 3 | 28391 |
| 4 | 28449 |
| 5 | 45265 |
| 6 | 45366 |
| 7 | 45438 |
| 8 | 60620 |
| 9 | 71502 |
| 10 | 75693 |
| 11 | 75695 |
| 12 | 107730 |
| 13 | 141934 |
| 14 | 238481 |
| 15 | 313392 |
| 16 | 355922 |
| 17 | 376954 |
| 18 | 377199 |
| 19 | 443738 |
| 20 | 447150 |
| 21 | 447359 |
| 22 | 447545 |
| 23 | 455200 |
| 24 | 481887 |
| 25 | 481890 |
| 26 | 482599 |
| 27 | 482613 |
| 28 | 513409 |
| 29 | 514030 |
| 30 | 573331 |
| 31 | 595814 |
| 32 | 595854 |
| 33 | 654662 |
| 34 | 654900 |

**Table S7: List of previously reported genes in association to AAMD (**[**1**](#_ENREF_1)**).**

| **Chr.** | **Gene** | **Lead variant** |
| --- | --- | --- |
| 1 | *CFH* | rs10922109 (NC_000001.10:g.196704632C>A) |
| 2 | *COL4A3* | rs11884770 (NC_000002.11:g.228086920T>A) |
| 3 | *ADAMTS9/AS2* | \| rs62247658 (NC_000003.11:g.64715155C>T) \|  \| \| --- \| --- \| |
| 3 | *COL8A1* | rs140647181 (NC_000003.11:g.99180668T>C) |
| 4 | *CFI* | rs10033900 (NC_000004.11:g.110659067T>C) |
| 5 | *C9* | rs62358361 (NC_000005.9:g.39327888G>C) |
| 5 | *PRLR/SPEF2* | rs114092250 (NC_000005.9:g.35494448G>A) |
| 6 | *C2/CFB/SKIV2L* | \| rs116503776 (NC_000006.11:g.31930462G>A) \|  \| \| --- \| --- \| |
| 6 | *VEGFA* | rs943080 (NC_000006.11:g.43826627C>T) |
| 7 | *KMT2E/SRPK2* | rs1142 (NC_000007.13:g.104756326C>T) |
| 7 | *PILRB/PILRA* | rs7803454 (NC_000007.13:g.99991548C>T) |
| 8 | *TNRSF10A* | rs79037040 (NC_000008.10:g.23082971G>A) |
| 9 | *TRPM3* | rs71507014 (NC_000009.11:g.73438610_73438611dup) |
| 9 | *MIR6130/RORB* | rs10781182 (NC_000009.11:g.76617720T>G) |
| 9 | *TGFBR1* | rs1626340 (NC_000009.11:g.101923372G>A) |
| 9 | *ABCA1* | rs2740488 (NC_000009.11:g.107661742A>C) |
| 10 | *ARHGAP21* | rs12357257 (NC_000010.10:g.24999593G>A) |
| 10 | *ARMS2/HTRA1* | rs3750846 (NC_000010.10:g.124215565T>C) |
| 12 | *RDH5/CD63* | rs3138141 (NC_000012.11:g.56115778C>A) |
| 12 | *ACAD10* | rs61941274 (NC_000012.11:g.112132610G>A) |
| 13 | *B3GALTL* | rs9564692 (NC_000013.10:g.31821240C>T) |
| 14 | *RAD51B* | rs61985136 (NC_000014.8:g.68769199C>T) |
| 15 | *LIPC* | rs2043085 (NC_000015.9:g.58680954T>C) |
| 16 | *CETP* | rs5817082 (NC_000016.9:g.56997351dup) |
| 16 | *CTRB2* | rs72802342 (NC_000016.9:g.75234872C>A) |
| 17 | *TMEM97/VTN* | rs11080055 (NC_000017.10:g.26649724A>C) |
| 17 | *NPLOC4/TSPAN10* | rs6565597 (NC_000017.10:g.79526821C>T) |
| 19 | *C3* | rs2230199 (NC_000019.9:g.6718387G>C) |
| 19 | *CNN2* | rs67538026 (NC_000019.9:g.1031438C>T) |
| 19 | *APOE* | rs429358 (NC_000019.9:g.45411941T>C) |
| 20 | *MMP9* | rs142450006 (NC_000020.10:g.44614993_44614996TTCT[2]) |
| 20 | *C20orf85* | rs201459901 (NC_000020.10:g.56653725dup) |
| 22 | *SYN3/TIMP3* | rs5754227 (NC_000022.10:g.33105817T>C) |
| 22 | *SLC16A8* | rs8135665 (NC_000022.10:g.38476276C>T) |

**Table S8: List of previously reported rare variants in association with AAMD.**

| **Chr** | **Locus** | **rs-ID** | **Function**** | **MAF** | **p-value** | **Reference*** |
| --- | --- | --- | --- | --- | --- | --- |
| 1 | *CFH* | rs121913059 (NC_000001.10:g.196716375C>T) | 1 | 0.0001 | 9.00×10^-24^ | 1 |
| 1 | *KCNT2-CFH* | rs148553336 (NC_000001.10:g.196613173T>C) | 2 | 0.003 | 9.00×10^-26^ | 1 |
| 1 | *KCNT2* | rs187328863  (NC_000001.10:g.196380158C>T) | 3 | 0.01 | 1.00×10^-68^ | 1 |
| 1 | *CFH* | rs35292876  (NC_000001.10:g.196706642C>T) | 4 | 0.01 | 8.00×10^-37^ | 1 |
| 1 | *CFH* | rs191281603  (NC_000001.10:g.196958651C>G) | 3 | 0.009 | 7.00×10^-07^ | 1 |
| 3 | *LOC105374007* | rs140647181  (NC_000003.11:g.99180668T>C) | 2 | 0.012 | 1.00×10^-11^ | 1 |
| 4 | *CFI* | rs141853578  (NC_000004.11:g.110685820C>T) | 1 | 0.0005 | 6.00×10^-10^ | 1 |
| 5 | *C9* | rs62358361  (NC_000005.9:g.39327888G>C) | 3 | 0.002 | 1.00×10^-14^ | 1 |
| 5 | *PRLR-SPEF2* | rs114092250 (NC_000005.9:g.35494448G>A) | 5 | 0.006 | 2.00×10^-08^ | 1 |
| 6 | *TNXB* | rs12153855 (NC_000006.11:g.32074804T>C) | 3 | 0.011 | 1.00×10^-09^ | 3 |
| 6 | *STK19* | rs2746394  (NC_000006.11:g.31946792G>A) | 3 | 0.008 | 1.00×10^-32^ | 1 |
| 6 | *C2-AS1, C2* | rs9380272  (NC_000006.11:g.31906010G>A) | 3 | 0.012 | 2.00×10^-08^ | 3 |
| 6 | *STK19* | rs181705462  (NC_000006.11:g.31947027G>C) | 3 | 0.01 | 3.00×10^-10^ | 1 |
| 12 | *ACAD10* | rs61941274  (NC_000012.11:g.112132610G>A) | 3 | 0.01 | 1.00×10^-09^ | 1 |
| 15 | *HERC1* | rs74320127 (NC_000015.9:g.64075102T>C) | 3 | 0.05 | 5.00×10^-08^ | 2 |
| 19 | *C3* | rs147859257  (NC_000019.9:g.6718146T>G) | 1 | 0.003 | 3.00×10^-28^ | 1 |

*1 Fritsche et al 2016 ([1](#_ENREF_1)); 2: Yan Q 2018 ([2](#_ENREF_2)); 3: Cipriani V 2012 ([3](#_ENREF_3)).

** Function abbreviations are: 1: missense variant, 2: intergenic variant, 3: intron variant, 4: synonymous variant, and 5: regulatory region variant.

**Table S9: Bins with the locally most significant association signals in AAMD data set, detected by GECS and verified by SKAT.** Each bin is the most significant signal in the block of all overlapping significant bins detected by GECS. These bins are verified by SKAT, adjusted for sex, age, 10 principal components, and common variants in physical proximity, if available (p’-values). For verification with SKAT, we set the threshold at 5×10^-8^.

| **NCT** | **Chr.** | **Bin position (gh19)** | | **Bin size in bp** | **Number of RVs** | **p-value** | **Corrected p-value (p_corr)** | **Wilson lower**  **CI 95%** | **Wilson upper**  **CI 95%** | **OR** | **p'-value** | **Overlapping genes** |
| --- | --- | --- | --- | --- | --- | --- | --- | --- | --- | --- | --- | --- |
| 542 | 4 | 110,685,721 | 110,685,820 | 100 | 5 | 2.15×10^-11^ | 0.002 | 5.49×10^-04^ | 7.27×10^-03^ | 3.43 | 7.03×10^-10^ | *CFI* |
| 1,611 | 1 | 197,814,623 | 197,814,623 | 1 | 1 | 8.08×10^-10^ | 0.002 | 5.49×10^-04^ | 7.27×10^-03^ | 1.453 | 1.20×10^-09^ |  |
| 1,611 | 1 | 197,815,382 | 197,816,763 | 1,382 | 2 | 4.30×10^-17^ | 0.001 | 1.77×10^-04^ | 5.65×10^-03^ | 0.553 | 8.17×10^-15^ |  |
| 1,611 | 1 | 197,829,433 | 197,832,376 | 2,944 | 14 | 1.65×10^-12^ | 0.001 | 1.77×10^-04^ | 5.65×10^-03^ | 0.573 | 2.09×10^-09^ |  |
| 1,611 | 1 | 197,876,319 | 197,880,175 | 3,857 | 10 | 3.69×10^-22^ | 0.001 | 1.77×10^-04^ | 5.65×10^-03^ | 0.733 | 6.93×10^-13^ | *C1orf53* |
| 1,611 | 1 | 197,997,953 | 198,003,207 | 5,255 | 8 | 1.97×10^-11^ | 0.001 | 1.77×10^-04^ | 5.65×10^-03^ | 0.79 | 1.20×10^-08^ |  |
| 1,611 | 1 | 198,094,634 | 198,101,964 | 7,331 | 5 | 1.91×10^-10^ | 0.001 | 1.77×10^-04^ | 5.65×10^-03^ | 0.78 | 3.10×10^-08^ |  |
| 1,611 | 5 | 39,199,134 | 39,199,134 | 1 | 1 | 2.40×10^-10^ | 0.001 | 1.77×10^-04^ | 5.65×10^-03^ | 1.74 | 1.70×10^-10^ | *FYB* |
| 1,611 | 5 | 39,327,884 | 39,327,888 | 5 | 2 | 4.58×10^-12^ | 0.001 | 1.77×10^-04^ | 5.65×10^-03^ | 1.74 | 1.28×10^-11^ | *C9* |
| 1,611 | 5 | 39,331,894 | 39,331,894 | 1 | 1 | 7.49×10^-12^ | 0.001 | 1.77×10^-04^ | 5.65×10^-03^ | 1.73 | 1.47×10^-11^ | *C9* |
| 1,611 | 6 | 31,024,244 | 31,024,244 | 1 | 1 | 1.42×10^-09^ | 0.002 | 5.49×10^-04^ | 7.27×10^-03^ | 0.71 | 3.56×10^-09^ | *HCG22* |
| 1,611 | 6 | 31,421,170 | 31,421,514 | 345 | 4 | 1.14×10^-12^ | 0.001 | 1.77×10^-04^ | 5.65×10^-03^ | 1.31 | 6.37×10^-10^ | *HCP5* |
| 1,611 | 6 | 31,437,566 | 31,437,566 | 1 | 1 | 1.11×10^-09^ | 0.001 | 1.77×10^-04^ | 5.65×10^-03^ | 0.65 | 4.63×10^-10^ | *HCP5* |
| 1,611 | 6 | 31,440,505 | 31,440,641 | 137 | 2 | 2.09×10^-10^ | 0.001 | 1.77×10^-04^ | 5.65×10^-03^ | 0.63 | 6.85×10^-11^ | *HCP5* |
| 1,611 | 6 | 31,444,187 | 31,445,396 | 1,21 | 7 | 4.65×10^-12^ | 0.001 | 1.77×10^-04^ | 5.65×10^-03^ | 0.779 | 1.43×10^-15^ | *HCP5* |
| 1,611 | 6 | 31,458,400 | 31,460,161 | 1,762 | 9 | 2.99×10^-15^ | 0.001 | 1.77×10^-04^ | 5.65×10^-03^ | 1.27 | 2.13×10^-09^ |  |
| 1,611 | 6 | 31,485,262 | 31,487,066 | 1,805 | 7 | 4.81×10^-12^ | 0.001 | 1.77×10^-04^ | 5.65×10^-03^ | 0.72 | 3.48×10^-12^ |  |
| 1,611 | 6 | 31,518,764 | 31,519,389 | 626 | 2 | 2.32×10^-12^ | 0.001 | 1.77×10^-04^ | 5.65×10^-03^ | 0.70 | 5.17×10^-13^ | *NFKBIL1* |
| 1,611 | 6 | 31,522,174 | 31,522,518 | 345 | 2 | 1.80×10^-12^ | 0.001 | 1.77×10^-04^ | 5.65×10^-03^ | 0.65 | 2.03×10^-12^ | *NFKBIL1* |
| 1,611 | 6 | 31,586,761 | 31,592,082 | 5,322 | 23 | 6.95×10^-26^ | 0.001 | 1.77×10^-04^ | 5.65×10^-03^ | 0.72 | 1.14×10^-23^ | *BTNL2* |
| 1,611 | 6 | 31,787,120 | 31,787,539 | 420 | 2 | 5.52×10^-14^ | 0.001 | 1.77×10^-04^ | 5.65×10^-03^ | 0.57 | 9.08×10^-16^ |  |
| 1,611 | 6 | 31,799,353 | 31,800,492 | 1,14 | 4 | 2.60×10^-14^ | 0.001 | 1.77×10^-04^ | 5.65×10^-03^ | 1.30 | 4.80×10^-13^ |  |
| 1,611 | 6 | 31,935,392 | 31,937,762 | 2,371 | 28 | 6.24×10^-76^ | 0.001 | 1.77×10^-04^ | 5.65×10^-03^ | 0.55 | 4.74×10^-81^ | *DXO SKIV2L* |
| 1,611 | 6 | 32,202,920 | 32,202,920 | 1 | 1 | 2.62×10^-15^ | 0.001 | 1.77×10^-04^ | 5.65×10^-03^ | 0.61 | 3.46×10^-15^ |  |
| 1,611 | 6 | 32,216,480 | 32,216,895 | 416 | 3 | 1.03×10^-19^ | 0.001 | 1.77×10^-04^ | 5.65×10^-03^ | 0.69 | 6.96×10^-21^ |  |
| 1,611 | 6 | 32,241,969 | 32,242,178 | 210 | 3 | 1.13×10^-15^ | 0.001 | 1.77×10^-04^ | 5.65×10^-03^ | 0.64 | 1.12×10^-15^ |  |
| 1,611 | 6 | 32,251,835 | 32,252,170 | 336 | 4 | 3.25×10^-13^ | 0.001 | 1.77×10^-04^ | 5.65×10^-03^ | 0.68 | 7.48×10^-13^ |  |
| 1,611 | 6 | 32,270,925 | 32,271,088 | 164 | 2 | 2.12×10^-14^ | 0.001 | 1.77×10^-04^ | 5.65×10^-03^ | 0.59 | 1.47×10^-13^ | *C6orf10* |
| 1,611 | 6 | 32,300,471 | 32,300,824 | 354 | 4 | 1.65×10^-13^ | 0.001 | 1.77×10^-04^ | 5.65×10^-03^ | 0.68 | 3.52×10^-16^ | *C6orf10* |
| 1,611 | 6 | 32,302,546 | 32,302,546 | 1 | 1 | 4.80×10^-14^ | 0.001 | 1.77×10^-04^ | 5.65×10^-03^ | 0.61 | 2.70×10^-14^ | *C6orf10* |
| 1,611 | 6 | 32,304,466 | 32,304,466 | 1 | 1 | 2.45×10^-13^ | 0.001 | 1.77×10^-04^ | 5.65×10^-03^ | 0.62 | 1.59×10^-13^ | *C6orf10* |
| 1,611 | 6 | 32,304,863 | 32,305,102 | 240 | 2 | 3.11×10^-13^ | 0.001 | 1.77×10^-04^ | 5.65×10^-03^ | 0.628 | 2.84×10^-13^ | *C6orf10* |
| 1,611 | 6 | 32,319,173 | 32,319,189 | 17 | 2 | 1.69×10^-16^ | 0.001 | 1.77×10^-04^ | 5.65×10^-03^ | 0.63 | 3.77×10^-16^ | *C6orf10* |
| 1,611 | 6 | 32,340,374 | 32,340,593 | 220 | 2 | 1.61×10^-13^ | 0.001 | 1.77×10^-04^ | 5.65×10^-03^ | 0.68 | 1.52×10^-13^ |  |
| 1,611 | 6 | 32,343,086 | 32,343,588 | 503 | 2 | 1.16×10^-15^ | 0.001 | 1.77×10^-04^ | 5.65×10^-03^ | 0.64 | 1.72×10^-14^ |  |
| 1,611 | 6 | 32,346,903 | 32,346,903 | 1 | 1 | 2.53×10^-13^ | 0.001 | 1.77×10^-04^ | 5.65×10^-03^ | 0.64 | 6.97×10^-13^ |  |
| 1,611 | 6 | 32,351,217 | 32,351,217 | 1 | 1 | 1.76×10^-13^ | 0.001 | 1.77×10^-04^ | 5.65×10^-03^ | 0.64 | 3.30×10^-13^ |  |
| 1,611 | 6 | 32,356,684 | 32,356,684 | 1 | 1 | 1.02×10^-13^ | 0.001 | 1.77×10^-04^ | 5.65×10^-03^ | 0.64 | 1.33×10^-13^ |  |
| 1,611 | 6 | 32,369,601 | 32,369,601 | 1 | 1 | 5.03×10^-14^ | 0.001 | 1.77×10^-04^ | 5.65×10^-03^ | 0.60 | 6.47×10^-15^ | *BTNL2* |
| 1,611 | 6 | 32,395,997 | 32,396,078 | 82 | 2 | 3.74×10^-12^ | 0.001 | 1.77×10^-04^ | 5.65×10^-03^ | 0.66 | 2.84×10^-12^ |  |
| 1,611 | 6 | 32,402,968 | 32,404,276 | 1,309 | 4 | 1.43×10^-11^ | 0.001 | 1.77×10^-04^ | 5.65×10^-03^ | 0.75 | 8.68×10^-14^ |  |
| 1,611 | 10 | 124,156,944 | 124,241,923 | 84,98 | 273 | 1.04×10^-69^ | 0.001 | 1.77×10^-04^ | 5.65×10^-03^ | 0.63 | 2.37×10^-22^ | *ARMS2 HTRA1 MIR3941 PLEKHA1* |
| 1,611 | 19 | 6,718,146 | 6,718,155 | 10 | 2 | 7.87×10^-27^ | 0.001 | 1.77×10^-04^ | 5.65×10^-03^ | 2. 99 | 6.30×10^-28^ | *C3* |
| 2,657 | 1 | 197,920,216 | 197,924,770 | 4,555 | 4 | 5.74×10^-22^ | 0.001 | 1.77×10^-04^ | 5.65×10^-03^ | 0.70 | 1.36×10^-18^ |  |
| 2,657 | 1 | 197,931,243 | 197,939,490 | 8,248 | 7 | 4.32×10^-21^ | 0.001 | 1.77×10^-04^ | 5.65×10^-03^ | 0.75 | 1.86×10^-14^ |  |
| 2,657 | 1 | 198,151,111 | 198,151,688 | 578 | 3 | 5.49×10^-13^ | 0.001 | 1.77×10^-04^ | 5.65×10^-03^ | 0.63 | 7.02×10^-10^ | *NEK7* |
| 2,657 | 1 | 198,210,986 | 198,216,293 | 5,308 | 18 | 8.50×10^-10^ | 0.002 | 5.49×10^-04^ | 7.27×10^-03^ | 0.81 | 1.62×10^-09^ | *NEK7* |
| 2,657 | 6 | 30,075,877 | 30,076,828 | 952 | 10 | 9.81×10^-10^ | 0.002 | 5.49×10^-04^ | 7.27×10-03 | 0.78 | 1.45×10^-11^ | *TRIM31 TRIM31-AS1* |
| 2,657 | 6 | 31,274,584 | 31,274,734 | 151 | 3 | 7.15×10^-14^ | 0.001 | 1.77×10^-04^ | 5.65×10^-03^ | 0.79 | 1.21×10^-11^ |  |
| 2,657 | 6 | 31,322,108 | 31,322,611 | 504 | 6 | 9.80×10^-13^ | 0.001 | 1.77×10^-04^ | 5.65×10^-03^ | 1.28 | 1.62×10^-11^ | *HLA-B* |
| 2,657 | 6 | 31,323,455 | 31,323,745 | 291 | 12 | 3.48×10^-10^ | 0.001 | 1.77×10^-04^ | 5.65×10^-03^ | 1.18 | 2.76×10^-11^ | *HLA-B* |
| 2,657 | 6 | 31,329,415 | 31,331,621 | 2,207 | 16 | 5.77×10^-13^ | 0.001 | 1.77×10^-04^ | 5.65×10^-03^ | 1.23 | 1.57×10^-11^ |  |
| 2,657 | 6 | 31,335,791 | 31,335,983 | 193 | 3 | 1.69×10^-12^ | 0.001 | 1.77×10^-04^ | 5.65×10^-03^ | 1.31 | 2.09×10^-08^ |  |
| 2,657 | 6 | 31,341,327 | 31,342,419 | 1,093 | 9 | 4.10×10^-12^ | 0.001 | 1.77×10^-04^ | 5.65×10^-03^ | 1.22 | 3.09×10^-10^ | *AL671883.1* |
| 2,657 | 6 | 31,346,655 | 31,347,314 | 660 | 7 | 1.84×10^-11^ | 0.001 | 1.77×10^-04^ | 5.65×10^-03^ | 1.25 | 1.47×10^-13^ |  |
| 2,657 | 6 | 31,373,445 | 31,373,957 | 513 | 9 | 5.74×10^-12^ | 0.001 | 1.77×10^-04^ | 5.65×10^-03^ | 1.29 | 1.03×10^-09^ | *HCP5 MICA* |
| 2,657 | 6 | 31,377,662 | 31,378,089 | 428 | 3 | 3.52×10^-13^ | 0.001 | 1.77×10^-04^ | 5.65×10^-03^ | 1.28 | 1.98×10^-09^ | *HCP5 MICA* |
| 2,657 | 6 | 31,378,864 | 31,379,016 | 153 | 5 | 1.76×10^-13^ | 0.001 | 1.77×10^-04^ | 5.65×10^-03^ | 1.30 | 1.07×10^-09^ | *HCP5 MICA* |
| 2,657 | 6 | 31,380,001 | 31,380,601 | 601 | 8 | 2.16×10^-11^ | 0.001 | 1.77×10^-04^ | 5.65×10^-03^ | 1.21 | 1.49×10^-09^ | *HCP5 MICA* |
| 2,657 | 6 | 31,390,874 | 31,391,175 | 302 | 6 | 1.20×10^-14^ | 0.001 | 1.77×10^-04^ | 5.65×10^-03^ | 1.31 | 1.45×10^-11^ | *HCP5* |
| 2,657 | 6 | 31,421,170 | 31,421,547 | 378 | 5 | 4.20×10^-20^ | 0.001 | 1.77×10^-04^ | 5.65×10^-03^ | 1.33 | 1.04×10^-14^ | *HCP5* |
| 2,657 | 6 | 31,444,174 | 31,445,396 | 1,223 | 8 | 2.83×10^-15^ | 0.001 | 1.77×10^-04^ | 5.65×10^-03^ | 0.78 | 1.22×10^-17^ | *HCP5* |
| 2,657 | 6 | 31,473,707 | 31,474,883 | 1,177 | 6 | 1.71×10^-10^ | 0.001 | 1.77×10^-04^ | 5.65×10^-03^ | 1.28 | 2.08×10^-13^ | *MICB* |
| 2,657 | 6 | 31,480,212 | 31,481,149 | 938 | 10 | 4.19×10^-15^ | 0.001 | 1.77×10^-04^ | 5.65×10^-03^ | 1.25 | 1.03×10^-12^ |  |
| 2,657 | 6 | 31,483,892 | 31,485,240 | 1,349 | 6 | 7.72×10^-11^ | 0.001 | 1.77×10^-04^ | 5.65×10^-03^ | 1.28 | 1.70×10^-13^ | *XXbac-BPG16N22.5* |
| 2,657 | 6 | 31,554,382 | 31,554,382 | 1 | 1 | 1.53×10^-10^ | 0.001 | 1.77×10^-04^ | 5.65×10^-03^ | 0.64 | 2.93×10^-11^ | *LST1* |
| 2,657 | 6 | 31,560,910 | 31,561,913 | 1,004 | 5 | 4.03×10^-16^ | 0.001 | 1.77×10^-04^ | 5.65×10^-03^ | 0.73 | 1.55×10^-17^ |  |
| 2,657 | 6 | 31,587,134 | 31,592,082 | 4,949 | 20 | 5.35×10^-35^ | 0.001 | 1.77×10^-04^ | 5.65×10^-03^ | 0.70 | 7.44×10^-37^ | *PRRC2A SNORA38* |
| 2,657 | 6 | 31,600,906 | 31,602,654 | 1,749 | 10 | 7.76×10^-14^ | 0.001 | 1.77×10^-04^ | 5.65×10^-03^ | 0.73 | 3.74×10^-10^ | *PRRC2A* |
| 2,657 | 6 | 31,606,813 | 31,606,977 | 165 | 3 | 5.87×10^-12^ | 0.001 | 1.77×10^-04^ | 5.65×10^-03^ | 0.60 | 1.77×10^-13^ | *BAG6* |
| 2,657 | 6 | 31,607,952 | 31,608,827 | 876 | 6 | 4.39×10^-13^ | 0.001 | 1.77×10^-04^ | 5.65×10^-03^ | 0.71 | 1.92×10^-10^ | *BAG6* |
| 2,657 | 6 | 31,633,482 | 31,633,552 | 71 | 3 | 3.59×10^-16^ | 0.001 | 1.77×10^-04^ | 5.65×10^-03^ | 1.31 | 2.35×10^-14^ | *CSNK2B GPANK1* |
| 2,657 | 6 | 31,647,027 | 31,651,194 | 4,168 | 12 | 3.79×10^-14^ | 0.001 | 1.77×10^-04^ | 5.65×10^-03^ | 1.24 | 1.71×10^-13^ | *LY6G5C* |
| 2,657 | 6 | 31,656,565 | 31,656,565 | 1 | 1 | 6.35×10^-12^ | 0.001 | 1.77×10^-04^ | 5.65×10^-03^ | 0.59 | 1.10×10^-13^ | *ABHD16A* |
| 2,657 | 6 | 31,713,785 | 31,714,220 | 436 | 4 | 1.19×10^-25^ | 0.001 | 1.77×10^-04^ | 5.65×10^-03^ | 1.37 | 6.25×10^-21^ | *MSH5 MSH5-SAPCD1* |
| 2,657 | 6 | 31,729,958 | 31,730,568 | 611 | 3 | 3.30×10^-14^ | 0.001 | 1.77×10^-04^ | 5.65×10^-03^ | 0.73 | 1.12×10^-12^ | *MSH5 MSH5-SAPCD1* |
| 2,657 | 6 | 31,733,224 | 31,737,279 | 4,056 | 26 | 5.69×10^-21^ | 0.001 | 1.77×10^-04^ | 5.65×10^-03^ | 0.76 | 1.15×10^-14^ | *SAPCD1-AS1 VWA7* |
| 2,657 | 6 | 31,761,291 | 31,762,141 | 851 | 3 | 4.02×10^-17^ | 0.001 | 1.77×10^-04^ | 5.65×10^-03^ | 0.56 | 1.06×10^-16^ | *VARS* |
| 2,657 | 6 | 31,770,823 | 31,770,823 | 1 | 1 | 4.81×10^-17^ | 0.001 | 1.77×10^-04^ | 5.65×10^-03^ | 0.62 | 1.10×10^-16^ | *LSM2* |
| 2,657 | 6 | 31,788,479 | 31,792,174 | 3,696 | 9 | 4.35×10^-23^ | 0.001 | 1.77×10^-04^ | 5.65×10^-03^ | 0.73 | 4.43×10^-16^ |  |
| 2,657 | 6 | 31,798,707 | 31,799,223 | 517 | 4 | 5.39×10^-15^ | 0.001 | 1.77×10^-04^ | 5.65×10^-03^ | 0.73 | 1.11×10^-09^ |  |
| 2,657 | 6 | 31,799,353 | 31,800,434 | 1,082 | 5 | 7.47×10^-10^ | 0.002 | 5.49×10^-04^ | 7.27×10-03 | 1.21 | 1.05×10^-09^ |  |
| 2,657 | 6 | 31,807,148 | 31,807,652 | 505 | 7 | 1.22×10^-31^ | 0.001 | 1.77×10^-04^ | 5.65×10^-03^ | 0.64 | 1.01×10^-27^ | *C6orf48* |
| 2,657 | 6 | 31,844,593 | 31,847,180 | 2,588 | 15 | 1.83×10^-49^ | 0.001 | 1.77×10^-04^ | 5.65×10^-03^ | 0.65 | 1.31×10^-56^ | *SLC44A4* |
| 2,657 | 6 | 31,878,006 | 31,878,721 | 716 | 5 | 3.78×10^-80^ | 0.001 | 1.77×10^-04^ | 5.65×10^-03^ | 0.53 | 1.23×10^-70^ | *C2* |
| 2,657 | 6 | 32,006,004 | 32,006,453 | 450 | 4 | 6.80×10^-10^ | 0.001 | 1.77×10^-04^ | 5.65×10^-03^ | 1.36 | 3.32×10^-08^ | *CYP21A2* |
| 2,657 | 6 | 32,017,545 | 32,018,345 | 801 | 3 | 6.61×10^-54^ | 0.001 | 1.77×10^-04^ | 5.65×10^-03^ | 0.56 | 6.61×10^-51^ | *TNXB* |
| 2,657 | 6 | 32,070,602 | 32,070,838 | 237 | 3 | 4.18×10^-48^ | 0.001 | 1.77×10^-04^ | 5.65×10^-03^ | 0.56 | 1.02×10^-42^ | *ATF6B FKBPL* |
| 2,657 | 6 | 32,093,363 | 32,097,421 | 4,059 | 17 | 3.49×10^-54^ | 0.001 | 1.77×10^-04^ | 5.65×10^-03^ | 0.59 | 1.14×10^-56^ | *ATF6B FKBPL* |
| 2,657 | 6 | 32,188,713 | 32,188,853 | 141 | 4 | 2.46×10^-38^ | 0.001 | 1.77×10^-04^ | 5.65×10^-03^ | 0.60 | 1.15×10^-37^ | *NOTCH4* |
| 2,657 | 6 | 32,192,330 | 32,192,560 | 231 | 5 | 5.44×10^-10^ | 0.001 | 1.77×10^-04^ | 5.65×10^-03^ | 0.77 | 5.89×10^-11^ |  |
| 2,657 | 6 | 32,196,249 | 32,196,389 | 141 | 3 | 3.33×10^-15^ | 0.001 | 1.77×10^-04^ | 5.65×10^-03^ | 0.70 | 1.36×10^-13^ |  |
| 2,657 | 6 | 32,198,847 | 32,202,201 | 3,355 | 18 | 2.57×10^-16^ | 0.001 | 1.77×10^-04^ | 5.65×10^-03^ | 1.23 | 1.06×10^-17^ |  |
| 2,657 | 6 | 32,210,714 | 32,211,077 | 364 | 4 | 1.32×10^-13^ | 0.001 | 1.77×10^-04^ | 5.65×10^-03^ | 1.39 | 3.58×10^-16^ |  |
| 2,657 | 6 | 32,231,102 | 32,232,988 | 1,887 | 11 | 2.91×10^-19^ | 0.001 | 1.77×10^-04^ | 5.65×10^-03^ | 0.672 | 4.18×10^-13^ | *XXbac-BPG154L12.4* |
| 2,657 | 6 | 32,256,782 | 32,257,200 | 419 | 4 | 1.33×10^-11^ | 0.001 | 1.77×10^-04^ | 5.65×10^-03^ | 1.34 | 1.01×10^-11^ | *C6orf10* |
| 2,657 | 6 | 32,303,245 | 32,303,474 | 230 | 3 | 3.66×10^-16^ | 0.001 | 1.77×10^-04^ | 5.65×10^-03^ | 1.38 | 1.19×10^-17^ | *C6orf10* |
| 2,657 | 6 | 32,306,922 | 32,308,729 | 1,808 | 8 | 3.62×10^-16^ | 0.001 | 1.77×10^-04^ | 5.65×10^-03^ | 1.28 | 2.98×10^-22^ | *C6orf10* |
| 2,657 | 6 | 32,350,328 | 32,350,622 | 295 | 4 | 7.88×10^-14^ | 0.001 | 1.77×10^-04^ | 5.65×10^-03^ | 1.38 | 4.54×10^-16^ |  |
| 2,657 | 6 | 32,407,537 | 32,407,773 | 237 | 2 | 3.73×10^-12^ | 0.001 | 1.77×10^-04^ | 5.65×10^-03^ | 0.66 | 1.02×10^-12^ | *HLA-DRA* |
| 2,657 | 10 | 124,226,492 | 124,249,185 | 22,694 | 64 | 2.09×10^-84^ | 0.001 | 1.77×10^-04^ | 5.65×10^-03^ | 0.62 | 2.96×10^-30^ | *HTRA1* |
| 2,657 | 19 | 5,827,765 | 5,828,064 | 300 | 2 | 2.49×10^-11^ | 0.001 | 1.77×10^-04^ | 5.65×10^-03^ | 0.74 | 1.04×10^-12^ | *NRTN* |
| 2,657 | 19 | 5,832,268 | 5,832,773 | 506 | 3 | 1.55×10^-10^ | 0.001 | 1.77×10^-04^ | 5.65×10^-03^ | 0.74 | 4.84×10^-12^ | *FUT6* |
| 2,657 | 19 | 5,835,677 | 5,835,677 | 1 | 1 | 8.06×10^-12^ | 0.001 | 1.77×10^-04^ | 5.65×10^-03^ | 0.74 | 1.22×10^-13^ | *FUT6* |
| 2,657 | 22 | 33,074,351 | 33,091,173 | 16,823 | 11 | 1.02×10^-16^ | 0.001 | 1.77×10^-04^ | 5.65×10^-03^ | 0.69 | 5.71×10^-11^ | *SYN3* |
| 2,657 | 22 | 33,097,419 | 33,101,049 | 3,631 | 11 | 1.78×10^-16^ | 0.001 | 1.77×10^-04^ | 5.65×10^-03^ | 0.70 | 1.27×10^-11^ | *SYN3* |

**Table S10: Genes overlapping with significant bins were detected by GECS and verified by SKAT in the AAMD data set. Mapped gene ID refers to availability of the ID in gene ontology.**

| **Chr.** | **Locus name** | **Mapped gene ID** | **Previously reported genes** |
| --- | --- | --- | --- |
| 6 | *ABHD16A* | TRUE | NO |
| 6 | *AL671883.1* | FALSE | NO |
| 10 | *ARMS2* | TRUE | YES |
| 6 | *ATF6B* | TRUE | YES |
| 6 | *BAG6* | TRUE | NO |
| 6 | *BTNL2* | TRUE | NO |
| 6 | *C1orf53* | TRUE | NO |
| 6 | *C2* | TRUE | YES |
| 19 | *C3* | TRUE | YES |
| 6 | *C6orf10* | TRUE | NO |
| 6 | *C6orf48* | TRUE | NO |
| 5 | *C9* | TRUE | YES |
| 4 | *CFI* | TRUE | YES |
| 6 | *CSNK2B* | TRUE | NO |
| 6 | *CYP21A2* | TRUE | NO |
| 6 | *DXO* | TRUE | YES |
| 6 | *FKBPL* | TRUE | NO |
| 19 | *FUT6* | TRUE | YES |
| 5 | *FYB* | TRUE | NO |
| 6 | *GPANK1* | TRUE | NO |
| 6 | *HCG22* | TRUE | NO |
| 6 | *HCP5* | TRUE | NO |
| 6 | *HLA-B* | TRUE | NO |
| 6 | *HLA-DRA* | TRUE | NO |
| 10 | *HTRA1* | TRUE | YES |
| 6 | *LSM2* | TRUE | NO |
| 6 | *LST1* | TRUE | NO |
| 6 | *LY6G5C* | TRUE | NO |
| 6 | *MICA* | TRUE | NO |
| 6 | *MICB* | TRUE | NO |
| 6 | *MIR3941* | FALSE | NO |
| 6 | *MSH5* | TRUE | NO |
| 6 | *MSH5-SAPCD1* | FALSE | NO |
| 1 | *NEK7* | TRUE | NO |
| 6 | *NFKBIL1* | TRUE | NO |
| 6 | *NOTCH4* | TRUE | YES |
| 19 | *NRTN* | TRUE | YES |
| 10 | *PLEKHA1* | TRUE | YES |
| 6 | *PRRC2A* | TRUE | NO |
| 6 | *SKIV2L* | TRUE | YES |
| 6 | *SLC44A4* | TRUE | NO |
| 6 | *SNORA38* | FALSE | NO |
| 22 | *SYN3* | TRUE | YES |
| 6 | *TNXB* | TRUE | YES |
| 6 | *TRIM31* | TRUE | NO |
| 6 | *TRIM31-AS1* | FALSE | NO |
| 6 | *VARS* | TRUE | NO |
| 6 | *VWA7* | TRUE | NO |
| 6 | *XXbac-BPG154L12.4* | FALSE | NO |
| 6 | *XXbac-BPG16N22.5* | FALSE | NO |
| 6 | *XXbac-BPG181B23.4* | FALSE | NO |
| 6 | *XXbac-BPG32J3.20* | FALSE | NO |

**Table S11: A list of gene ontology* results for GECS findings in AAMD data set (significant results in grey).**

| **GO biological process** | **Homo sapiens - REFLIST (20996)** | **upload_1 (44)** | **upload_1 (expected)** | **upload_1 (over/under)** | **upload_1 (fold Enrichment)** | **upload_1**  **(p-value)** |
| --- | --- | --- | --- | --- | --- | --- |
| positive regulation of immune response (GO:0050778) | 778 | 12 | 1.63 | + | 7.36 | 4,40×10^-04^ |
| regulation of immune response (GO:0050776) | 1,056 | 13 | 2.21 | + | 5.87 | 1,45×10^-03^ |
| activation of immune response (GO:0002253) | 560 | 10 | 1.17 | + | 8.52 | 1,86×10^-03^ |
| regulation of immune system process (GO:0002682) | 1,557 | 15 | 3.26 | + | 4.60 | 2,98×10^-03^ |
| positive regulation of immune system process (GO:0002684) | 1,064 | 12 | 2.23 | + | 5.38 | 1,21×10^-02^ |
| regulation of immune effector process (GO:0002697) | 451 | 8 | .95 | + | 8.46 | 3,75×10^-02^ |
| lymphocyte mediated immunity (GO:0002449) | 258 | 6 | 0.54 | + | 11.10 | 1,52×10^-01^ |
| innate immune response (GO:0045087) | 741 | 9 | 1.55 | + | 5.80 | 1,79×10^-01^ |
| adaptive immune response based on somatic recombination of immune receptors built from immunoglobulin superfamily domains (GO:0002460) | 269 | 6 | 0.56 | + | 10.64 | 1,92×10^-01^ |
| negative regulation of immune system process (GO:0002683) | 429 | 7 | 0.90 | + | 7.79 | 2,70×10^-01^ |
| cytolysis (GO:0019835) | 27 | 3 | 0.06 | + | 53.02 | 2,91×10^-01^ |
| immune system process (GO:0002376) | 2,687 | 16 | 5.63 | + | 2.84 | 5,46×10^-01^ |
| positive regulation of lymphocyte mediated immunity (GO:0002708) | 104 | 4 | 0.22 | + | 18.35 | 6,64×10^-0^1 |
| regulation of complement activation (GO:0030449) | 113 | 4 | 0.24 | + | 16.89 | 9,06×10^-01^ |

* Analysis type: PANTHER overrepresentation Test (Released 20181113). Annotation version and release date: GO Ontology, database released 2018-12-01. Analyzed list: upload_1 (Homo sapiens). Reference list: Homo sapiens (all genes in database). Test type: FISHER. Correction: BONFERRONI. Bonferroni count: 8723.

**Table S12: Previously reported rare-variant association signals in AAMD which were detected as significant by GECS (nominal p-values shown, see Methods for more information).**

| **Chr.** | **Locus** | **rs-ID** | **Bin**  **position (hg19)** | | **Number of RV’s** | **p-value** |
| --- | --- | --- | --- | --- | --- | --- |
| 1 | *CFH* | rs121913059 | 196,734,777 | 19,6748,844 | 22 | 4.68×10^-25^ |
| 1 | *KCNT2 - CFH* | rs148553336 | 196,642,858 | 196,644,117 | 11 | 2.94×10^-35^ |
| 1 | *KCNT2* | rs187328863 | 196,383,386 | 96,412,490 | 88 | 8.77×10^-29^ |
| 1 | *CFH* | rs35292876 | 196,730,754 | 196,738,102 | 19 | 8.89×10^-31^ |
| 1 | *CFH* | rs191281603 | 196,958,365 | 196,961,953 | 37 | 1.94×10^-31^ |
| 3 | *LOC105374007* | rs140647181 |  |  |  |  |
| 4 | *CFI* | rs141853578 |  |  |  |  |
| 5 | *C9* | rs62358361 | 39,326,270 | 39,327,888 | 12 | 4.43×10^-10^ |
| 5 | *PRLR - SPEF2* | rs114092250 | 35,494,324 | 35,494,448 | 3 | 3.55×10^-07^ |
| 6 | *TNXB* | rs12153855 | 32,105,360 | 32,109,828 | 14 | 5.86×10^-19^ |
| 6 | *STK19* | rs2746394 | 31,946,792 | 31,947,261 | 5 | 6.12×10^-14^ |
| 6 | *C2-AS1, C2* | rs9380272 | 31,938,120 | 31,938,515 | 7 | 7.72×10^-15^ |
| 6 | *STK19* | rs181705462 | 31,933,248 | 31,980,000 | 125 | 1.99×10^-12^ |
| 12 | *ACAD10* | rs61941274 |  |  |  |  |
| 15 | *HERC1* | rs74320127 |  |  |  |  |
| 19 | *C3* | rs147859257 |  |  |  |  |

T**able S13: Huge bins (number of rare variants ≥ 1,000) that were detected as significant by GECS in the AAMD data set.**

| **NCT** | **Chr.** | **Bin position** | | **Number of rare variants** | **p_value** |
| --- | --- | --- | --- | --- | --- |
| 542 | 1 | 194,548,913 | 197,626,814 | 6,351 | 8.27×10^-10^ |
| 542 | 1 | 196,133,060 | 198,510,045 | 6,168 | 8.61×10^-10^ |
| 542 | 1 | 196,150,322 | 198,524,866 | 6,189 | 6.33×10^-10^ |
| 542 | 10 | 123,863,539 | 124,449,418 | 1,192 | 9.45×10^-10^ |
| 542 | 10 | 123,878,617 | 124,449,418 | 1,185 | 2.88×10^-10^ |
| 542 | 19 | 281,468 | 2,557,330 | 1,082 | 3.91×10^-10^ |
| 542 | 19 | 281,468 | 2,558,709 | 1,083 | 5.27×10^-10^ |
| 1,611 | 1 | 196,311,284 | 197,201,223 | 3,729 | 6.44×10^-10^ |
| 1,611 | 1 | 196,311,578 | 197,201,223 | 3,728 | 6.85×10^-10^ |
| 1,611 | 1 | 196,380,240 | 197,236,152 | 3,730 | 4.37×10^-10^ |
| 1,611 | 10 | 123,988,786 | 124,425,237 | 1,420 | 3.84×10^-10^ |
| 1,611 | 10 | 123,993,600 | 124,435,927 | 1,444 | 1.77×10^-10^ |
| 1,611 | 17 | 78,865,313 | 80,206,871 | 1,785 | 7.43×10^-10^ |
| 1,611 | 17 | 78,982,299 | 80,369,949 | 1,781 | 8.79×10^-10^ |
| 1,611 | 17 | 78,991,475 | 80,372,759 | 1,781 | 7.50×10^-10^ |
| 1,611 | 19 | 1,116,932 | 2,481,236 | 1,205 | 9.09×10^-13^ |
| 1,611 | 19 | 1,116,932 | 2,484,530 | 1,207 | 5.18×10^-13^ |
| 2,657 | 1 | 196,681,376 | 197,026,151 | 1,567 | 3.55×10^-11^ |
| 2,657 | 1 | 196,693,159 | 197,037,552 | 1,546 | 3.39×10^-11^ |
| 2,657 | 1 | 196,693,761 | 197,037,552 | 1,544 | 2.63×10^-11^ |
| 2,657 | 19 | 1,140,069 | 2,301,128 | 1,275 | 3.02×10^-11^ |
| 2,657 | 19 | 1,140,069 | 2,305,170 | 1,280 | 2.88×10^-11^ |
| 2,657 | 19 | 1,140,069 | 2,308,505 | 1,283 | 2.47×10^-11^ |

**Table S14: Counts of significant signals from GECS and SMA in the AAMD data set.**

| **Chr.** | **SMA** | **GECS** | | |
| --- | --- | --- | --- | --- |
|  |  | MAF_T_ = 0.01 | MAF_T_ = 0.03 | MAF_T_ = 0.05 |
| 1 | 2,120 | 170,197 | 143,688 | 58,491 |
| 2 | 0 | 0 | 0 | 0 |
| 3 | 32 | 0 | 15 | 15 |
| 4 | 33 | 6 | 6 | 6 |
| 5 | 3 | 2 | 8 | 8 |
| 6 | 1,536 | 0 | 53,684 | 24,532 |
| 7 | 1 | 0 | 0 | 0 |
| 8 | 0 | 0 | 0 | 0 |
| 9 | 45 | 0 | 0 | 2 |
| 10 | 1,093 | 6,960 | 72,537 | 38,329 |
| 11 | 2 | 0 | 0 | 925 |
| 12 | 9 | 0 | 0 | 0 |
| 13 | 3 | 0 | 0 | 0 |
| 14 | 0 | 0 | 0 | 0 |
| 15 | 19 | 0 | 0 | 0 |
| 16 | 62 | 0 | 0 | 0 |
| 17 | 4 | 1 | 19,702 | 1,003 |
| 18 | 0 | 0 | 0 | 0 |
| 19 | 514 | 9,582 | 43,154 | 59,800 |
| 20 | 49 | 0 | 2 | 20 |
| 21 | 0 | 0 | 456 | 0 |
| 22 | 44 | 0 | 0 | 354 |
| **SUM** | **5,590** | **186,748** | **333,252** | **183,485** |

**Table S15: Bins with the locally most significant association signals in SCZD data set, detected by GECS and verified by SKAT.** Each bin is the most significant signal in the block of all overlapping significant bins detected by GECS. These bins are verified by SKAT, adjusted for sex, age, 10 principal components, and common variants in physical proximity, if available (p’-values). For verification with SKAT, we set the threshold at 2×10^-6^.

| **NCT** | **Chr.** | **Bin position** | | **Bin size** | **Number of rare variants** | **p-value** | **Corrected p-value (p_corr)** | **Wilson lower**  **CI 95%** | **Wilson upper**  **CI 95%** | **OR** | **p'-value** | **Overlapping or close genes (+/- 10kb)** |
| --- | --- | --- | --- | --- | --- | --- | --- | --- | --- | --- | --- | --- |
| 214 | 2 | 38,903,107 | 38,956,836 | 53,73 | 3 | 2.61×10^-10^ | 0.001 | 1.77×10^-04^ | 5.65×10^-03^ | 0.20 | 2.97×10^-09^ | *GALM* |
| 214 | 2 | 61,719,303 | 61,719,303 | 1 | 1 | 1.07×10^-11^ | 0.001 | 1.77×10^-04^ | 5.65×10^-03^ | 0.32 | 8.05×10^-13^ | *XPO1* |
| 214 | 3 | 187,444,543 | 187,444,543 | 1 | 1 | 1.07×10^-12^ | 0.001 | 1.77×10^-04^ | 5.65×10^-03^ | 0.18 | 1.11×10^-14^ | *BCL6* |
| 214 | 4 | 47,887,513 | 47,887,513 | 1 | 1 | 1.26×10^-08^ | 0.017 | 1.77×10^-04^ | 2.71×10^-02^ | 0.39 | 2.42×10^-09^ | *NFXL1* |
| 214 | 5 | 178,392,653 | 178,392,792 | 140 | 2 | 1.02×10^-08^ | 0.014 | 8.37×10^-03^ | 2.34×10^-02^ | 0.19 | 3.63×10^-09^ | *ZNF454* |
| 214 | 6 | 150,290,454 | 150,291,207 | 754 | 2 | 2.70×10^-13^ | 0.001 | 1.77×10^-04^ | 5.65×10^-03^ | 0.27 | 1.90×10^-14^ | *ULBP1* |
| 214 | 7 | 129,680,877 | 129,680,877 | 1 | 1 | 1.38×10^-08^ | 0.019 | 1.22×10^-02^ | 2.95×10^-02^ | 0.13 | 1.66×10^-09^ | *ZC3HC1* |
| 214 | 11 | 57,100,475 | 57,100,642 | 168 | 2 | 1.56×10^-12^ | 0.001 | 1.77×10^-04^ | 5.65×10^-03^ | 0.04 | 1.65×10^-12^ | *SSRP1* |
| 214 | 11 | 82,642,868 | 82,642,868 | 1 | 1 | 2.23×10^-09^ | 0.003 | 1.02×10^-03^ | 8.79×10^-03^ | 1.01 | 1.54×10^-10^ | *PRCP* |
| 214 | 12 | 53,455,228 | 53,455,228 | 1 | 1 | 3.63×10^-10^ | 0.001 | 1.77×10^-04^ | 5.65×10^-03^ | 0.31 | 1.35×10^-10^ | *TENC1* |
| 214 | 12 | 57,398,026 | 57,398,026 | 1 | 1 | 1.40×10^-11^ | 0.001 | 1.77×10^-04^ | 5.65×10^-03^ | 0.20 | 2.17×10^-13^ | *ZBTB39* |
| 214 | 13 | 31,897,996 | 31,897,996 | 1 | 1 | 9.72×10^-10^ | 0.002 | 5.49×10^-04^ | 7.27×10^-03^ | 0.10 | 5.47×10^-12^ | *B3GALTL* |
| 214 | 14 | 60,611,664 | 60,611,664 | 1 | 1 | 1.41×10^-08^ | 0.021 | 1.38×10^-02^ | 3.19×10^-02^ | 0.17 | 6.09×10^-10^ | *DHRS7 PCNXL4* |
| 214 | 14 | 89,078,090 | 89,088,986 | 10,897 | 2 | 1.14×10^-09^ | 0.002 | 5.49×10^-04^ | 7.27×10^-03^ | 0.14 | 9.19×10^-11^ | *EML5* |
| 214 | 17 | 49,239,143 | 49,239,143 | 1 | 1 | 2.72×10^-09^ | 0.003 | 1.02×10^-03^ | 8.79×10^-03^ | 0.13 | 5.58×10^-11^ | *NME1 NME1-NME2 NME2* |
| 214 | 22 | 17,687,954 | 17,688,129 | 176 | 2 | 3.79×10^-13^ | 0.001 | 1.77×10^-04^ | 5.65×10^-03^ | 0.27 | 6.77×10^-14^ | *CECR1* |
| 636 | 1 | 24,447,832 | 24,447,843 | 12 | 2 | 1.28×10^-14^ | 0.001 | 1.77×10^-04^ | 5.65×10^-03^ | 0.38 | 5.86×10^-16^ | *IL22RA1* |
| 636 | 3 | 138,402,579 | 138,402,579 | 1 | 1 | 1.42×10^-21^ | 0.001 | 1.77×10^-04^ | 5.65×10^-03^ | 0.30 | 8.36×10^-23^ | *PIK3CB* |
| 636 | 4 | 103,720,097 | 103,806,388 | 86,292 | 6 | 3.19×10^-17^ | 0.001 | 1.77×10^-04^ | 5.65×10^-03^ | 0.35 | 4.07×10^-18^ | *CISD2 RNU7-151P RP11-10L12.4 SLC9B1 snoU13 UBE2D3* |
| 636 | 5 | 32,415,110 | 32,415,216 | 107 | 2 | 2.12×10^-17^ | 0.001 | 1.77×10^-04^ | 5.65×10^-03^ | 0.24 | 3.52×10^-18^ | *ZFR* |
| 636 | 5 | 54,591,272 | 54,591,272 | 1 | 1 | 3.95×10^-12^ | 0.001 | 1.77×10^-04^ | 5.65×10^-03^ | 0.38 | 1.20×10^-14^ | *DHX29* |
| 636 | 7 | 128,413,777 | 128,413,777 | 1 | 1 | 1.96×10^-15^ | 0.001 | 1.77×10^-04^ | 5.65×10^-03^ | 0.42 | 7.85×10^-17^ | *OPN1SW* |
| 636 | 12 | 53,069,392 | 53,069,392 | 1 | 1 | 1.19×10^-09^ | 0.003 | 1.02×10^-03^ | 8.79×10^-03^ | 0.58 | 8.07×10^-10^ | *KRT1* |
| 636 | 14 | 67,848,325 | 67,848,325 | 1 | 1 | 3.67×10^-13^ | 0.001 | 1.77×10^-04^ | 5.65×10^-03^ | 0.35 | 4.27×10^-12^ | *EIF2S1* |
| 636 | 15 | 73,044,829 | 73,044,833 | 5 | 2 | 1.17×10^-19^ | 0.001 | 1.77×10^-04^ | 5.65×10^-03^ | 0.43 | 4.30×10^-20^ | *ADPGK* |
| 636 | 19 | 8,999,386 | 9,028,410 | 29,025 | 62 | 2.59×10^-09^ | 0.004 | 1.56×10^-03^ | 1.02×10^-02^ | 1.29 | 3.10×10^-10^ | *MUC16* |
| 1,049 | 1 | 202,724,559 | 202,729,678 | 5,12 | 3 | 3.06×10^-13^ | 0.001 | 1.77×10^-04^ | 5.65×10^-03^ | 0.42 | 3.77×10^-13^ | *KDM5B* |
| 1,049 | 9 | 33,796,672 | 33,798,630 | 1,959 | 20 | 5.07×10^-10^ | 0.003 | 1.02×10^-03^ | 8.79×10^-03^ | 1.37 | 3.89×10^-11^ | *PRSS3* |

**Table S16: Genes overlapping with significant bins were detected by GECS and verified by SKAT in the SCZD data set. Unmapped gene ID refers to availability of the ID in gene ontology.**

| **Chr.** | **Locus name** | **Unmapped gene ID** | **Previously reported**  **(Purcell et al 2014, Genovese et al 2016)** |
| --- | --- | --- | --- |
| 1 | *IL22RA1* | NO | YES |
| 1 | *KDM5B* | NO | YES |
| 1 | *KLHL12* | NO | YES |
| 1 | *RABIF* | NO | NO |
| 1 | *HNRNPA1P59* | YES | NO |
| 1 | *PCAT6* | YES | NO |
| 1 | *RP11-480I12.10* | YES | NO |
| 1 | *RP11-480I12.2* | YES | NO |
| 1 | *RP11-480I12.4* | YES | NO |
| 1 | *RP11-480I12.5* | YES | NO |
| 1 | *RP11-480I12.7* | YES | NO |
| 1 | *RP11-480I12.9* | YES | NO |
| 1 | *SLC25A39P1* | YES | NO |
| 1 | *Y_RNA* | YES | NO |
| 2 | *XPO1* | NO | NO |
| 2 | *GALM* | NO | YES |
| 3 | *BCL6* | NO | YES |
| 3 | *PIK3CB* | NO | YES |
| 3 | *RP11-211G3* | YES | NO |
| 4 | *NFXL1* | NO | YES |
| 4 | *CISD2* | NO | No |
| 4 | *SLC9B1* | NO | YES |
| 4 | *UBE2D3* | NO | No |
| 4 | *RNU7-151P* | YES | NO |
| 4 | *RP11-10L12* | YES | NO |
| 4 | *snoU13* | YES | NO |
| 5 | *ZNF454* | NO | YES |
| 5 | *DHX29* | NO | YES |
| 5 | *ZFR* | NO | NO |
| 6 | *ULBP1* | NO | NO |
| 7 | *ZC3HC1* | NO | YES |
| 7 | *OPN1SW* | NO | YES |
| 9 | *PRSS3* | NO | NO |
| 9 | *RP11-133O22.6* | YES | NO |
| 11 | *SSRP1* | NO | NO |
| 11 | *PRCP* | NO | YES |
| 11 | *C11orf82* | NO | YES |
| 12 | *ZBTB39* | NO | YES |
| 12 | *KRT1* | NO | YES |
| 12 | *TENC1* | YES | YES |
| 13 | *B3GALTL* | NO | YES |
| 14 | *DHRS7* | NO | NO |
| 14 | *EML5* | NO | YES |
| 14 | *ZC3H14* | NO | NO |
| 14 | *PCNXL4* | NO | YES |
| 14 | *EIF2S1* | NO | NO |
| 15 | *ADPGK* | NO | NO |
| 17 | *NME1* | NO | YES |
| 17 | *NME2* | NO | NO |
| 19 | *MUC16* | NO | YES |
| 22 | *CECR1* | NO | YES |

**Table S17: Largest bins (number of rare variants ≥ 50) that were detected as significant by GECS in the SCZD data set.**

| **NCT** | **Chr.** | **Bin position** | | **Number of rare variants** | **p-value** |
| --- | --- | --- | --- | --- | --- |
| 214 | 1 | 16,525,790 | 16,918,465 | 77 | 6.54×10^-09^ |
| 214 | 1 | 16,528,955 | 16,918,473 | 77 | 2.93×10^-09^ |
| 214 | 1 | 16,528,955 | 17,030,399 | 80 | 3.33×10^-09^ |
| 214 | 1 | 16,531,261 | 16,918,523 | 77 | 3.92×10^-09^ |
| 636 | 7 | 128,049,496 | 128,471,084 | 56 | 6.06×10^-09^ |
| 636 | 7 | 128,049,590 | 128,471,084 | 54 | 4.70×10^-09^ |
| 636 | 16 | 89,168,971 | 89,347,730 | 89 | 1.92×10^-08^ |
| 636 | 19 | 8,994,484 | 9,026,247 | 66 | 1.03×10^-08^ |
| 636 | 19 | 8,997,119 | 9,026,247 | 65 | 1.25×10^-08^ |
| 636 | 19 | 8,997,472 | 9,026,247 | 64 | 8.74×10^-09^ |
| 636 | 19 | 8,998,663 | 9,026,247 | 63 | 7.79×10^-09^ |

**Table S18: Performance of GECS in simulated data sets under the null model.**

| **Method** | $\boldsymbol{N}_{\boldsymbol{T}}^{\boldsymbol{C}}$ | **MAF_T_** | **Number of variants (millions)** | **Sample size** | **Average time* (h)** | **Average memory in gb** | **Average number of all bins* (millions)** | **Average number of distinct bins* (millions)** | **Average reduction rate** |
| --- | --- | --- | --- | --- | --- | --- | --- | --- | --- |
| **GECS** | 19 | 0.01 | 3 | 1,000 | 6 | 3 | 295,000 | 276 | 99.91% |
|  | 59 | 0.03 | 5 | 1,000 | 6 | 4 | 795,000 | 285 | 99.96% |
|  | 97 | 0.05 | 6 | 1,000 | 6 | 4 | 1,064,000 | 259 | 99.98% |
| **SMA** |  |  | 12 | 1,000 | 0.2 | 7 |  |  |  |
| **GECS** | 99 | 0.01 | 4 | 5,000 | 42 | 11 | 386,000 | 730 | 99.81% |
|  | 295 | 0.03 | 6 | 5,000 | 38 | 13 | 902,000 | 667 | 99.93% |
|  | 487 | 0.05 | 6 | 5,000 | 34 | 13 | 1,186,000 | 599 | 99.95% |
| **SMA** |  |  | 12 | 5,000 | 3 | 19 |  |  |  |
| **GECS** | 199 | 0.01 | 4 | 10,000 | 129 | 20 | 411,000 | 1,083 | 99.74% |
|  | 591 | 0.03 | 6 | 10,000 | 110 | 22 | 1,001,000 | 931 | 99.91% |
|  | 975 | 0.05 | 7 | 10,000 | 98 | 24 | 1,205,000 | 890 | 99.93% |
| **SMA** |  |  | 12 | 10,000 | 4 | 34 |  |  |  |
| **GECS** | 398 | 0.01 | 4 | 20,000 | 452 | 40 | 413,000 | 1,504 | 99.64% |
|  | 1,181 | 0.03 | 6 | 20,000 | 380 | 34 | 1,070,000 | 1,360 | 99.86% |
|  | 1,950 | 0.05 | 7 | 20,000 | 340 | 29 | 1,213,000 | 1,230 | 99.88% |
| **SMA** |  |  | 13 | 20,000 | 12 | 63 |  |  |  |
| **GECS**** | 398 | 0.01 | 0.05-0.3 | 20,000 | 6-100 | >1-3 | 1,339-57,010 | 20-130 | 98.44%-99.77% |
|  | 1,181 | 0.03 | 0.07-0.5 | 20,000 | 5-85 | >1-3 | 3,468-147,655 | 50-325 | 98.67%-99.91% |
|  | 1,950 | 0.05 | 0.09-0.6 | 20,000 | 4-75 | >1-3 | 3,953-168,327 | 56-360 | 98.73%-99.93% |

* Average across 1,000 simulations.

** GECS was performed in parallel for each chromosome independently; min and max values among all chromosomes were considered.

**Table S19: Performance of GECS in the imputed whole-genome AAMD data set and in the whole-exome SCZD data set.**

|  | **Method** | $\boldsymbol{N}_{\boldsymbol{T}}^{\boldsymbol{C}}$ | **MAF_T_** | **Number of variants on chromosomes** | **Sample size** | **Time (h)** | **Memory in gb** | **Number of all bins (millions)** | **Number of distinct bins (millions)** |
| --- | --- | --- | --- | --- | --- | --- | --- | --- | --- |
| **AAMD** | **GECS** | 542 | 0.01 | 34-267k | 27,259 | 3 | >1-4 | 600-38,000 | 5-35 |
|  |  | 1,611 | 0.03 | 54-406k |  | 4 | >1-4 | 1,000-82,000 | 6-40 |
|  |  | 2,657 | 0.05 | 64-470k |  | 3 | >1-5 | 2,000-111,000 | 5-36 |
|  | **SMA** |  |  | 927k |  | 14 | 72 |  |  |
| **SCZD** | **GECS** | 214 | 0.01 | 219k | 10,763 | 14 | >1 | 1,300 | 116 |
|  |  | 636 | 0.03 | 241k |  | 10 | 1 | 1,600 | 65 |
|  |  | 1,049 | 0.05 | 251k |  | 8 | 1 | 1,700 | 49 |
|  | **SMA** |  |  | 308k |  | 6 | 2 |  |  |

**Table S20: Plots visualizing the results of the power study for different parameters.**

|  | **Figure** | | | |
| --- | --- | --- | --- | --- |
|  | **Rare diseases, K=0.01** | | **Common diseases, K=0.1** | |
| **Sample size** | **PNV=0.1** | **PNV=0.3** | **PNV=0.1** | **PNV=0.3** |
| 1,000 | Figure S9 | Figure 2 | Figure S11 | Figure S6 |
| 10,000 | Figure S10 | Figure 3 | Figure S12 | Figure S7 |
| 20,000 | - | Figure S5 | - | Figure S8 |

## Figures:

**Figure S1: Combined carrier matrix B and inductive computation of bins.** The arrays $v_{1}, v_{2}, \ldots, v_{n}$ are binary, each of length *N*, where *N* denotes number of samples and the elements $v_{i,l}$ indicate the carrier status of the *l*-th individual (1=carrier, 0=non-carrier) at the *i-th* variant.


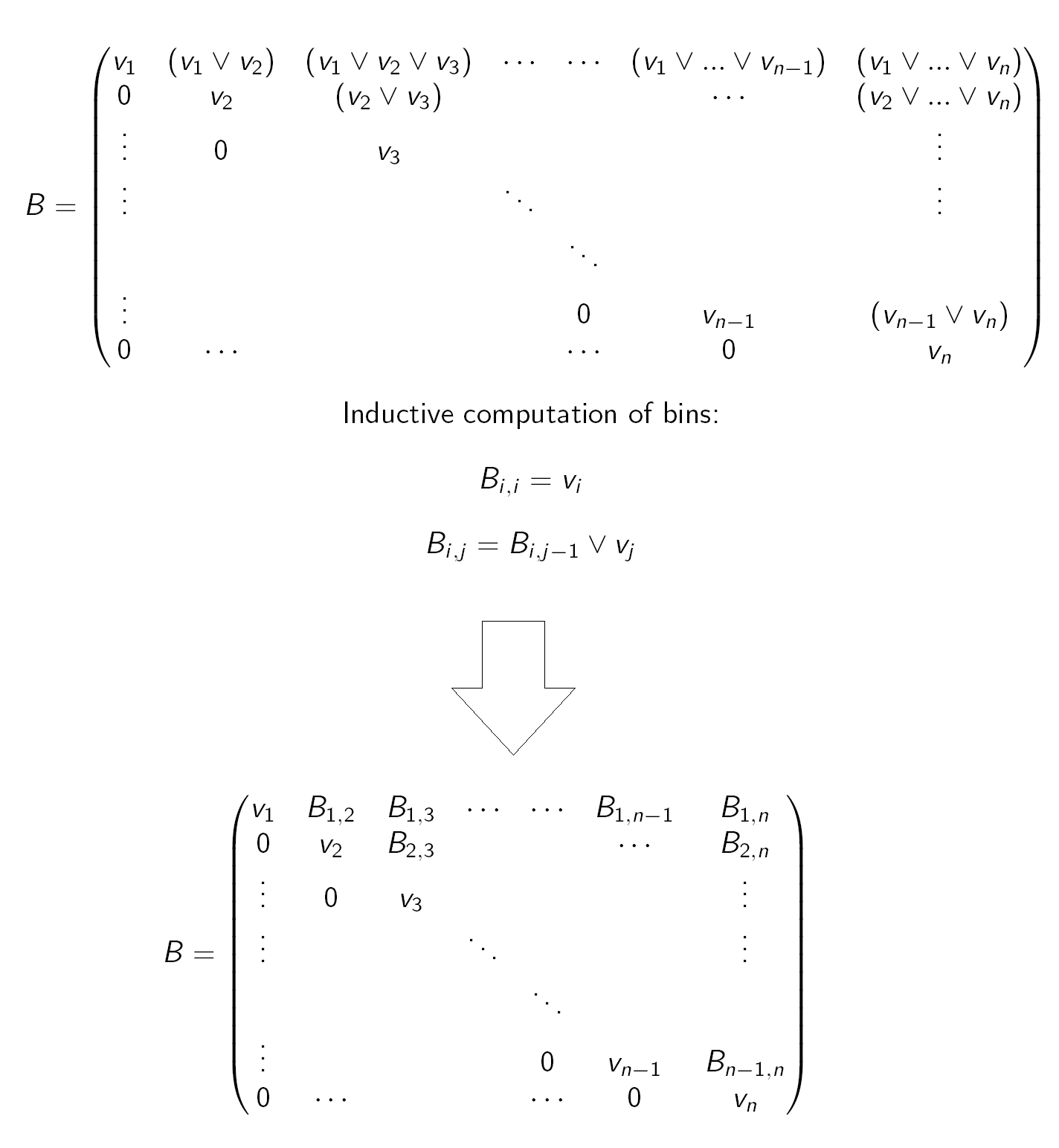


**Figure S2: In-silico post-GECS analysis**

**^1)^ As the strongest signal, we define bins with the lowest SKAT-p*-value that overlap with the most significant bin found by GECS.**

**Figure S3: P-value densities obtained from GECS in 1,000 simulated studies with 1,000 samples each, compared to corresponding results from single-marker analyses (SMA).** MAF_T_: minor allele frequency threshold.

-log_10_(p-value)


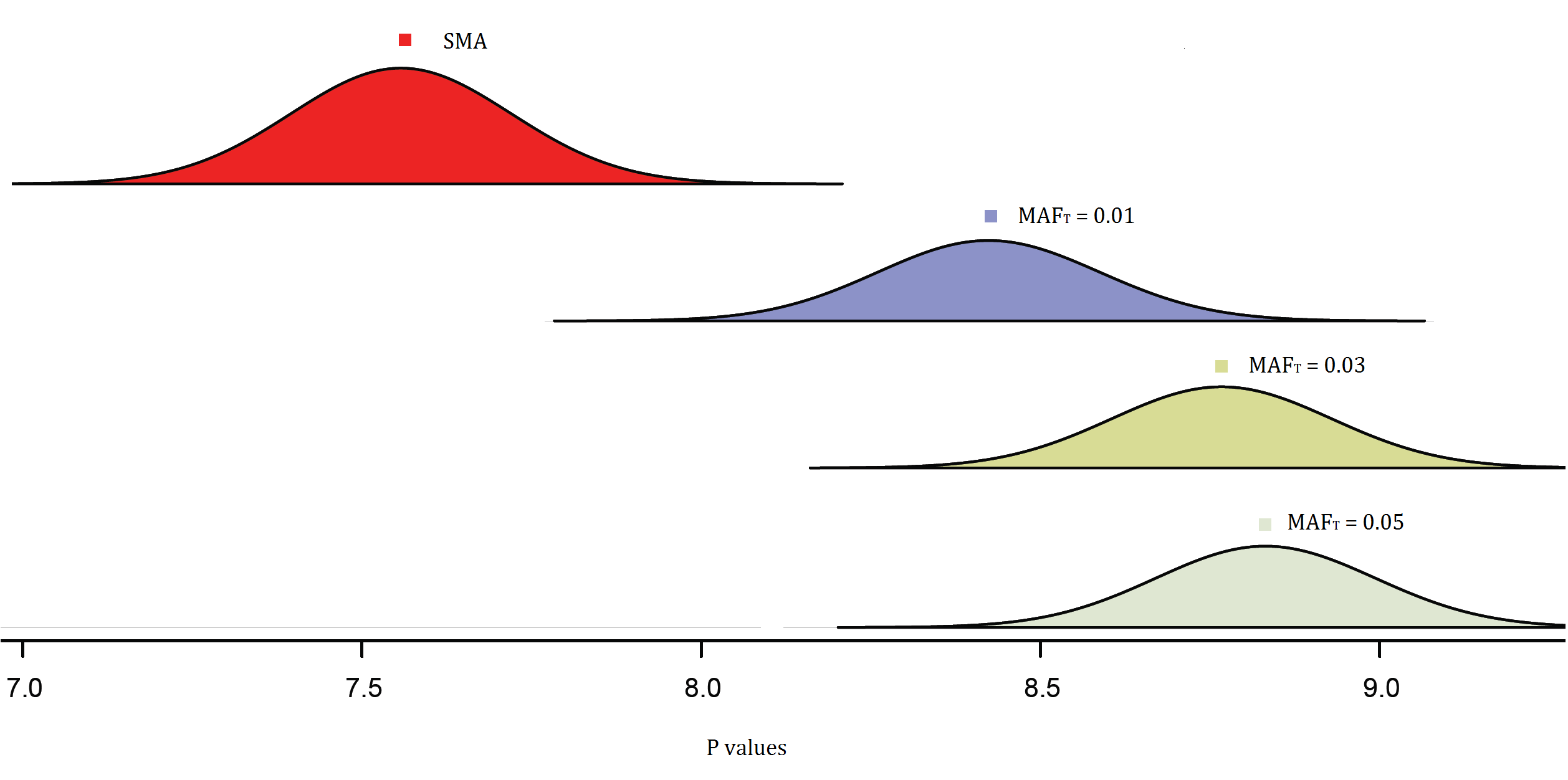


Log_10(P value)

**Figure S4: P-value densities obtained from GECS in 1000 simulated studies with 5,000, 10,000, and 20,000 samples each, compared to corresponding results from single-marker analyses (SMA).** Distributions always shifted to the right with increasing sample sizes.


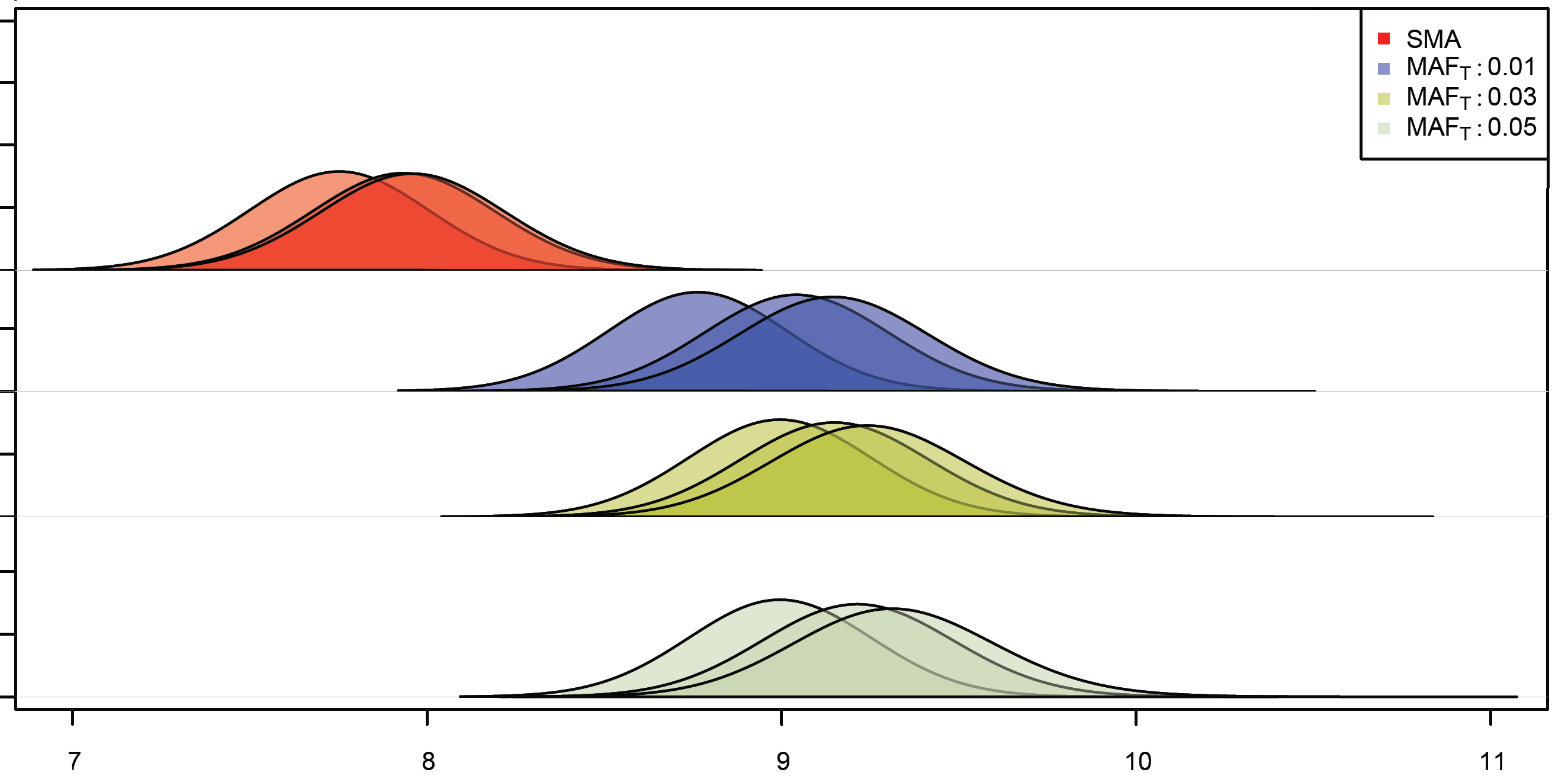


-log_10_(p-value)

**Figure S5: Comparative power analysis for a rare disease (prevalence K=0.01) and large sample size (N=20,000).** Results are given for studies with proportion of neutral rare variants (PNV) = 0.3, different simulated window sizes (x-axis), and different proportions of detrimental rare variants (PDV) (y-axis). Black lines: GECS; red lines: SMA. In each grid cell, the power is presented on the y-axis and OR intervals on the x-axis. For an overview see table S20.

**
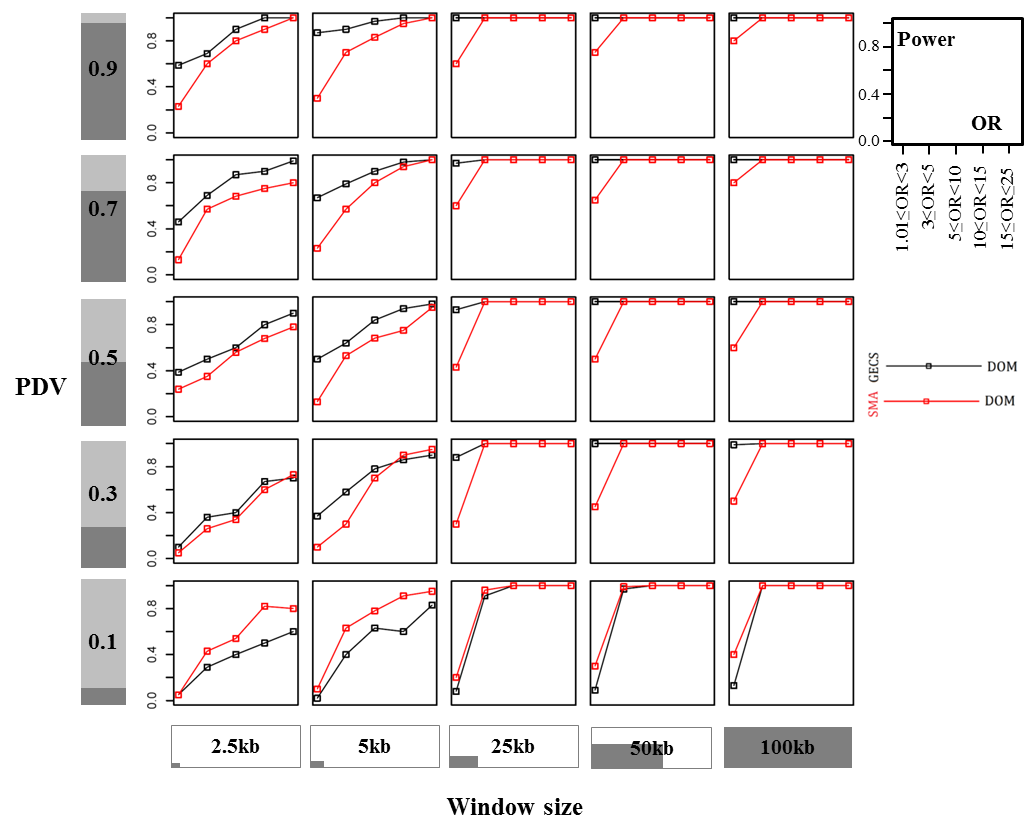
**

**Figure S6: Comparative power analysis for a common disease (prevalence K=0.1) and small sample size (N=1,000).** Results are given for studies with proportion of neutral rare variants (PNV) = 0.3, different simulated window sizes (x-axis), and different proportions of detrimental rare variants (PDV) (y-axis). Black lines: GECS; red lines: SMA. In each grid cell, the power is presented on the y-axis and OR intervals on the x-axis. For an overview see table S20.

**
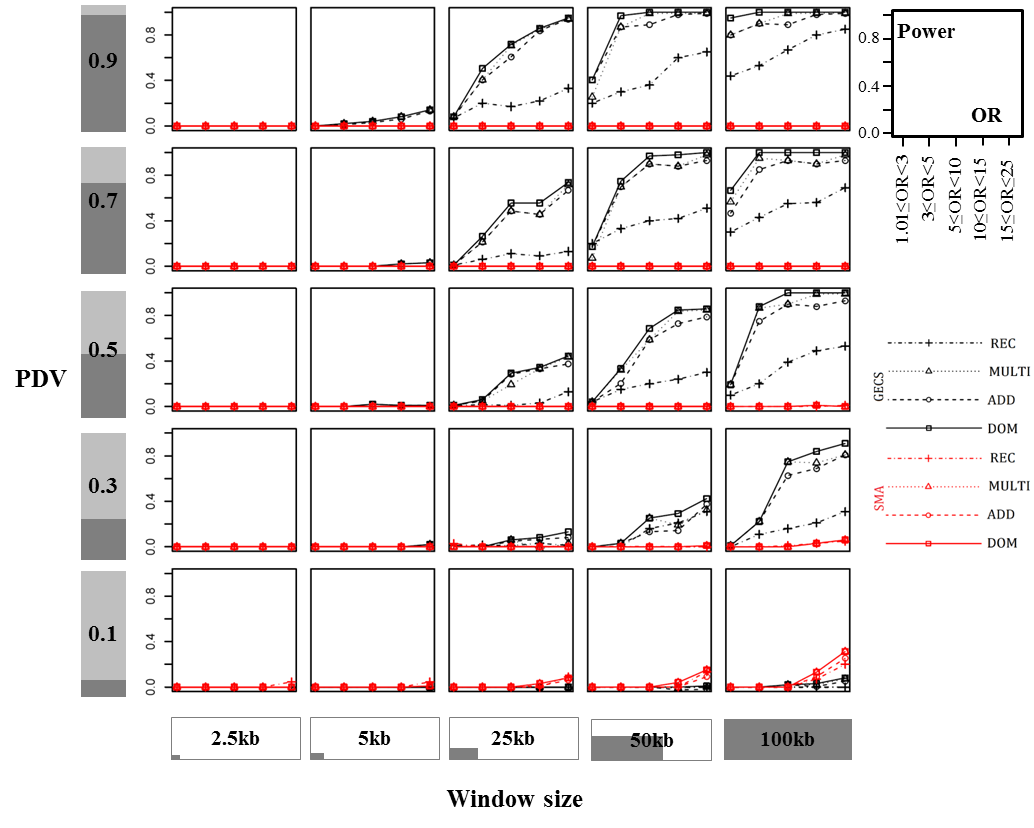
**

**Figure S7: Comparative power analysis for a common disease (prevalence K=0.1) and moderate sample size (N=10,000).** Results are given for studies with proportion of neutral rare variants (PNV) = 0.3, different simulated window sizes (x-axis), and different proportions of detrimental rare variants (PDV) (y-axis). Black lines: GECS; red lines: SMA. In each grid cell, the power is presented on the y-axis and OR intervals on the x-axis. For an overview see table S20.

**
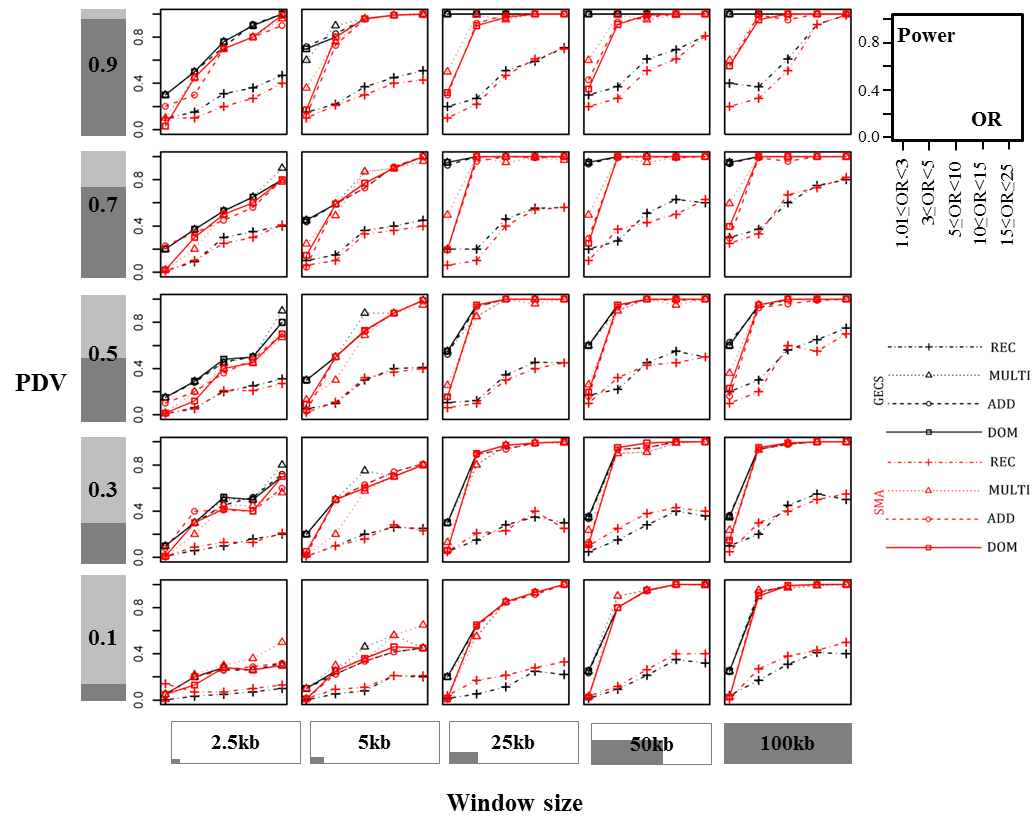
**

**Figure S8: Comparative power analysis for a common disease (prevalence K=0.1) and large sample size (N=20,000).** Results are given for studies with proportion of neutral rare variants (PNV) = 0.3, different simulated window sizes (x-axis), and different proportions of detrimental rare variants (PDV) (y-axis). Black lines: GECS; red lines: SMA. In each grid cell, the power is presented on the y-axis and OR intervals on the x-axis. For an overview see table S20.
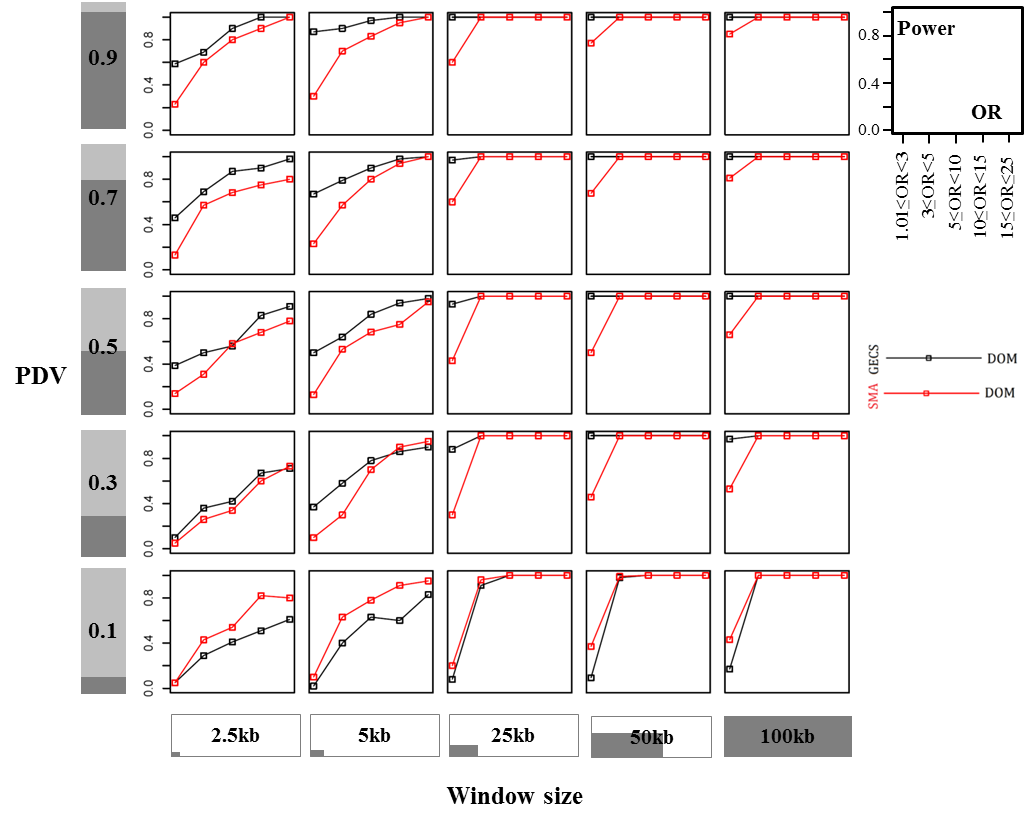


**Figure S9: Comparative power analysis for a rare disease (prevalence K=0.01) and small sample size (N=1,000).** Results are given for studies with proportion of neutral rare variants (PNV) = 0.1, different simulated window sizes (x-axis), and different proportions of detrimental rare variants (PDV) (y-axis). Black lines: GECS; red lines: SMA. In each grid cell, the power is presented on the y-axis and OR intervals on the x-axis. For an overview see table S20.

**
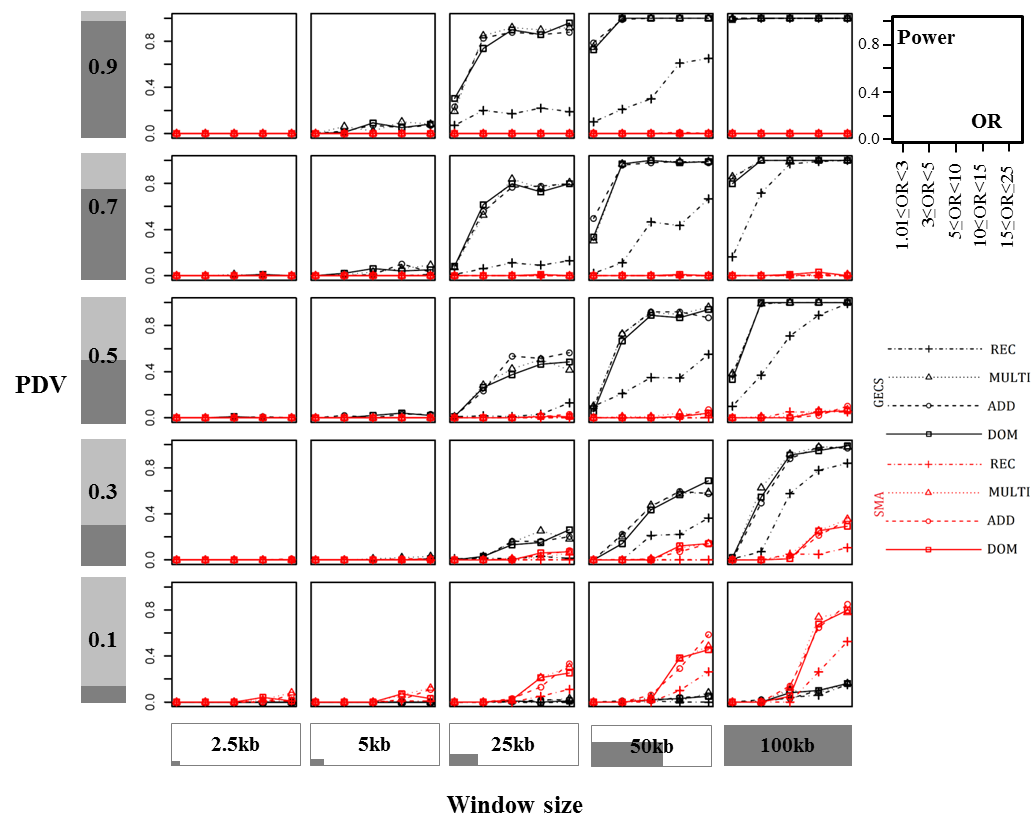
**

**Figure S10:** **Comparative power analysis for a rare disease (prevalence K=0.01) and moderate sample size (N=10,000).** Results are given for studies with proportion of neutral rare variants (PNV) = 0.1, different simulated window sizes (x-axis), and different proportions of detrimental rare variants (PDV) (y-axis). Black lines: GECS; red lines: SMA. In each grid cell, the power is presented on the y-axis and OR intervals on the x-axis. For an overview see table S20.

**
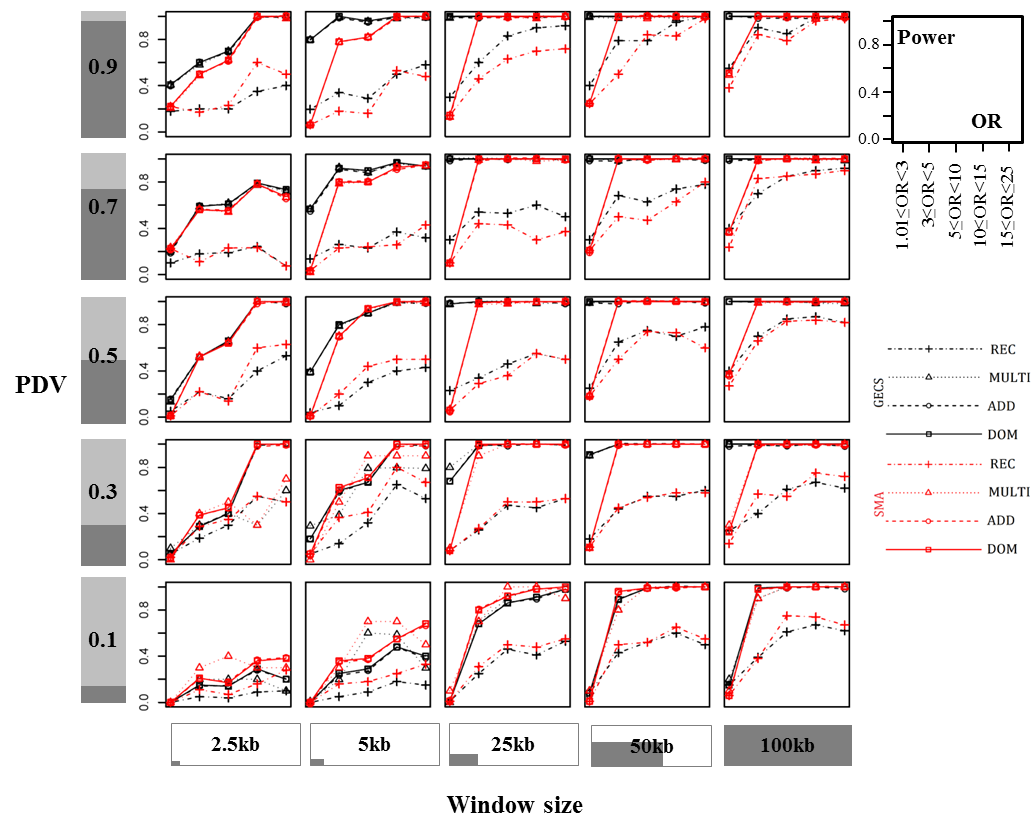
**

**Figure S11: Comparative power analysis for a common disease (prevalence K=0.1) and small sample size (N=1,000).** Results are given for studies with proportion of neutral rare variants (PNV) = 0.1, different simulated window sizes (x-axis), and different proportions of detrimental rare variants (PDV) (y-axis). Black lines: GECS; red lines: SMA. In each grid cell, the power is presented on the y-axis and OR intervals on the x-axis. For an overview see table S20.

**
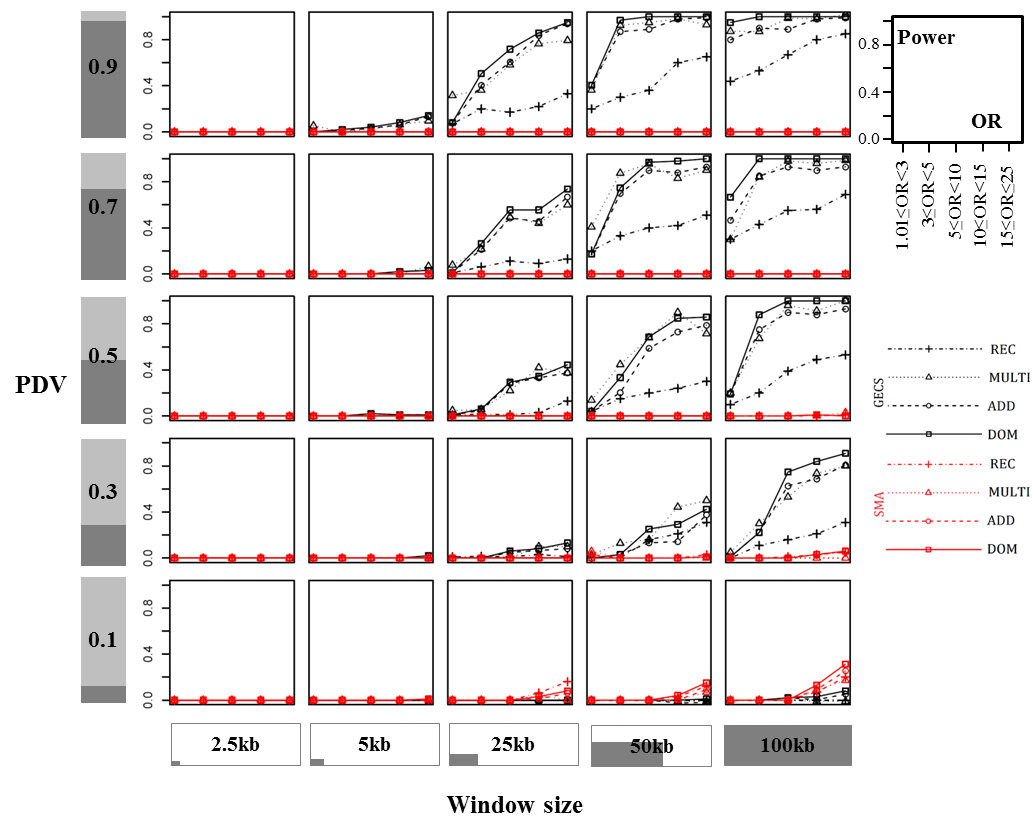
**

**Figure S12: Comparative power analysis for a common disease (prevalence K=0.1) and moderate sample size (N=10,000).** Results are given for studies with proportion of neutral rare variants (PNV) = 0.1, different simulated window sizes (x-axis), and different proportions of detrimental rare variants (PDV) (y-axis). Black lines: GECS; red lines: SMA. In each grid cell, the power is presented on the y-axis and OR intervals on the x-axis. For an overview see table S20.

**
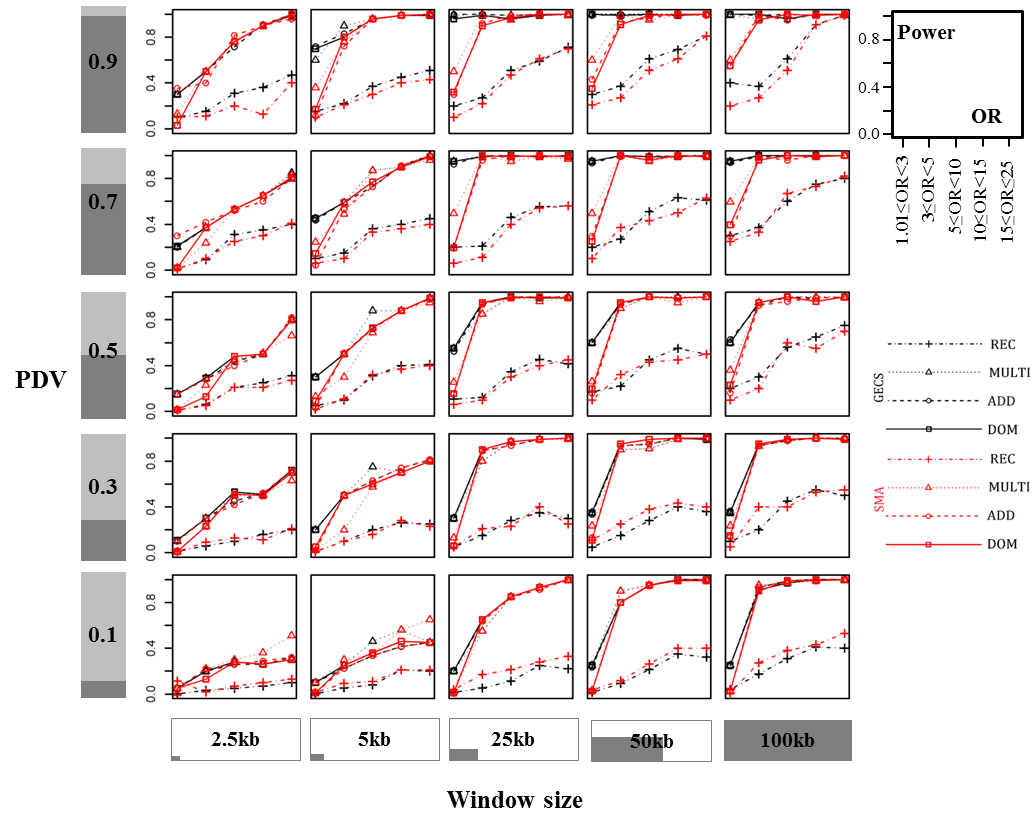
**

**Figure S13: Manhattan plot of single-marker analysis (SMA) for the AAMD data set. The y-axis is truncated at –log_10_(p)=25. The significance threshold (Table 2) and p-values were computed with the GECS software.**

**
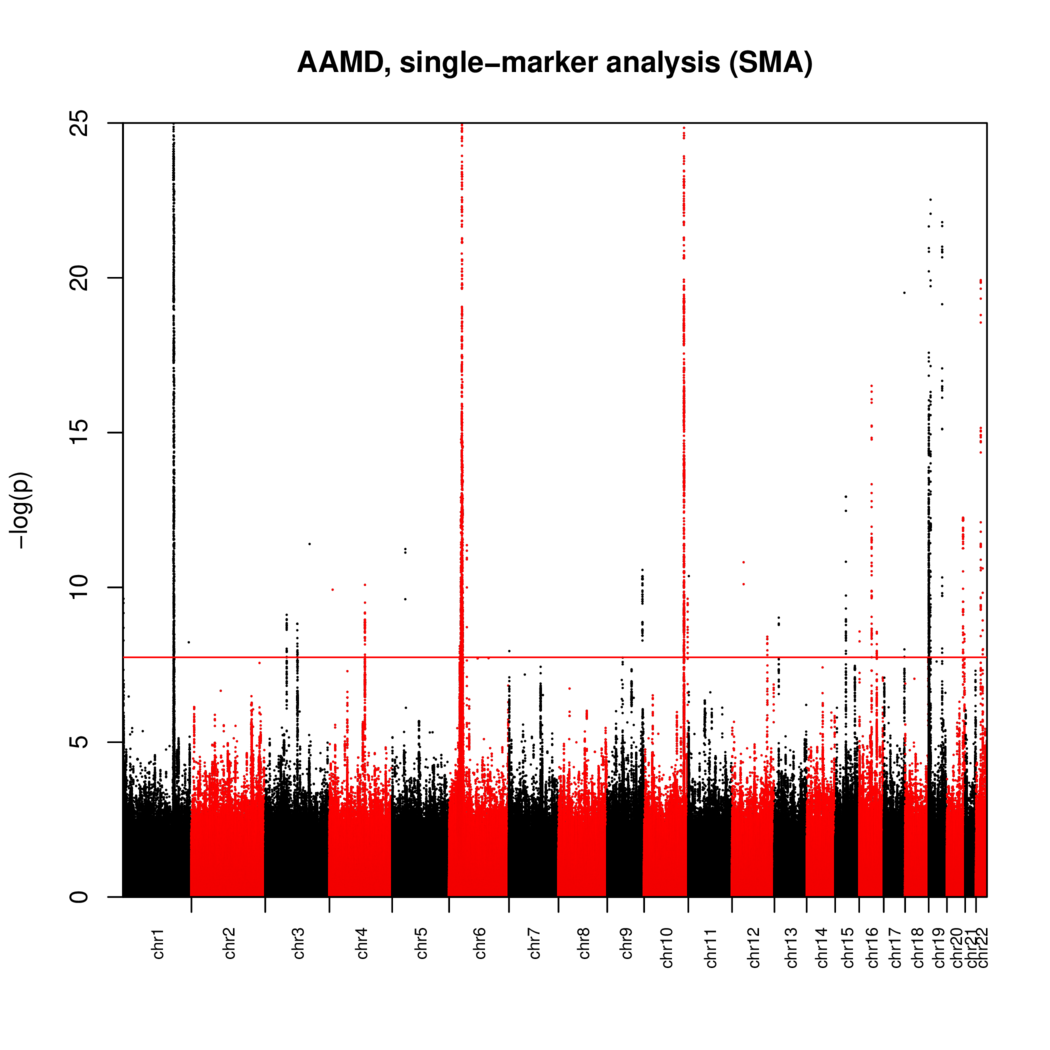
**

**Figure 14: Manhattan plot of bins at MAF_T_=0.01 for the AAMD data set. Dots represent the middle position of bins (average between the start and end positions). The y-axis is truncated at –log_10_(p)=25 and values of –log_10_(p)≤3 are omitted. The significance threshold for the combined the three MAF thresholds (Table 2) and p-values were computed with the GECS software.**

**
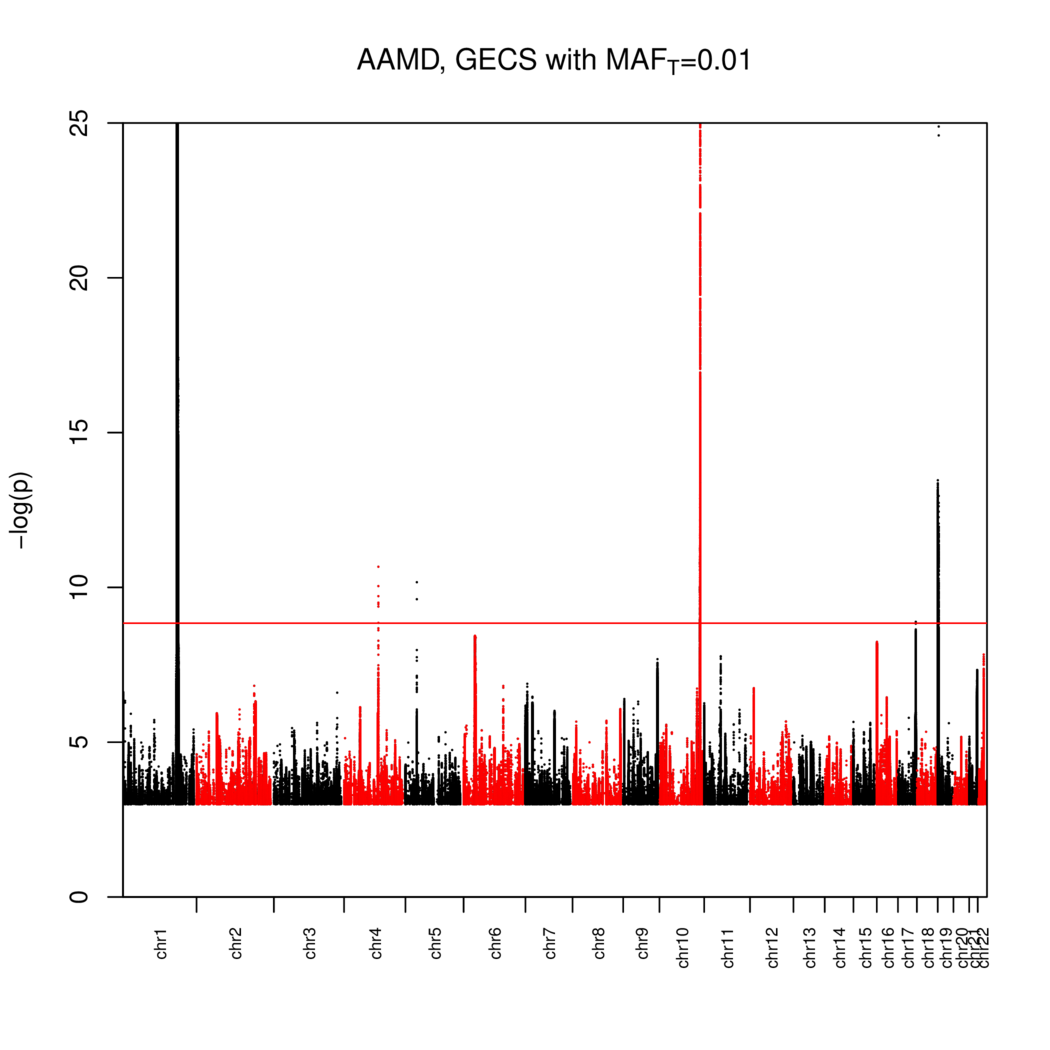
**

F**igure 15: Manhattan plot of bins at MAF_T_=0.03 for the AAMD data set. Dots represent the middle position of bins (average between the start and end positions). The y-axis is truncated at –log_10_(p)=25 and values of –log_10_(p)≤3 are omitted. The significance threshold for the combined the three MAF thresholds (Table 2) and p-values were computed with the GECS software.
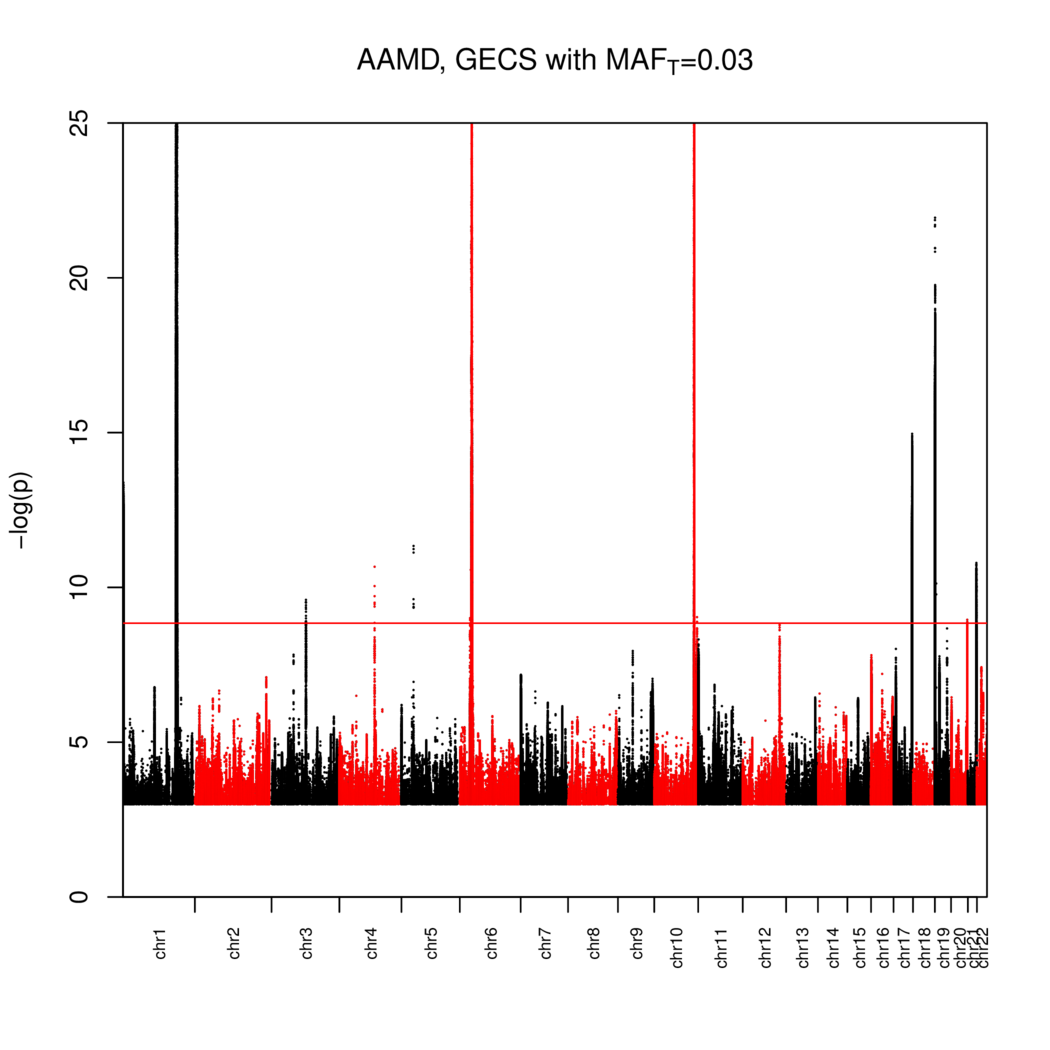
**

**Figure S16: Manhattan plot of bins at MAF_T_=0.05 for the AAMD data set. Dots represent the middle position of bins (average between the start and end positions). The y-axis is truncated at –log_10_(p)=25 and values of –log_10_(p)≤3 are omitted. The significance threshold for the combined the three MAF thresholds (Table 2) and p-values were computed with the GECS software.**


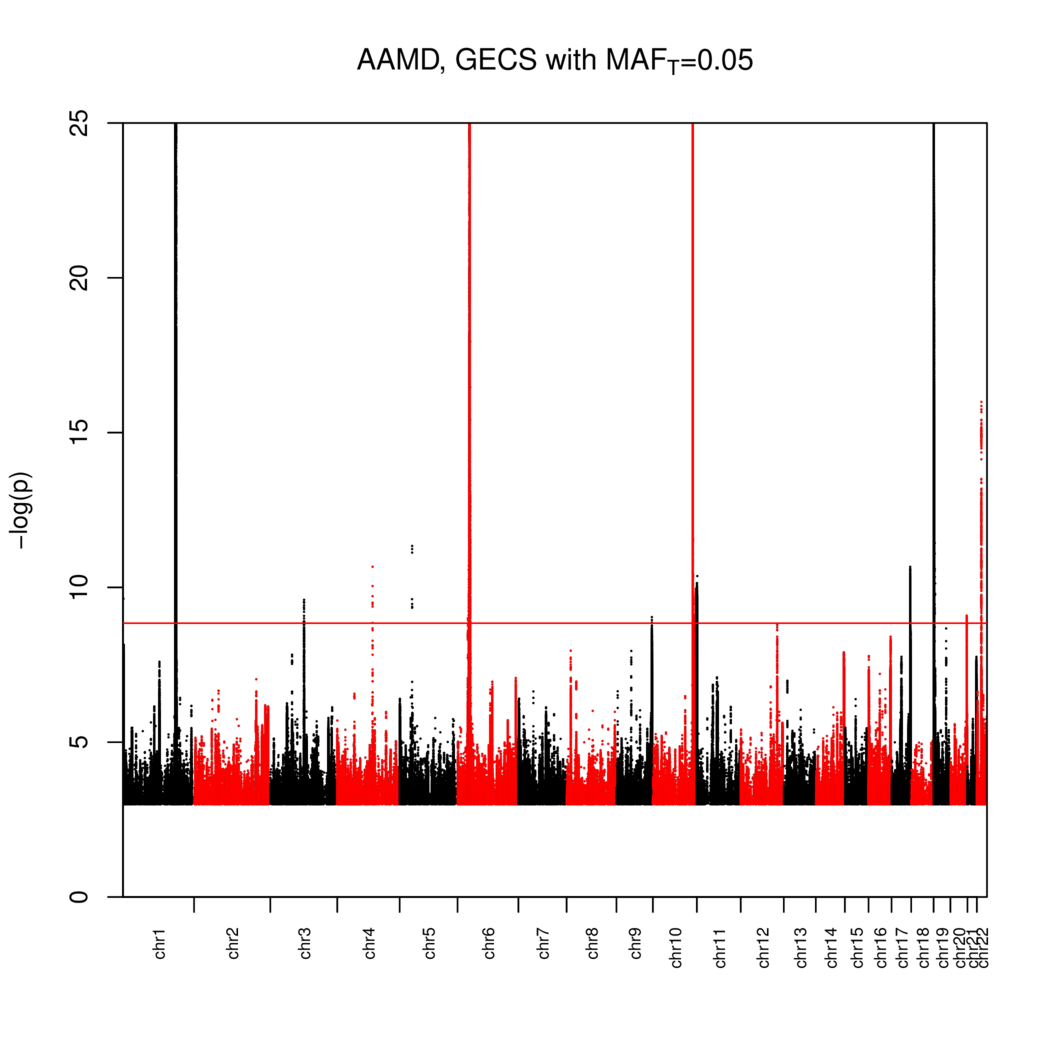


**Figure S17:**

The extended region [31,323,455-31,323,745] on chromosome 6 for NCT=2,657 in the AAMD data set. Numbers in cells represent the -log_10_ of p*-values. Cells with the lowest p*-values are shown in red.


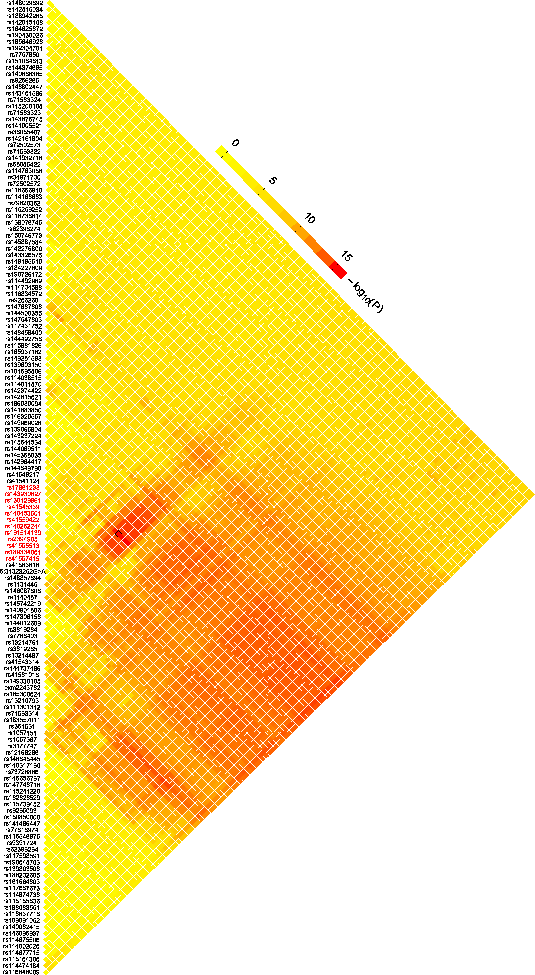


**Figure S18:**

The extended region [31,473,707-31,474,883] on chromosome 6 for NCT=2,657 in the AAMD data set. Numbers in cells represent the -log_10_ of p*-values. Cells with the lowest p*-values are shown in red.


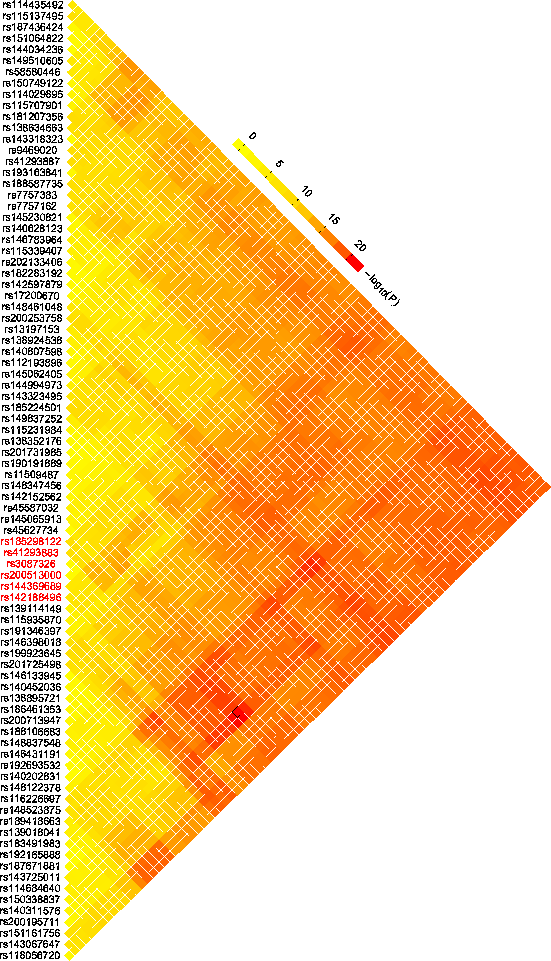


**Figure S19:**

The extended region [31,878,006-31,878,721] on chromosome 6 for NCT=2,657 in the AAMD data set. Numbers in cells represent the -log_10_ of p*-values. Cells with the lowest p*-values are shown in red.


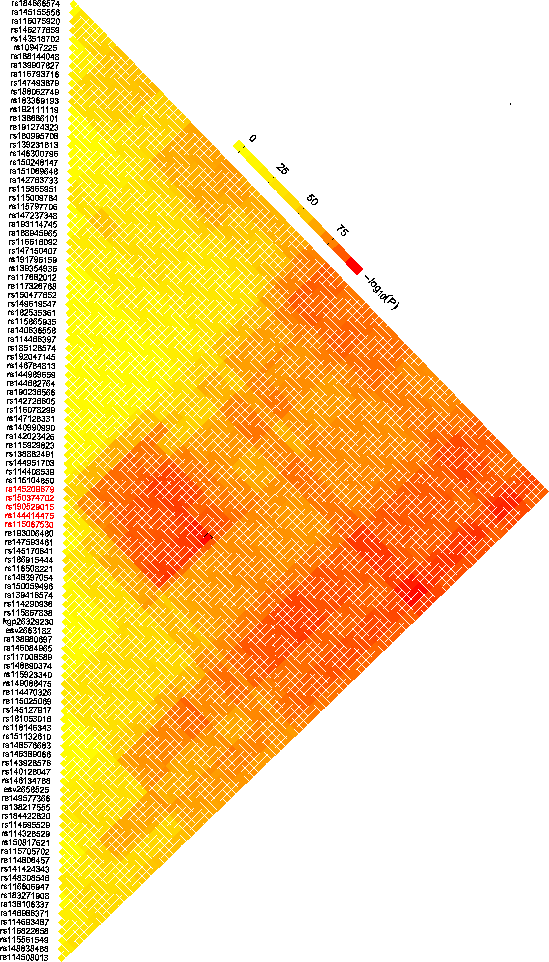


**Figure S20:**

The extended region [110,685,721-110,685,820] on chromosome 4 for NCT=1,611 in the AAMD data set. Numbers in cells represent the -log_10_ of p*-values. Cells with the lowest p*-values are shown in red.


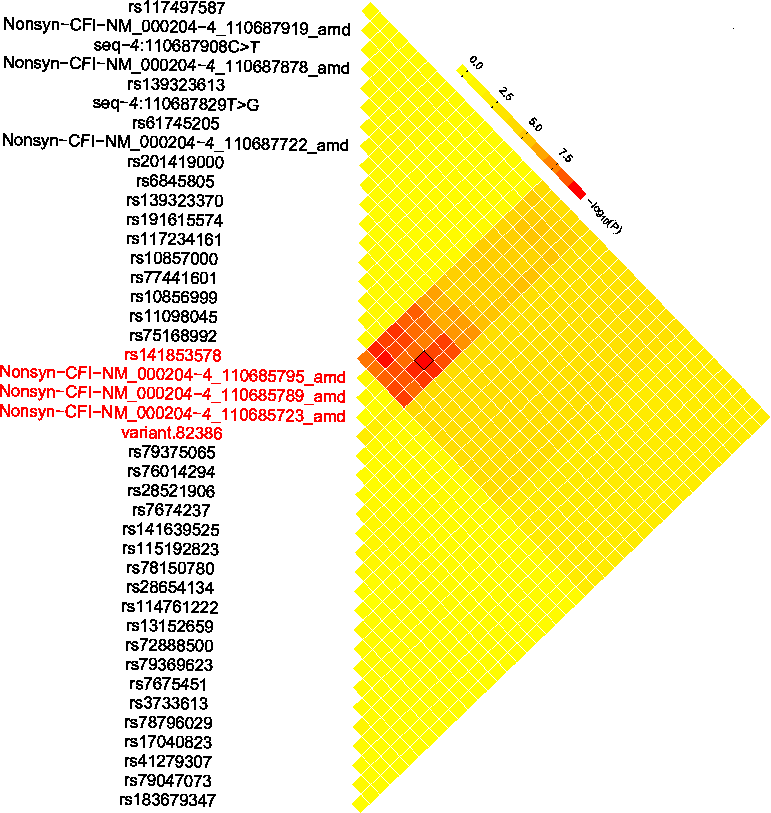


**Figure 21: Manhattan plot of single-marker analysis (SMA) for the SCZD data set. The significance threshold (Table 2) and p-values were computed with the GECS software.**

**
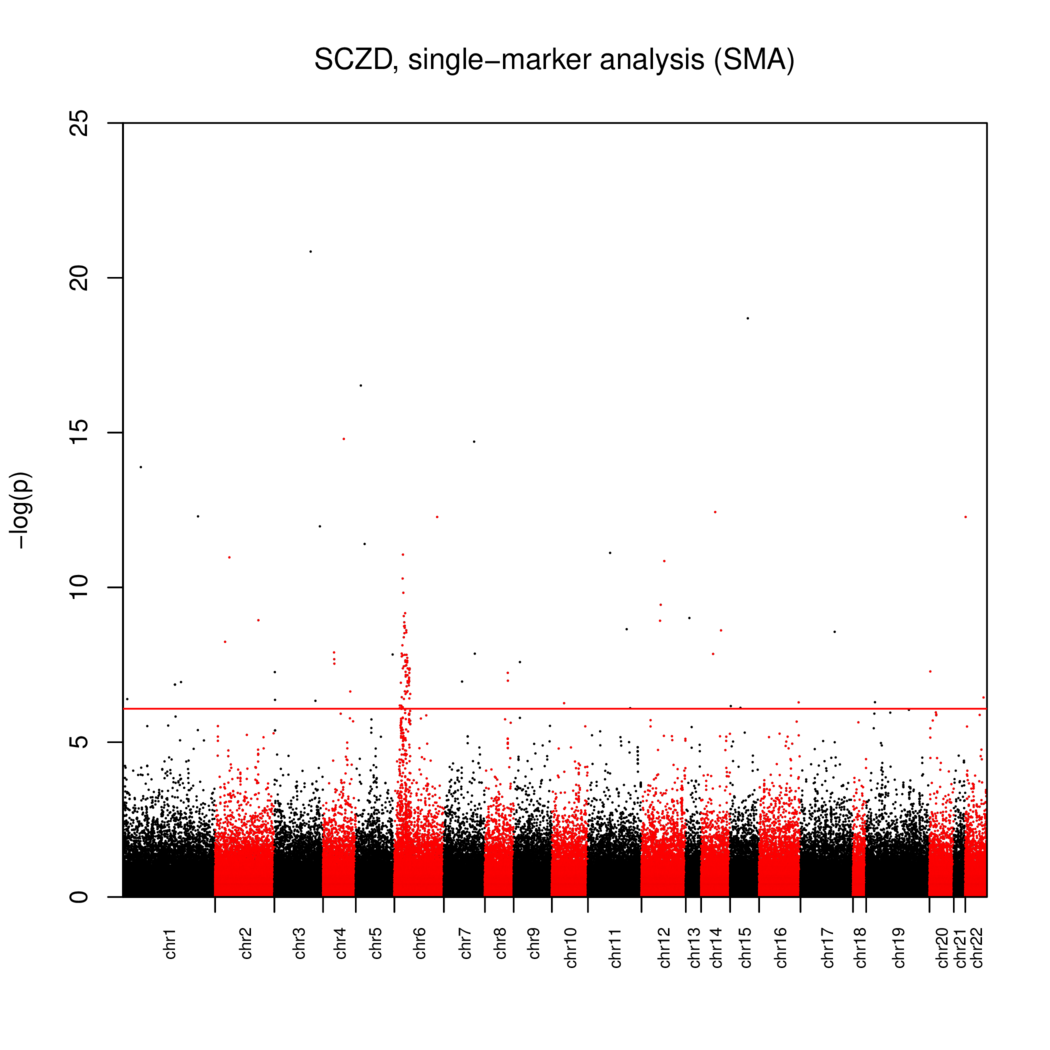
**

**Figure S22: Manhattan plot of bins at MAF_T_=0.01 for the SCZD data set. Dots represent the middle position of bins (average between the start and end positions). Values of –log_10_(p)≤2.5 are omitted. The significance threshold for the combined the three MAF thresholds (Table 2) and p-values were computed with the GECS software.**

**
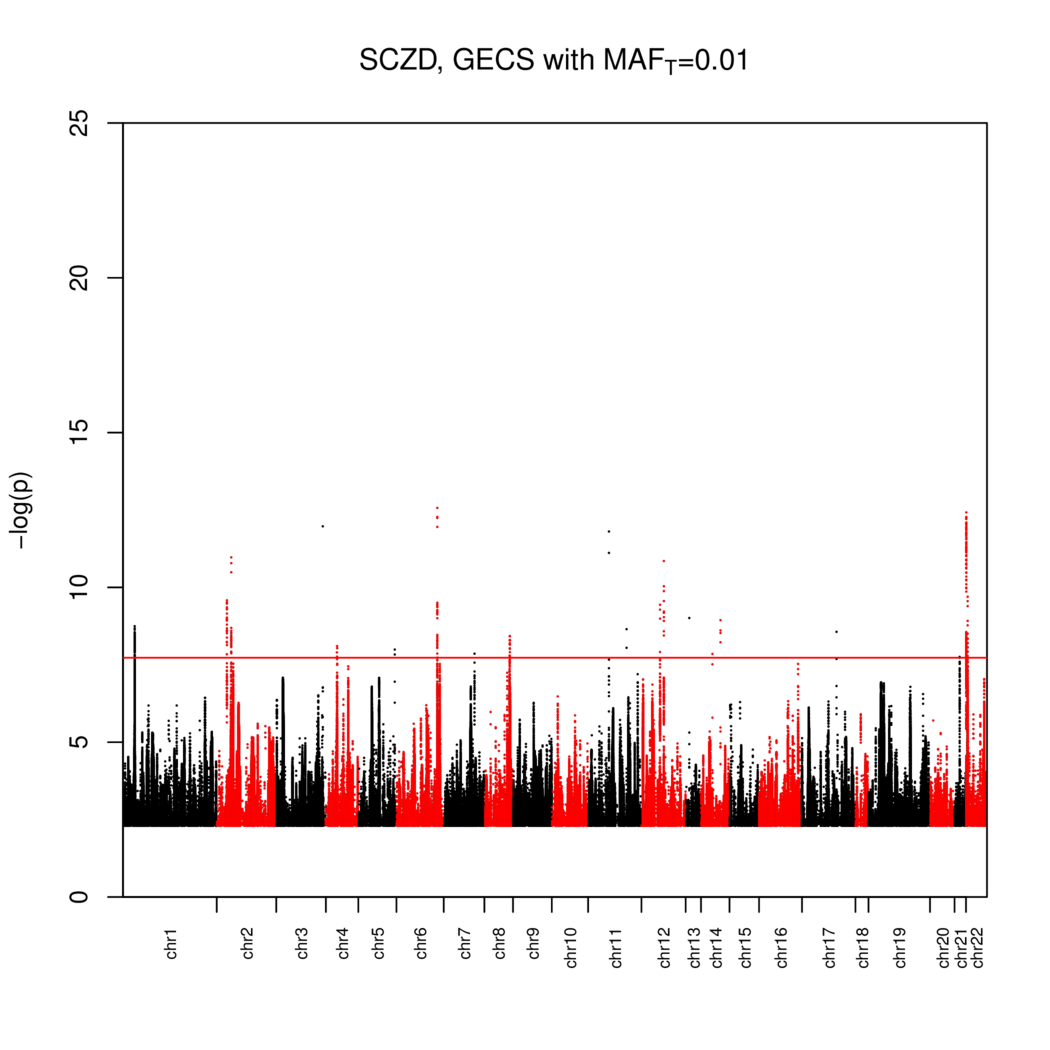
Figure S23: Manhattan plot of bins at MAF_T_=0.03 for the SCZD data set. Dots represent the middle position of bins (average between the start and end positions). Values of –log_10_(p)≤2.5 are omitted. The significance threshold for the combined the three MAF thresholds (Table 2) and p-values were computed with the GECS software.**

**
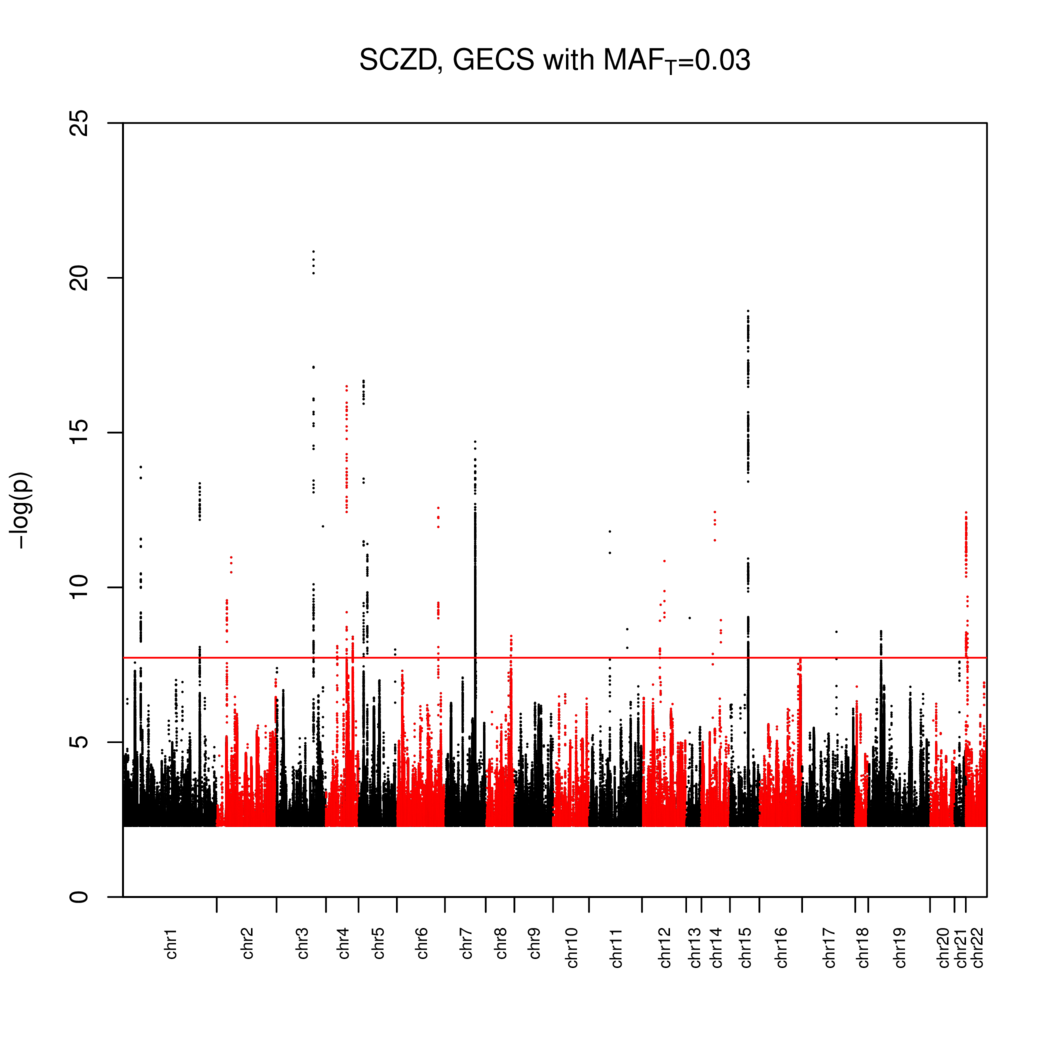
**

**Figure S24: Manhattan plot of bins at MAF_T_=0.05 for the SCZD data set. Dots represent the middle position of bins (average between the start and end positions). Values of –log_10_(p)≤2.5 are omitted. The significance threshold for the combined the three MAF thresholds (Table 2) and p-values were computed with the GECS software.**

**
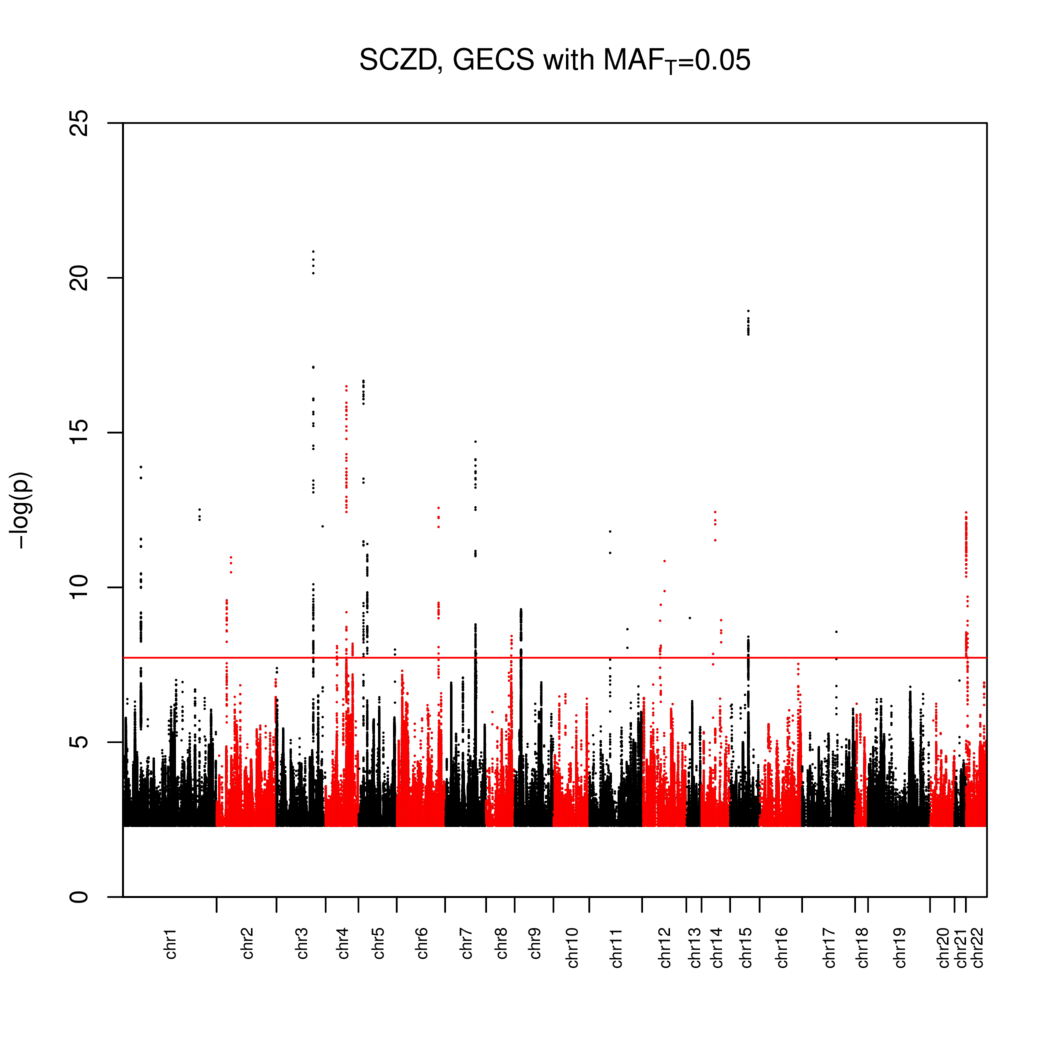
**

**Figure S25:**

The extended region [8,999,386-9,0s28,410] on chromosome 19 for NCT=636 in the SCZD data set. Numbers in cells represent the -log_10_ of p*-values. Cells with the lowest p*-values are shown in red.

## Supplementary Notes:

### Collapsing test (COLL)

Given a set of variants, the collapsing test COLL dichotomizes individuals by their carrier status, i.e. by presence of at least one minor allele. The resulting contingency table,

| #Individuals | Carrier | Non-carrier |
| --- | --- | --- |
| Affected | $N_{A}^{C}$ | $N_{A}^{N}$ |
| Unaffected | $N_{U}^{C}$ | $N_{U}^{N}$ |

can be evaluated with Pearson χ^2^ -test with the test statistic

$T_{COLL}=\frac{{(N_{A}+N_{U})(N_{U}N_{A}^{C}+N_{A}N_{U}^{C})}^{2}}{N_{A}N_{U}(N_{A}^{C}+N_{U}^{C})(N_{A}^{N}+N_{U}^{N})}$.

The odds ratios (OR) are obtained from the table by computing

$$OR=\frac{N_{A}^{C}N_{U}^{N}}{N_{U}^{C}N_{A}^{N}}.$$

A rare-variant analysis procedure requires a definition of ”rareness”, which is usually provided by setting a minor allele frequency threshold (MAF_T_) and considering all variants with minor allele frequencies below the threshold value as ”rare”.

1. **Genomic Exhaustive Collapsing Scan (GECS) algorithm**

The collapsing test COLL dichotomizes individuals upon their carrier status of at least one rare allele and applies a simple χ^2^ test to the resulting 2×2 contingency table in a case-control study design ([4](#_ENREF_4)) (Supplementary Note a)). The test only considers those variants whose minor allele frequency is below a pre-determined threshold (MAF_T_).We propose an exhaustive region-agnostic whole-genome scan approach that avoids pre-determination (and probable misspecification) of bins by considering all possible contiguous regions at a chromosome. The computational burden, usually prohibitive with human and other large genomes, is solved for COLL by identifying overlapping bins that are identical with respect to their variant carrier status in cases and controls, and skipping redundant computations. More specifically, consider a study with *n* variants with MAF less or equal to a fixed MAF_T_ being present at a chromosome. When conducting COLL, we can parameterize the data set using binary arrays $v_{1}, v_{2}, \ldots, v_{n}$ $v_{1}, v_{2}, \ldots, v_{n}$ $v_{1}, v_{2}, \ldots, v_{n}$, each of length *N*, where *N* denotes number of samples and the elements $v_{i,l}$ indicate the carrier status of the *l*-th individual (1=carrier, 0=non-carrier) at the i*-th* variant. Since the analyzed variants are rare, the arrays $v_{i}$ are sparse (most entries equal 0). We note that, although MAF_T_ is usually used to define “rareness”, in the setting of COLL, we use the number of carriers$N_{T}^{C}$, as COLL does not distinguish between homozygous and heterozygous rare-allele carries. By using the cutoff$N_{T}^{C}$, we define a fixed maximum count of 1’s in the arrays$v_{i}$. For a given MAF_T_, the corresponding $N_{T}^{C}$ can be estimated from the Hardy-Weinberg proportions as

$N_{T}^{C}=floor(N(2{MAF}_{T}-{MAF}_{T}^{2}))$.

For a bin comprising multiple variants, the logical OR operator yields the array with the carrier status of the individuals with respect to this bin from the constituent variant vectors:

$B_{ij}=v_{i}\bigvee v_{i+1}\bigvee\ldots\bigvee v_{j}$ ,

where *i* denotes the index of the start, or left, variant and *j* is the index of the end, or right, variant of the bin. Using the known affection status of the individuals in the study, contingency tables and test statistics ${T_{ij}=T(B}_{ij})$ can be obtained from binary arrays $B_{ij}$ in a computationally efficient way. The space of all combinations of bin start and end positions on a linear chromosome can be considered as a matrix *B* with elements$B_{ij}$ (Figure S1). Note that diagonal elements represent single-variants,$B_{ii}=v_{i}$, and $B_{1n}$ contains the carrier status with respect to all rare variants on the chromosome. Since *B* is symmetric ($B_{ij}=B_{ji}$), only the upper triangular part of *B* with *n(n + 1)/2* elements requires consideration.

Despite binary encoding and utilization of computationally efficient Boolean operators, computing *B* and performing association testing by brute force is not feasible for large data sets. However, the computational space can be considerably reduced, typically by several orders of magnitude. Our algorithm utilizes the fact that a given element$B_{ij}$ does not change by adding a variant that does not contribute new carriers, i.e.$B_{ij}=B_{i(j+1)}$. Likewise, removing the first variant$B_{ij}=B_{(i+1)j}$often results in an identical set of carriers and, subsequently, invariant contingency table and test statistic. Extrapolating this observation, we find that classes of overlapping bins can be found that are identical with respect to the collapsing test under variation of start and end coordinates. For such clusters of locally non-distinct bins (with respect to the binary representation), the test statistic needs to be computed only once. Therefore, the problem of computing the whole matrix *B* can be reduced to computing all locally distinct bins$B_{ij}$, in which a locally distinct bin represents a whole cluster of overlapping bins with identical contingency tables. Moreover, for practical purposes we do not need to keep track of the whole range of physical positions under which a bin remains invariant, it is sufficient to keep track of the coordinates of an arbitrary region representing the whole cluster. The set of locally distinct elements of * *can be found very efficiently by systematically traversing the matrix (w.l.o.g. row-wise) and exploiting properties of the logical OR operation that allow to perform early abandoning of a row while being sure that no distinct bin will be missed in the non-computed elements of the matrix. The crucial observation is that early abandoning can be performed if a bin $B_{ij}$ is equal to$B_{(i+1)j}$, i.e. the exact element below in the matrix. The condition $B_{ij}{=B}_{(i+1)j}$ ensures that $B_{i(j+k)}{=B}_{(i+1)(j+k)}$ for all *k ≥ 0*. In other words, if the condition is encountered once, it will be satisfied for the remainder of the row, which is a direct consequence of OR operator’s truth table. Intuitively, if the minor allele of the start variant *v_i_* is not carried by any individual that does not already carry at least one minor allele in$B_{(i+1)j}$, then *v_i_* will not contribute any new carriers to bins with an incremented end position ( *j+k* ). Therefore, all distinct bins not yet encountered in row *i* will be encountered in the next row, and may be in the following rows. This observation allows eliminating redundant computations inductively. Early abandoning can also be performed if a bin is found in which all individuals are carriers, i.e. the encoding binary array consists of only 1’s, $B_{ij}$= 𝟙, since the array remains “fully collapsed” for the remainder of the row. In pseudocode, the algorithm can be summarized as follows (see Supplementary Note (c) for comprehensive example of the algorithm and its implementation).

We implemented this algorithm in our publicly available analysis software GECS (https://github.com/ddrichel/GECS) using C++.

Finally, we note that although we perform our analysis under either one or multiple fixed thresholds$N_{T}^{C}$, it is possible to generalize the algorithm to consider all possible thresholds$N_{T}^{C}$, i.e. to simultaneously perform a variable threshold (VT) analysis (see Supplementary Note (d)). However, the algorithm of GECS combined with VT approach is in general not efficient enough to handle large-scale association studies. We therefore resort to applying GECS with three fixed$N_{T}^{C}$. Control of the FWER can be performed by combining the results from the three separate permutation runs under different MAF_T_s (see Supplementary Note (e)). Following ([5](#_ENREF_5)), we aim to perform at least 9,999 permutation replicates for significance testing at alpha=0.05, however for large data sets we sometimes only use 999 permutations.

### GECS with Variable Binning (VB), an example:

In the following, we present a small example for which we specify all steps of the algorithm.

Our goal is to find the locally distinct bins from the contiguous region that includes 8 variants in 4 individuals: $\left( v_{1}=\left( 1000 \right),v_{2}=\left( 0100 \right),v_{3}=\left( 0100 \right),v_{4}=\left( 0010 \right),v_{5}=\left( 0110 \right),v_{6}=\left( 1000 \right),v_{7}=\left( 1010 \right),v_{8}=\left( 0001 \right) \right)$

There are overall $8\left( 8+1 \right)/2=36$ combinations of start- end positions.

The full matrix $B$ is:

$$B=\left[ \begin{matrix} \left( 1000 \right) & \left( 1100 \right) & \left( 1100 \right) & \left( 1110 \right) & \left( 1110 \right) & \left( 1110 \right) & \left( 1110 \right) & \left( 1111 \right) \\ & \left( 0100 \right) & \left( 0100 \right) & \left( 0110 \right) & \left( 0110 \right) & \left( 1110 \right) & \left( 1110 \right) & \left( 1111 \right) \\ & & \left( 0100 \right) & \left( 0110 \right) & \left( 0110 \right) & \left( 1110 \right) & \left( 1110 \right) & \left( 1111 \right) \\ & & & \left( 0010 \right) & \left( 0110 \right) & \left( 1110 \right) & \left( 1110 \right) & \left( 1111 \right) \\ & & & & \left( 0110 \right) & \left( 1110 \right) & \left( 1110 \right) & \left( 1111 \right) \\ & & & & & \left( 1000 \right) & \left( 1010 \right) & \left( 1011 \right) \\ & & & & & & \left( 1010 \right) & \left( 1011 \right) \\ & & & & & & & \left( 0001 \right) \end{matrix} \right]$$

The algorithm executes as follows:

- Compute first bin to be analyzed:$B_{1,2}=v_{1}\vee v_{2}=\left( 1100 \right)$. This bin is locally distinct from both (1) $B_{1,1}=\left( 1000 \right)$and (2)$B_{2,2}=\left( 0100 \right)$.

- We keep track of the first identified distinct bin $B_{1,1}$by setting$I_{1}=\{1\}$.

- Next bin to be analyzed is$B_{1,3}=B_{1,2}\vee v_{3}=\left( 1100 \right)$. Variation of start and end positions identifies (1) $B_{1,3}$ as non-distinct from $B_{1,2}$ and (2) distinct from$B_{2,3}=\left( 0100 \right)$.

- Continuing with$B_{1,4}$, we find that $B_{1,4}=\left( 1110 \right)\neq B_{1,3}=\left( 1100 \right)\neq B_{2,3}=\left( 0110 \right)\Rightarrow B_{1,3}$ is a new distinct element: $I_{1}=\{1,3\}$.

- $B_{1,5}$: (1) $B_{1,5}=B_{1,4}=\left( 1110 \right)$; (2) $B_{1,5}\neq B_{2,5}$, continue.

- $B_{1,6}$: (1) $B_{1,6}=B_{1,5}=\left( 1110 \right)$, but (2) $B_{1,6}=B_{2,6}$ implies that $B_{1,7}=B_{2,7}$ and $B_{1,8}=B_{2,8}$, so that $B_{1,6},B_{1,7},B_{1,8}$ are not distinct from bins that will be encountered in the family of bins starting at $v_{2}$, the algorithm terminates for the first row of $B$ and the final $I_{1}$ is $I_{1}=\{1,3\}$, resulting in two distinct bins with start positions at $v_{1}$: $B_{1,1}$ and $B_{1,3}$.

-$B_{2,3}$: (1) and (2) are both satisfied: $B_{2,3}=B_{2,2}=B_{3,3}$, so that $I_{2}=\{\}$ and the algorithm terminates for row 2.

-$B_{3,4}$: (1) and (2) are both not satisfied: $B_{3,4}=\left( 0110 \right)\neq B_{3,3}=\left( 0100 \right)\neq B_{4,4}=\left( 0010 \right)\Rightarrow I_{3}=\left\{ 3 \right\}$.

-$B_{3,5}$: (1) and (2) satisfied: $B_{3,5}=B_{3,4}=B_{4,5}=\left( 0110 \right)$, terminate row.

-$B_{4,5}$: (1) $B_{4,5}=\left( 0110 \right)\neq B_{4,4}=\left( 0010 \right)\Rightarrow I_{4}=\left\{ 4 \right\}$, (2) $B_{4,5}=B_{55}=\left( 0110 \right)$, terminate row.

-$B_{5,6}$: (1) and (2):$B_{5,6}=\left( 1110 \right)\neq B_{5,5}=\left( 0110 \right)\neq B_{6,6}=\left( 1000 \right)\Rightarrow I_{4}=\left\{ 5 \right\}$.

-$B_{5,7}$: (1) $B_{5,7}=B_{5,6}=\left( 1110 \right)$, (2) $B_{5,7}\neq B_{6,7}$.

-$B_{5,8}$: (1) $B_{5,8}=\left( 1111 \right)\neq B_{5,7}=\left( 1110 \right)\Rightarrow I_{5}=\left\{ 5,7 \right\}$; $B_{5,8}=\left( 1111 \right)$ is a fully collapsed bin, terminate row.

-$B_{6,7}$: (1) $B_{6,7}=\left( 1010 \right)\neq B_{6,6}=\left( 1000 \right)\Rightarrow I_{6}=\left\{ 6 \right\}$, (2) $B_{6,7}=B_{7,7}$, terminate row.

-$B_{7,8}$: (1) and (2):$B_{7,8}\left( 1011 \right)\neq B_{7,7}=\left( 1010 \right)\neq B_{8,8}=\left( 1000 \right)\Rightarrow I_{7}=\left\{ 7,8 \right\}$

- Finally, include $B_{8,8}$: $I_{8}=\left\{ 8 \right\}$.

The algorithm finds the index sets$I=\left\{ \left\{ 1,4 \right\},\{\},\left\{ 3 \right\},\left\{ 4 \right\},\left\{ 5,7 \right\},\left\{ 6 \right\},\left\{ 7,8 \right\},\left\{ 8 \right\} \right\}$. This results in identification of all single-variant bins$B_{i,i}=v_{i}$, apart from $v_{2}$ (since$v_{2}=v_{3}$) and three bins consisting of two variants each:$B_{1,3}=v_{1}\vee v_{3}=\left( 1100 \right),B_{5,7}=v_{5}\vee v_{7}\left( 1110 \right),B_{7,8}=v_{7}\vee v_{8}=\left( 1011 \right)$. Only 10 locally distinct, non-fully-collapsed bins remain out of 36 possible bins.

We wish to re-emphasize at this point that the algorithm identifies *locally* distinct bins. It can be stated that all overlapping bins that are left after application of the algorithm are distinct. Non-overlapping bins, however, may have identical carrier status also after application of the algorithm. This is actually a desirable feature, since bins from chromosomal regions that are far apart can have identical carrier status. In such a case, it is important to know the locations of the identical bins, since they will point to different genes. In addition, the output of the analysis software shall report also the boundaries locally equivalent bins. While they can be neglected for computation, their exact location is of interest.

### GECS with variable threshold (VT):

Until now, the GECS method used a fixed threshold$N_{T}^{C}$, so that the number of carriers (i.e. the count of ‘1’s in each variant’s array) for each variant is below or equal to $N_{T}^{C}$:

*∀ i :* $N_{i}^{C}$= $N_{i}^{C}$(*v_i_*)=*l_i_* ≡ BitCount(*v_i_*) ≤ $N_{T}^{C}$ (1)

where the function ‘BitCount’ literally counts the number of ‘1’s in each variant’s array. We call$N_{k}^{C}$ the “level” of variant *k*.

The idea of VT analysis for a fixed bin is to find the ”optimal level” that minimizes the p-value among a set of the locally distinct bins, followed by correction for multiple testing.

For the GECS approach with VT, it turns out that it is possible to combine the VB and VT methods and to sift through all combinations of start position, end position and level in a computationally efficient manner by only considering one representative bin from each family of locally non-distinct bins. The objective is then to find all locally distinct bins for all combinations of three parameters.

We define $B_{i,j}^{l}$ (”$B_{i,j}$ at level *l*”) by combining all variants *v_k_* with *i ≤ k ≤ j* and$N_{k}^{C}$ *≤ l* using the logical ”OR”.

$B_{i,j}^{l}=\left\{ \begin{matrix} 0 & : & \left\{ N_{k}^{C} \right|i\leq k\leq j ; N_{k}^{C} \leq l\}=\emptyset\\ \bigvee_{k} v_{k}, k \in\left\{ i\leq k\leq j \right| N_{k}^{C}\leq l\} & : & otherwise \end{matrix} \right.$ (2)

Local non-distinctiveness at neighboring levels occurs if variants at level *l + 1* do not contribute carries *l* that are not in$B_{i,j}$ already:

$B_{i,j}^{l}=B_{i,j}^{l+1}$. (3)

We traverse the parameters *i, j* as in the case of VB analysis, with additional traversal of possible *l*-values for fixed *i, j*.

As in the case with a fixed threshold, we check for the conditions

$B_{i,j}^{l}=B_{i,j+1}^{l}$ (A1)

$B_{i,j}^{l}=B_{i+1,j}^{l}$ (A2)

during the traversal of parameters. If local non-distinctiveness is encountered during traversal of *l*, conditions (A1), (A2) have further consequences, compared to the fixed threshold scenario.

$B_{i,j}^{l}=B_{i,j+1}^{l}$ with *l ≤ l_j_* (B1)

$\Rightarrow B_{i,j}^{l+k}=B_{i,j+1}^{l} \forall k:0\leq k\leq N_{T}^{C}-l$_max_

$\Rightarrow$ stop traversing *l,* continue with next *j.*

This is a consequence of the fact that variant *v_j_* only contributes to bins at levels *l_j_* and higher. If (B1) is satisfied, we can stop traversing *l* for the current *j*. Likewise, if a fully collapsed bin is encountered, all bins at higher levels and larger *j* are guaranteed to be fully collapsed:

$B_{i,j}^{l}=B_{i+1,j}^{l}$ or $B_{i,j}^{l}=1$ (B2)

$\Rightarrow B_{i,j+k_{2}}^{l+k_{1}}=B_{i+1,j+k_{2}}^{l+k_{1}} \forall k_{1},k_{2}:0\leq k_{1}\leq N_{T}^{C}-l_{max};0\leq k_{2}\leq n-j$

$\Rightarrow$stop traversing *l*; for fixed *i*, the highest level we need to consider is *l_max_* *= l −* 1.

In other words, if *v_i_* does not contribute carriers to$B_{i,j}$, it will not contribute carriers to bins at either higher levels or bins with larger end coordinates.

Finally, local non-distinctiveness is possible at different levels for fixed *i* and *j*:

$B_{i,j}^{l}=B_{i,j}^{l+1} \forall l:l_{i}<l+1<l_{max}$ (B3)

$\Rightarrow$ eliminate $B_{i,j}^{l+1}$ .

This condition is trivially satisfied if$B_{i,j-1}^{l}=B_{i,j-1}^{l-1}$.

As opposed to *i* and *j*, most values of *l* do not correspond to potentially new distinct bins. For a fixed start position *i*, the lower bound of *l* is given by *l_i_*. The highest possible non-trivial level for bins with coordinates (*i, j*) is given by *l_max_* = max(*l_i_*, ...,*l_j_*), although it can be lower due to (B2). Furthermore, for a fixed *j > i*, potentially new locally non-distinct bins can be found at levels *l ≥* max(*l_i_*,*l_j_*) were either $B_{i,j-1}^{l}\neq B_{i,j-1}^{l+1}$, or at level *l_j_* if *l_j_* > *l_i_*.

To illustrate the algorithm, consider a minimal example with four individuals and four variants:

$$v_{1}=\left( 0011 \right),v_{2}=\left( 0010 \right),v_{3}=\left( 1011 \right),v_{4}=\left( 1000 \right)$$

Keeping *i*=1 fixed, the matrix $B_{1,j}^{l}$ has the following elements:

$$B_{1,j}^{l}=\left[ \begin{matrix} B_{1,1}^{4} & B_{1,2}^{4} & B_{1,3}^{4} & B_{1,4}^{4} \\ B_{1,1}^{3} & B_{1,2}^{3} & B_{1,3}^{3} & B_{1,4}^{3} \\ B_{1,1}^{2} & B_{1,2}^{2} & B_{1,3}^{2} & B_{1,4}^{2} \\ B_{1,1}^{1} & B_{1,2}^{1} & B_{1,3}^{1} & B_{1,4}^{1} \end{matrix} \right]$$

Based on just the levels of constituent variants, we can eliminate most of the elements in advance. As the level of the first variant is *l=*2, all bins containing *v_i_* are positioned at level 2 and above and all bins with *l*=1 are 0=(0000), by condition( B3). Furthermore, $B_{1,1}^{2}=B_{1,1}^{3}{=B}_{1,1}^{4}$, as all levels at position 1 only contain one variant with *l*=2, so that traversing the level does not contribute to addition of new variants. Likewise, $B_{1,2}^{2}=B_{1,2}^{3}=B_{1,2}^{4}$, as no variants at level 3 and 4 are available to modify $B_{1,2}^{2}$ at higher levels. By the same logic, $B_{1,3}^{3}=B_{1,3}^{4}$ and $B_{1,4}^{3}=B_{1,4}^{4}$.

We can also see that $B_{1,2}^{2}=B_{1,3}^{2}$, because the level of variant *v_3_*is *l*=3 and it therefore only contributes to level 3 and above. Setting all empty and trivially non-distinct bins to 0, we obtain only 5 potentially distinct elements:

$$B_{1,j}^{l}=\left[ \begin{matrix} 0 & 0 & 0 & 0 \\ 0 & 0 & B_{1,3}^{3} & B_{1,4}^{3} \\ B_{1,1}^{2} & B_{1,2}^{2} & 0 & B_{1,4}^{2} \\ 0 & 0 & 0 & 0 \end{matrix} \right]$$

$B_{1,1}^{2}$ is by definition $v_{1}=\left( 0011 \right).$ $B_{1,2}^{2}=v_{1}\bigvee v_{2}=(0011)$. Here, condition (B1) applies, and we can eliminate $B_{1,2}^{2}$ as locally non-distinct. On the other hand, $B_{1,3}^{3}=\left( 1011 \right)$ and (B2) is satisfied, with the consequence that $B_{1,4}^{3}$ =$B_{3,4}^{3}$ , and the bin $B_{1,4}^{3}$ will be encountered when we traverse the matrix $B_{3,j}^{l}$. The only remaining bin is indeed locally distinct at level 2, $B_{1,4}^{2}=(1011)$. Therefore, the only bins we consider for *i*=1 in this example are $B_{1,1}^{2}$ and $B_{1,4}^{2}$.

### Combined results and correction for multiple testing

Let us consider a study with large sample size *N*. To make the conducting of GECS on such studies more feasible and less time consuming, maybe we need to conduct the analysis separately on the 22 chromosomes. Moreover, we want to conduct GECS with different MAF_T_. As a result, we get for each of these sub-studies a separate list of maxT/minP of *n_0_* permutations. The combined result from all separate sub-studies must be also corrected for multiple testing. To hold the correction for multiple testing across the whole data, we have to conduct the maxT/minP approach for each permutation across all sub-studies, and subsequently the 5% level will be determined. In particular, for *m_0_* separate sub-studies and *n_0_* minP permutations conducted for each sub-study, the combined correction for multiple testing is

$$\alpha=0.95\_quantile \left( P \right),$$

where

$$P=\left( P_{1},P_{2},\ldots,P_{n_{0}} \right),$$

and

$$P_{i}=\min\left\{ P_{1}^{i}, P_{2}^{i},\ldots,P_{m_{0}}^{i} \right\}.$$

Note that for sub-studies$s_{1}, s_{2},\ldots, s_{m_{0}}$, the produced list of minP’s of *n_0_* permutations for the *j-th* sub-study will be $(P_{j}^{1},P_{j}^{2},\ldots,P_{j}^{n_{0}})$ and here the order of the permutation is very essential to get the aimed result.

In analogy, if we want to combine results from multiple MAF_T_, we have first to conduct the maxT/minP approach for each permutation, and subsequently the 5% level will be determined.

1. **Description of simulation studies**

**Genome-wide significance thresholds for rare-variant analysis**. The analysis of large genomic data sets involves simultaneous testing of many hypotheses, whose dependency structure may be unknown. Population history is a strong determining factor for this structure, as it induces specific patterns of genetic variation and allelic correlation. A genome-wide significance level α for analyzing rare variants in genomic data to control type I error will depend on the studied data set and the used statistical test. Given that the number of rare variants is high and that the number of possible bins grows quadratically with the number or rare variants, it can be expected that an exhaustive analysis of all possible contiguous subsequences will require smaller α than with tests of single common variants. For single-marker analysis, the problem of determining significance thresholds that control the FWER has been approached analytically ([6](#_ENREF_6)) and by using Monte-Carlo (MC) simulations ([7](#_ENREF_7)). In realistic scenarios, the analytical approach has to rely on approximations, possibly resulting in a conservative bias. On the other hand, the MC approach results in a quickly converging approximation of the exact thresholds, under the condition that the computational burden can be overcome ([8](#_ENREF_8)). In this study, we used the maxT/minP approach by ([9](#_ENREF_9)) in order to control the FWER at the 5% level, performing 999 permutations with respect to the case-control labels. When conducting an analysis with multiple MAF_T_, maxT/minP is obtained across MAF_T_ levels in each permutation, subsequently determining the combined significance level. This approach can be used also if the analysis is conducted in different chromosomes separately (for more details see Supplementary Note e)).

**Estimation of genome-wide significance levels.** In order to explore the distribution of the test statistic under the null hypothesis of no phenotypic association in a case-control study design, we simulated genome-wide WGS variant data sets representing populations of European (EUR) ancestry using HAPGEN2 software ([10](#_ENREF_10)) and the 99 CEU (Utah residents with Northwestern European ancestry) individuals from the 1000 Genomes Project Phase 3 as reference ([11](#_ENREF_11)) (ftp://ftp.1000genomes.ebi.ac.uk/vol1/ftp/release/20130502/; accessed October 2017). HAPGEN2 maintains the frequency distribution and linkage disequilibrium (LD) structure in the simulated data sets similar to that observed in the reference ([7](#_ENREF_7)). We repeatedly simulated data sets comprising 1,000 (“1k”), 5,000 (“5k”), 10,000 (“10k”) and 20,000 (20k”) individuals, respectively, totaling 500 replications for 20k and 1,000 replications for all other each sample sizes. Each data set was partitioned into “cases” and “controls” of equal size by random assignment. We then derived empirical thresholds for genome-wide significance by applying GECS under a threshold $N_{T}^{C}$ corresponding to minor allele frequencies of MAF_T_ = 0.01, 0.03 and 0.05, respectively. For comparison, COLL was applied to single-marker analysis (SMA) without imposing a frequency threshold and results were compared to those of GECS, delivering a base-line comparison for the performance of our method. Note that under COLL, the SMA analysis is equivalent to the genotypic χ^2^ test under the dominant model. The global 5% significance threshold for each combination of $N_{T}^{C}$ and sample size was estimated by the average 95% quantile for the test statistic across all replications.

**Power assessment.** In order to assess the power of GECS, we simulated autosomal local DNA sequences (2.5-100kb) under different disease models in a case-control study design. Simulating locally is justified by the expectation that true association signals will be small compared to whole chromosomes. Since GECS is guaranteed to find all distinct subsequences, we do not need to simulate association signals within whole genomes. Instead, we applied the exhaustive scan for association on the simulated subsequences and used the sample-size and variant-frequency specific genome-wide significance thresholds. Again, we also performed single-marker association (SMA) tests for comparison. More specifically, we repeatedly generated genomic regions of 100kb in forward-time simulated populations using SimRare ([12](#_ENREF_12)), while considering a range of disease models with multiple causal variants in these regions. First, we obtained 25 replications of variant pools from the forward-time simulations following a European simple bottleneck model ([13](#_ENREF_13)) as demographic history with an ancestral effective population size of N_anc_=7,895, a subsequent bottleneck with N_bot_=5,699 and a current effective population size of N_exp_=30,030, with an exponential growth duration of Gen_exp_=7,703 generations, a mutation rate of *μ=*2×10^-8^ and no recombination. In the second step, we repeatedly and randomly picked a variant pool and simulated a sample set comprising equal numbers of cases and controls under different disease models. Common variants (MAF≥0.05) in the simulations were given an OR of 1. Sample sets were parameterized in terms of: (i) proportion of functional detrimental rare variants (PDV), with values 0.1, 0.3, 0.5, 0.7, 0.9; (ii) proportion of considered neutral rare variants (PNV), with values 0.1, 0.3; (iii) intervals of odds ratios (OR) of detrimental rare variants as a function of their minor allele frequency (MAF), namely ]1, 3], ]3, 5], ]5,10], ]10,15], ]15,25]; (iv) mode of inheritance, namely, dominant (DOM), recessive (REC), additive (ADD), and multiplicative (MULTI); and (v) prevalence values *K* of 0.01 and 0.1 for the modeled trait (Table S1). Furthermore, we considered sample sizes of 1000, 10,000, and 20,000 individuals. We restricted our analysis to variants with MAF_T_ ≤ 0.01, corresponding to $N_{T}^{C}$equaling 19, 199 and 398 for the three sample sizes, respectively. For most combination of parameters and sample sizes, 100 simulations were performed and GECS as well as a SMA test were applied to test for phenotypic association (see Table S1 for more details). Obtained nominal p-values were compared against simulation-based sample-size and frequency-specific global significance thresholds.

**Feasibility of GECS.** We benchmarked GECS with respect to (i) the average reduction rate, defined as ratio of the numbers of actually calculated bins to those theoretically existing with a given set of variants, and (ii) the computational memory and time requirements. Since the number of variants included in the analyses depended on the applied MAF_T_ (MAF_T_=0.5 for SMA; MAF_T_=0.01, 0.03, 0.05, respectively, for GECS), it is useful to consider the number of performed tests for association in each study. The computation was conducted on Cheops, a high-performance cluster of University of Cologne, Germany (<https://rrzk.uni-koeln.de/cheops.html>). Nodes used for the calculations possess 24 GB RAM and CPUs with 2.66 GHz.

1. **Results of simulation studies**

**Global significance thresholds.** As expected, the GECS-specific thresholds for genome-wide significance were always more stringent than those for the single-marker analysis (SMA) and were inversely correlated with sample size and MAF_T_ (Figure S3-4).

The combined (adjusted for testing at the different MAF_T_) significance thresholds ranged between 7.35×10^-10^ and 2.59×10^-10^ for N=1,000 and N=20,000, respectively.

In real-world data, the combined significance thresholds were 1.87×10^-8^ for the exome dataset (SCZD) and 1.43×10^-9^ for the imputed data (AAMD). The much higher, and therefore less stringent, thresholds were expected due to the incomplete genomic coverage of the available real data. It is expected that in whole-genome, deeply sequenced studies the significance thresholds will be closer to the results obtained in the simulated data (Table 1).

We evaluated the statistical power of GECS, also in comparison to SMA, by simulating realistic case-control sequence data of 2.5-100kb regions under a variety of disease etiology models and sample sizes (Table S1). The exhaustive scan was applied to the simulated region and the significance of the strongest signal was determined using the global significance thresholds (Table 1). Here, we focus on results for PNV=0.3 since results for the different proportions of considered neutral variants (PNV) were similar.

**Power in models of rare diseases.** In small (N=1,000) case-control association studies of diseases with low prevalence (*K*=0.01), GECS performed substantially better than SMA across all considered inheritance modes in the presence of moderate to large proportions of detrimental rare variants (PDV) (Figure 2). In particular, the power ranged between 80% and 99% for high ORs (15≤OR≤ 25) with PDV≥0.3, whereas SMA’s power reached at most 40%. Even in the presence of only small OR values (i.e. 1<OR≤3), as would be expected in complex diseases ([14](#_ENREF_14)), the power of GECS ranged between 40% (PDV=0.5) and 95% (PDV=0.9). In contrast, the power of SMA exceeded 10% only for PDV>0.3 for all considered OR. Not unexpectedly, small PDV (PDV≤0.3) weakened the performance of GECS in comparison to SMA, where only few rare variants are considered in the study. With PDV=0.1, the power of GECS did not exceed 20% even for the largest OR interval [15, 25] where the power of SMA reached 80%.

For moderate sample sizes (N=10,000), both methods provided comparable power, although of the performance of GECS was clearly superior for small to medium OR and PDV>0.1 (Figure 3). For PDV=0.1, the SMA kept a slight advantage over GECS, although it was much less pronounced than in small-sample studies (N=1,000). A similar observation was made in studies with large sample size (N=20,000): power of both methods were higher for small OR values, but GECS remained much more powerful than SMA with moderate to large PDV values; only low proportions of detrimental rare variants favored the SMA (Figure S5). Increasing PDV always resulted in a power increase for GECS, while SMA remained underpowered (<50% for N=1,000 and 10,000) for small OR values even for the highest proportion of detrimental rare variants (PDV=0.9).

The impact of the assumed inheritance mode was broadly similar for the dominance (DOM), additive (ADD) and multiplicative (MULTI) genotypic inheritance modes, whereas the recessive mode (REC) resulted in the lowest power values for both GECS and SMA. The difference reached up to 40% for studies with small OR values (< 3). This is not surprising since homozygous carriers of rare variants are expected to be very rare. Extreme PDV values resulted in highly similar power values of about 10% (PDV=0.1) and about 100% (PDV=0.9) for all four modes, while intermediate values, ranging between 0.3 and 0.7, resulted in more pronounced differences between the recessive mode and the other three modes.

**Power in models of common diseases.** Applying GECS and SMA to diseases of higher prevalence (*K*=0.1) yielded some similarities to the results for rare diseases (*K*=0.01), but also some marked differences. In general, the power of both approaches slightly decreased.

Again, SMA performed better than GECS for PDV<0.3, but the power did not exceed 30% (SMA) and 10% (GECS), respectively, for the highest OR interval [15, 25]. Again, higher PDV values (PDV≥0.3) always yielded superior or at least equal power of GECS compared to SMA (Figures S6-7, S8). GECS attained about 100% power only for extreme PDV values (PDV=0.9) and extremely large OR values (≥15) and all but the recessive inheritance modes. Moderate sample size (N=10,000) strongly improved the power, ranging between 60-90% for studies with small to moderate OR values (1<OR≤5) and PDV ≥0.3 (Figure S7). Higher prevalence values also led to more nuanced results between the DOM, ADD and MULTI inheritance modes, although these remained marginal compared to the recessive mode. The moderate sample sizes provided substantial power (30-75%) even with small window sizes, although the power was consistently lower than for rare diseases (*K*=0.01) (Figures 3, S7). Increasing the sample size to N=20,000 resulted in an only negligible deviation from simulations with 10,000 individuals (Figures 3, S5, S7-8).

1. **Real-data analysis**

We applied GECS to two previously analyzed data sets using three different MAF thresholds and corrected for multiple testing using the combined significance thresholds. For each corrected p-value, in the results of the analysis of AAMD and SCZD data, the upper and lower limits of Wilson score binomial interval were calculated for 95% confidence interval. Subsequently we performed an in-depth analysis of the significant regions (Figure S2). The in-depth analysis was performed locally using SKAT ([15](#_ENREF_15)), as implemented in the SKAT R package (v 1.3.2.1), in two stages, the first of which was a locally exhaustive SKAT (“eSKAT”) with adjustment for age, sex and the first 10 principal components (p*-values). SKAT uses single-SNP score test statistics and a default weighting scheme based on the family of *Beta(x, α, β) ~ x^α-1^(1-x)^β-1^*distributions which assigns significantly higher weights to variants with low MAFs. In addition, candidate regions with p*-values that indicated possible association were re-analyzed with SKAT and common variants in physical proximity as additional covariates (p’-values). The last step was performed to assure that the association in the candidate region could not be explained simply due to linkage disequilibrium with an associated, common variant.

**Advanced age-related macular degeneration GWAS.** Advanced age-related macular degeneration (AAMD) is a neurodegenerative disease of the retina and the leading cause of blindness among people aged 55 years and older in the western world ([16](#_ENREF_16)). The disease is characterized by reduced retinal pigment epithelium (RPE) function and photoreceptor loss in the macula ([1](#_ENREF_1)). AAMD (late stage AMD) includes two morphological sub-types: endovascular AMD (“wet AMD”) and geographic atrophy (“dry AMD”) and typically preceded by clinically asymptomatic earlier stages of AMD ([17](#_ENREF_17)). A multitude of genes are reported to harbor common genetic variations associated with the disease (GWAS catalogue: https://www.ebi.ac.uk/gwas). Furthermore, the International AMD Genomics Consortium already examined the contribution of rare and common genetic variation and identified 52 independently associated common and rare variants distributed across 34 loci ([1](#_ENREF_1), [18](#_ENREF_18)). Besides these single-variant signals, gene-based enrichment of very rare coding variants (frequency < 0.1%) was observed in cases, implicating causal roles for *CFH, CFI,* and *TIMP3* in three of the known AMD risk loci. We obtained whole-genome imputed data from a case-control cohort study, where 14,566 patients with AAMD and 12,693 controls were included ([18](#_ENREF_18)) (data deposited in dbGaP database under dbGaP accession: phs001039.v1.p1). Besides more than 12 million variants, the study included 163,714 directly genotyped, mostly rare, protein-altering variants and 8,200 variants in known AAMD-associated loci ([1](#_ENREF_1)). We included only the 27,259 European samples from this study and restricted the phenotype to AAMD cases (Table S2-3). GECS was applied with three MAF thresholds, namely 0.01, 0.03 and 0.05, for defining rare variants. The final analysis was conducted by combining the results from all three thresholds and applying correction for multiple testing (see Methods). In a post-GECS analysis, we categorized all significant bins in blocks constituted by all overlapped bins in a region. Then we determined bins with the lowest p-values in each block, each of them being defined by their chromosomal positions and threshold MAF_T_. Subsequently, these bins were subject to further locally exhaustive association analysis.

**Schizophrenia Exome Study.** Gene-disruptive and putatively protein-damaging rare variants have been found to be enriched in individuals with schizophrenia ([19](#_ENREF_19), [20](#_ENREF_20)). Schizophrenia is a chronic debilitating mental disorder with a lifetime risk of about 0.7% and a heritability of 60–80% conferred by common and rare alleles at many loci ([21](#_ENREF_21), [22](#_ENREF_22)). Alleles associated with an extremely high risk are expected to be prevented from reaching even modest allele frequencies due to purifying selection ([23](#_ENREF_23)). Schizophrenia affects approximately 1% of the population worldwide and is characterized by thought abnormalities, hallucinations, delusions, and bizarre behaviors ([24](#_ENREF_24)). The genetic basis of this complex disorder was subject to numerous genome-wide association studies which continue to uncover common SNPs at novel loci (GWAS catalogue: <https://www.ebi.ac.uk/gwas>). One notable outcome of these large-scale genome-wide investigations is the degree of polygenicity, with thousands of genes and non-coding loci harboring risk alleles ([20](#_ENREF_20)). Recently, a Swedish study aimed to investigate the disruptive role of extremely rare variants in schizophrenia by sequencing a very large number of individuals from the same population. They analyzed the exome sequences of about 12,300 unrelated individuals from Sweden and informed this analysis with a much larger set of exome sequencing data from 45,376 individuals from multiple non-psychiatric cohorts ascertained by the Exome Aggregation Consortium ([19](#_ENREF_19)).

We analyzed WES variant data of 12,380 samples from the schizophrenia and bipolar disorder Swedish case-control cohort (data deposited in dbGaP database under dbGaP accession: phs000473.v2.p2). We included only those 10,898 samples that passed our quality control regarding population stratification (Table S5). Individuals with schizophrenia comprised 4,795 samples of the cases. Four $N_{T}^{C}$ values were considered for GECS analysis, namely 216, 642, 1059, and 3,910, corresponding to MAF_T_ of 0.01, 0.03, and 0.05, respectively. The post-GECS analysis was conducted analogously to AAMD.

### Extended results from AAMD data set

We applied GECS to the whole-genome imputed case-control data of the subset of samples with European ancestry and cases with AAMD from the international AMD genomics consortium (Table S2-3). The analysis was conducted for three MAF thresholds, and the genome-wide significance threshold in the combined study equalled 1.43×10^-09^ (Table 2, Figures S13-16). Previously, strong signals were found in genetic regions on chromosomes 1, 3, 4, 6, 19 and 22, mostly from common variants (Table S7, Figure S13). Recently, 16 other regions containing significant association signals with rare variants were reported ([1](#_ENREF_1)) (Table S8). GECS identified more than 100 genomic regions on chromosomes 1, 3, 4, 5, 6, 10 and 19, where bins of rare variants were found to be significantly associated with AAMD (locally validated by SKAT) (Tables 3, S9). The *CFI* gene was reported as associated with the AAMD phenotype and its subtypes in multiple studies ([25-27](#_ENREF_25)). We found a well-known rare variant in this region, rs141853578 (NC_000004.11:g.110685820C>T), on chromosome 4, to be significantly associated (p=3.1×10^-10^). This SNP was covered by bin 4.I (chr4:110,685,721-110,685,820bp) that included five rare variants (MAF_T_≤0.01) and was found to be significant with p =2.16×10^-11^, p’= 7.03×10^-10^ and OR = 3.43 (Figure S20). The bin was more significant than the single rare SNP, but the SNP possessed slightly larger OR of 3.8. Additionally, in the local eSKAT analysis, we found the bin 4.II (chr4:110,685,721-110,685,962bp) with 6 variants, including all variants of the bin 4.I, with a more significant p’-value of 3.31×10^-10^.

In general, regions that showed a strong association of common variants (MAF>0.05) with the phenotype were covered by huge bins, being detected as significant at MAF_T_ ≤ 0.05. This is because previously reported high-risk common variants may tag haplotypes that harbor rare variants included in these bins (some bins up to 5000 rare variants) (Table S13). As expected, local SKAT-based association testing with associated common variants as covariates led to disappearance of the association signal, confirming that the association seen in rare variants was only due to LD with nearby common variants (Table S13-14).

### Benchmarking of GECS

**Benchmarking of GECS for simulated data sets**. We investigated the impact of the number of included variants and of the sample size on the computation time and the required amount of memory for GECS. For real and simulated studies, this algorithm results in performance increase of multiple orders of magnitude and allows for exhaustive computation and correction for multiple testing via permutations. Since the number of variants included in the analyses depended on the applied MAF_T_ (MAF_T_=0.5 for SMA; MAF_T_=0.01, 0.03, 0.05, respectively, for GECS), it is useful to consider the number of performed tests of associations in each study. The study was conducted on Cheops, a high-performance cluster of university of Cologne, Germany (<https://rrzk.uni-koeln.de/cheops.html>). Nodes used for the calculations possess 24 GB RAM, 2.66 GHz each.

For small-sized studies (n=1,000), GECS performed on average 270 million (M) association tests, presenting the, number of the distinct bins in about 3 hours, requiring 3-4 GB (Table S18). In particular, GECS performed 276, 285, and 259 M association tests for 3.2, 5.3, and 6.2 M included variants, respectively. On the other hand, SMA performed 12.3 M association tests, representing the number of variants at MAF_T_=0.5, in 10 minutes, requiring 7 GB. For 3.2 M variants, COLL-based GECS reduced the number of actually performed tests to 276 M distinct bins down from a possible ~300,000 M bins that result from the *O(n^2^)* complexity of a naïve exhaustive search, i.e. a reduction by more than three orders of magnitude. Interestingly, the reduction rate became higher with increasing MAF_T_.

In general, the running time of both methods increased with increasing sample size, although to a lesser extent for SMA than for GECS. Moreover, the average reduction rate decreased slightly, which was more notable in for MAF_T_=0.01. The GECS analysis of the imputed whole-genome data of AMD, with 27,259 samples and round 900,000 variants, took about 3-4 hours as we analyzed each chromosome in parallel. On average, GECS required fewer computational resources than SMA, with memory usage ranging between 1-5 GB for GECS and about 14 GB for SMA (Table S19). The analysis of the schizophrenia WES data (~10,000 samples, ~300,000 SNPs) took at maximum 14 h for GECS and 6 h for SMA (Table S19).

**Benchmarking of GECS for real-world data sets**. GECS was found to be feasible for large data sets (Table 18). Analyzing the imputed whole-genome data of AMD, comprising 27,259 samples and round 900,000 variants, took less than 4 hours when analyzing each chromosome in parallel. On average, GECS required fewer computational resources than SMA, with memory usage ranging between 1-5 GB for GECS and about 14 GB for SMA (Table S19). The analysis of the schizophrenia WES data (~10,000 samples, ~300,000 SNPs) took at maximum 14h for GECS and 6 h for SMA (Table S19).

## References:

1. Fritsche LG, Igl W, Bailey JN, Grassmann F, Sengupta S, Bragg-Gresham JL, et al. A large genome-wide association study of age-related macular degeneration highlights contributions of rare and common variants. Nat Genet. 2016;48(2):134-43. Epub 2015/12/23.

2. Yan Q, Ding Y, Liu Y, Sun T, Fritsche LG, Clemons T, et al. Genome-wide analysis of disease progression in age-related macular degeneration. Human molecular genetics. 2018;27(5):929-40. Epub 2018/01/19.

3. Cipriani V, Leung HT, Plagnol V, Bunce C, Khan JC, Shahid H, et al. Genome-wide association study of age-related macular degeneration identifies associated variants in the TNXB-FKBPL-NOTCH4 region of chromosome 6p21.3. Human molecular genetics. 2012;21(18):4138-50. Epub 2012/06/15.

4. Li B, Leal SM. Methods for detecting associations with rare variants for common diseases: application to analysis of sequence data. American journal of human genetics. 2008;83(3):311-21. Epub 2008/08/12.

5. Kulldorff M. A spatial scan statistic. Communications in Statistics - Theory and Methods. 1997;26(6):1481-96.

6. Dickhaus T, Stange J. Multiple Point Hypothesis Test Problems and Effective Numbers of Tests for Control of the Family-Wise Error Rate. Calcutta Statistical Association Bulletin. 2013;65(1-4):123-44.

7. Pulit SL, de With SA, de Bakker PI. Resetting the bar: Statistical significance in whole-genome sequencing-based association studies of global populations. Genetic epidemiology. 2017;41(2):145-51. Epub 2016/12/19.

8. Atanassov E, Dimov IT. What Monte Carlo models can do and cannot do efficiently? Applied Mathematical Modelling. 2008;32(8):1477-500.

9. Westfall P. H. YSS. Resampling-Based Multiple Testing: Examples and Methods for P-Value Adjustment. New York: Wiley; 1993.

10. Su Z, Marchini J, Donnelly P. HAPGEN2: simulation of multiple disease SNPs. Bioinformatics. 2011;27(16):2304-5.

11. Auton A, Brooks LD, Durbin RM, Garrison EP, Kang HM, Korbel JO, et al. A global reference for human genetic variation. Nature. 2015;526(7571):68-74. Epub 2015/10/04.

12. Li B, Wang G, Leal SM. SimRare: a program to generate and analyze sequence-based data for association studies of quantitative and qualitative traits. Bioinformatics. 2012;28(20):2703-4. Epub 2012/08/24.

13. Boyko AR, Williamson SH, Indap AR, Degenhardt JD, Hernandez RD, Lohmueller KE, et al. Assessing the evolutionary impact of amino acid mutations in the human genome. PLoS genetics. 2008;4(5):e1000083. Epub 2008/06/03.

14. Bomba L, Walter K, Soranzo N. The impact of rare and low-frequency genetic variants in common disease. Genome biology. 2017;18(1):77. Epub 2017/04/30.

15. Wu MC, Lee S, Cai T, Li Y, Boehnke M, Lin X. Rare-variant association testing for sequencing data with the sequence kernel association test. American journal of human genetics. 2011;89(1):82-93. Epub 2011/07/09.

16. Smith W, Assink J, Klein R, Mitchell P, Klaver CC, Klein BE, et al. Risk factors for age-related macular degeneration: Pooled findings from three continents. Ophthalmology. 2001;108(4):697-704. Epub 2001/04/12.

17. Chakravarthy U, Wong TY, Fletcher A, Piault E, Evans C, Zlateva G, et al. Clinical risk factors for age-related macular degeneration: a systematic review and meta-analysis. BMC ophthalmology. 2010;10:31. Epub 2010/12/15.

18. Fritsche LG, Fariss RN, Stambolian D, Abecasis GR, Curcio CA, Swaroop A. Age-related macular degeneration: genetics and biology coming together. Annual review of genomics and human genetics. 2014;15:151-71. Epub 2014/04/30.

19. Genovese G, Fromer M, Stahl EA, Ruderfer DM, Chambert K, Landen M, et al. Increased burden of ultra-rare protein-altering variants among 4,877 individuals with schizophrenia. Nature neuroscience. 2016;19(11):1433-41. Epub 2016/10/28.

20. Purcell SM, Moran JL, Fromer M, Ruderfer D, Solovieff N, Roussos P, et al. A polygenic burden of rare disruptive mutations in schizophrenia. Nature. 2014;506(7487):185-90. Epub 2014/01/28.

21. McGrath J, Saha S, Chant D, Welham J. Schizophrenia: a concise overview of incidence, prevalence, and mortality. Epidemiologic reviews. 2008;30:67-76. Epub 2008/05/16.

22. Lichtenstein P, Yip BH, Bjork C, Pawitan Y, Cannon TD, Sullivan PF, et al. Common genetic determinants of schizophrenia and bipolar disorder in Swedish families: a population-based study. Lancet. 2009;373(9659):234-9. Epub 2009/01/20.

23. Zuk O, Schaffner SF, Samocha K, Do R, Hechter E, Kathiresan S, et al. Searching for missing heritability: designing rare variant association studies. Proceedings of the National Academy of Sciences of the United States of America. 2014;111(4):E455-64. Epub 2014/01/21.

24. Freedman R. Schizophrenia. The New England journal of medicine. 2003;349(18):1738-49. Epub 2003/10/31.

25. Alexander P, Gibson J, Cree AJ, Ennis S, Lotery AJ. Complement factor I and age-related macular degeneration. Molecular vision. 2014;20:1253-7. Epub 2014/10/30.

26. Seddon JM, Yu Y, Miller EC, Reynolds R, Tan PL, Gowrisankar S, et al. Rare variants in CFI, C3 and C9 are associated with high risk of advanced age-related macular degeneration. Nat Genet. 2013;45(11):1366-70. Epub 2013/09/17.

27. van de Ven JP, Nilsson SC, Tan PL, Buitendijk GH, Ristau T, Mohlin FC, et al. A functional variant in the CFI gene confers a high risk of age-related macular degeneration. Nat Genet. 2013;45(7):813-7. Epub 2013/05/21.
